# New liposidomycin congeners produced by *Streptomyces* sp. TMPU-20A065, anti-*Mycobacterium avium* complex agents with therapeutic efficacy in a silkworm infection model

**DOI:** 10.1038/s41429-024-00724-4

**Published:** 2024-05-08

**Authors:** Akiho Yagi, Mayu Fujiwara, Mayu Sato, Yuzu Abe, Ryuji Uchida

**Affiliations:** Division of Natural Product Chemistry, Faculty of Pharmaceutical Sciences, Tohoku Medical and Pharmaceutical University, 4-4-1 Komatsushima, Aoba-ku, Sendai, Miyagi 981-8558 Japan

**Keywords:** Drug development, Drug screening

## Abstract

Three new liposidomycin congeners (**1**, **2**, and **4**), together with 14 known liposidomycins (**3** and **5**–**17**), were isolated from the culture broth of *Streptomyces* sp. TMPU-20A065 as anti-*Mycobacterium avium* complex agents. The structures of liposidomycins were elucidated by spectroscopic analyses, including NMR and MS. Compounds **1**, **2**, and **4** belong to type-I liposidomycin-containing sulfate groups and methylglutaric acid, each with a different acyl side chain in the structure. Compounds **1**–**17** exhibited in vitro anti-*M. avium* and *M. intracellulare* activities with MIC values ranging between 2.0 and 64 μg ml^−1^. Furthermore, **1**–**17** exerted potent therapeutic effects in an in vivo-mimic silkworm infection model with ED_50_ values ranging between 0.12 and 3.7 μg larva^−1^ g^−1^.

## Introduction

*Mycobacterium avium* complex (MAC) infection, which is mainly caused by *M. avium* and *M. intracellulare*, is a nontuberculous mycobacterial pulmonary and intractable disorder, and the number of infected patients is increasing more than that of tuberculosis in developed countries [1, 2]. Symptoms are similar to those of tuberculosis, including weight loss, fever, fatigue, and night sweats [1], with slow progression and a less favorable prognosis. The first-line drug to treat MAC infections, clarithromycin (CAM), combines rifampicin and ethambutol. However, its therapeutic effect is insufficient and long-term administration for more than one year may be required, resulting in the emergence of drug-resistant bacteria, which is a problem [3]. Although amikacin liposome inhalation suspension was newly approved as a treatment for MAC infection in 2018 [4], its application is limited to patients for whom conventional combination therapies are not sufficiently effective. Therefore, there remains an urgent need to develop new candidates for the treatment of MAC infections with novel skeletal structures and different mechanisms of action from those of existing drugs. To resolve this issue, we developed an in vivo-mimic silkworm infection model with MAC based on our experience [5–13] and used it in the first screening of anti-MAC agents from microbial resources. We identified potent anti-MAC compounds with therapeutic effects, three new liposidomycin congeners (**1,** **2**, and **4**) and 14 known liposidomycins (**3** and **5**–**17**), in a culture broth of *Streptomyces* sp. TMPU-20A065. The fermentation, isolation, structural elucidation, and in vitro and in vivo antimycobacterial activities of liposidomycins were reported herein.

## Materials and methods

### General experimental procedures

UV spectra were recorded on a spectrophotometer (U-3310 UV-Visible spectrophotometer; Hitachi High-Technologies, Tokyo, Japan). IR spectra were recorded on a Fourier transform infrared spectrometer (FT/IR-4100; JASCO Co., Tokyo, Japan). Optical rotations were measured with a digital polarimeter (P-2300; JASCO Co., Tokyo, Japan). HRFAB-MS spectra were recorded on a mass spectrometer (JMS-MS 700, JEOL, Tokyo, Japan). Various NMR spectra were measured with a spectrometer (JNM-ECZ600R/S1; JEOL, Tokyo, Japan).

### Materials

Bovine serum albumin was purchased from FUJIFILM Wako Pure Chemical Industries (Osaka, Japan). CAM and Tween 80 were obtained from Tokyo Chemical Industries (Tokyo, Japan). Middlebrook 7H9 broth, malt extract, and yeast extract were supplied by Becton Dickinson and Company. Ehrlich meat extract was purchased from Kyokuto Pharmaceutical Co. (Tokyo, Japan). Solulys 095E was obtained from Oriental Yeast Co. (Tokyo, Japan). Oatmeal was purchased from Nippon Food Manufacturer (Hokkaido, Japan). The other compounds used were of special grade.

### Microorganisms

The following bacterial strains were used in the microdilution assay or silkworm infection assay: *M. avium* JCM 15430, *M. intracellulare* JCM 6384, *M. bovis* BCG Pasteur, and *M. smegmatis* NBRC 3207 for antimycobacterial activity; *Bacillus subtilis* NBRC 3134, *Staphylococcus aureus* NBRC 13276, *Escherichia coli* NBRC 3972, and *Pseudomonas aeruginosa* NBRC 13275 for antibacterial activity. Strains JCM 15430 and 6384 were provided by the Japan Collection of Microorganisms, RIKEN BRC, which is participating in the National BioResource Project of MEXT, Japan.

### Actinomycetal strain and identification

Liposidomycin-producing actinomycete strain TMPU-20A065 was isolated from soil collected at Yamaguchi city, Yamaguchi, Japan. Strain TMPU-20A065 was identified as *Streptomyces* sp. from its 16S rDNA sequence in a BLAST search by TechnoSuruga Laboratory (Shizuoka, Japan).

### Fermentation

An agar plate culture of strain TMPU-20A065 grown on ISP medium No. 2 (yeast extract 0.4%, malt extract 1.0%, glucose 0.4%, and agar 1.5%, pH 7.3) was inoculated into two 500-ml Erlenmeyer flasks containing 100 ml of a seed medium (potato starch 2.4%, yeast extract 0.5%, glucose 0.1%, peptone 0.3%, Ehrlich meat extract 0.3%, and CaCO_3_ 0.4%, adjusted to pH 7.0). Flasks were shaken on a rotary shaker (180 rpm) at 27 °C for 3 days. The seed culture (1.0 ml) was transferred into 180 × 500-ml Erlenmeyer flasks containing 200 ml of production medium (glucose 0.5%, Solulys 0.5%, KH_2_PO_4_ 0.5%, MgSO_4_·7H_2_O 0.5%, Oatmeal 0.5%, FeSO_4_·7H_2_O 0.001%, ZnSO_4_·7H_2_O 0.001%, MnCl_2_·4H_2_O 0.001%, CuSO_4_·5H_2_O 0.001%, and CoCl_2_·6H_2_O 0.001%, pH 7.0). Fermentation was performed on a rotary shaker (180 rpm) at 27 °C for 7 days.

### Isolation

Anti-MAC activity-guided isolation was performed step by step, leading to the isolation of **1**-**17** from the culture broth of *Streptomyces* sp. TMPU-20A065 (Supplementary Scheme 1). A 7-day-old culture broth (36 l) was centrifuged to separate the mycelia and supernatant, and the supernatant (14 l × 2) was applied to a Diaion HP-20 column (Mitsubishi Chemical Co., Tokyo, Japan, φ 65 × 220 mm, 400 ml) and eluted stepwise with 0, 50, and 100% methanol and 50% acetone (800 ml × 2 for each solvent). The 50% acetone fraction was concentrated to remove acetone, and the remaining aqueous solution was lyophilized to give a crude material (2.44 g). The material was dissolved in water and fractionated by medium-pressure liquid chromatography (column, ODS (Fuji Silysia Chemical Ltd., Nagoya, Japan, φ 28 × 80 mm); mobile phase, 0% (0–2 min), 0–100% (2–32 min), 100% (32–52 min) CH_3_CN-0.05% TFA; flow rate, 5.0 ml min^−1^; fractionation, 5 ml fraction^−1^). Fr. 27–31 (101.8 mg) was repeatedly purified by preparative high-performance liquid chromatography (HPLC: column, PEGASIL ODS SP100 (Senshu Scientific Co., Tokyo, Japan, i.d. 4.6 × 250 mm); mobile phase, 37% CH_3_CN-0.05% TFA; detection, UV at 210 nm; flow rate, 1.0 ml min^−1^). Under these conditions, **1** to **4** were eluted as peaks with retention times of 29, 31, 43, and 48 min, respectively (Fig. S1). Each eluate was concentrated *in vacuo* to yield pure **1** (1.12 mg), **2** (7.65 mg), **3** (0.20 mg), and **4** (1.21 mg) as a white powder. Fr. 32–39 (36.2 mg out of 86.3 mg) was repeatedly purified by preparative HPLC (column, PEGASIL ODS SP100 (i.d. 4.6 × 250 mm); mobile phase, 35–60% CH_3_CN-0.05% TFA gradient (0–50 min); detection, UV at 210 nm; flow rate, 1.0 ml min^−1^). Under these conditions, peaks A to I with retention times of 24, 26, 27, 29, 30, 31, 32, 40, and 43 min were repeatedly fractionated (Fig. S2). Peaks A, B, D, E, F, and I were concentrated *in vacuo*, respectively, to yield pure **5** (3.65 mg), **6** (3.35 mg), **9** (3.17 mg), **10** (0.97 mg), **11** (0.75 mg), and **17** (0.51 mg) as a white powder. Peak C (2.40 mg), including **7** and **8**, was re-chromatographed by preparative HPLC (column, Discovery HS F5 (Sigma-Aldrich Co. LLC., St. Louis, MO, USA, i.d. 4.6 × 250 mm); mobile phase, 35% CH_3_CN-0.05% TFA; detection, UV at 210 nm; flow rate, 1.0 ml min^−1^). Under these conditions, **7** and **8** were eluted as peaks with retention times of 42 and 51 min, respectively. Each eluate was concentrated *in vacuo* to yield pure **7** (0.33 mg) and **8** (1.49 mg) as a white powder. Peak G (2.60 mg), including **12** to **14**, was re-chromatographed by preparative HPLC (column, COSMOSIL πnap (Nacalai Tesque, Inc., Kyoto, Japan, i.d. 4.6 × 250 mm); mobile phase, 38% CH_3_CN-0.05% TFA; detection, UV at 210 nm; flow rate, 1.0 ml min^−1^). Under these conditions, **12** to **14** were eluted as peaks with retention times of 42, 44, and 47 min, respectively. Each eluate was concentrated *in vacuo* to yield pure **12** (0.60 mg), **13** (1.11 mg), and **14** (0.39 mg) as a white powder. Peak H (0.75 mg), including **15** and **16**, was re-chromatographed by preparative HPLC (column, Discovery HS F5 (i.d. 4.6 × 250 mm); mobile phase, 38% CH_3_CN-0.05% TFA; detection, UV at 210 nm; flow rate, 1.0 ml min^−1^). Under these conditions, **15** and **16** were eluted as peaks with retention times of 69 and 76 min, respectively. Each eluate was concentrated *in vacuo* to yield pure **15** (0.22 mg) and **16** (0.28 mg) as a white powder.

### Assay for antimycobacterial activity

#### In vitro broth microdilution method

The broth microdilution method was performed according to a previously established method [12, 14]. Mycobacterial strains were grown for 2 to 7 days at 37 °C in Middlebrook 7H9 broth (Middlebrook 7H9 broth 1.04%, Tween 80 0.05%, bovine serum albumin 0.5%, glucose 0.2%, and NaCl 0.085%) up to approximately 1.0 × 10^9^ CFU ml^−1^. Culture suspensions were then diluted 500 times with the same fresh broth. The suspension (95 μl) was added to each well of a 96-well microplate with or without test samples (5 μl in MeOH). The microplate was incubated at 37 °C for 2 to 7 days. Turbidity was assessed by measuring absorbance at 550 nm with an absorption spectrometer. The MIC value was defined as the lowest concentration of the test compounds at which bacterial growth was inhibited by 90% of control growth (no compound).

#### In vivo-mimic silkworm infection model

The silkworm infection model was performed according to a previously established method [13]. Fertilized silkworm eggs of *Bombyx mori* (Hu·Yo × Tukuba·Ne) were purchased from Ehime Sansyu (Ehime, Japan) and fed an artificial diet (Silk Mate 2 S; Nihon Nosan Kogyo, Kanagawa, Japan, and Silkmate; Katakura Industries, Tokyo, Japan) until the fourth-instar larval stage. Hatched silkworm larvae were raised by feeding an artificial diet containing antibiotics (Silk Mate 2 S, Nihon Nosan Kogyo, Kanagawa, Japan) in an incubator at 27 °C until the fourth molting stage. On the first day of the fifth-instar larval stage, silkworms were fed an antibiotic-free artificial diet (Silk Mate, Katakura Industries, Tokyo, Japan) until they weighed 2 g. On the second day, the *M. avium* or *M. intracellulare* suspension (2.5 × 10^7^ CFU larva^−1^ g^−1^ in 50 µl Middlebrook 7H9 broth) was injected into the hemolymph of silkworm larvae (2.0 g, *n* = 5) using a disposable 1-mL syringe with a 27-G needle (TERUMO, Tokyo, Japan), followed by an injection of test samples (50 µl in saline or 10% DMSO) within 30 minutes. Infected silkworms were raised without feed at 37 °C, and their survival rate was measured for 96 hours after the sample injection. ED_50_ values were defined as the amount of a sample required for a 50% survival rate normalized per 1 g of silkworm.

### Assay for antibacterial activity

The broth microdilution method was performed according to the guidelines of CLSI document M07-A09 [15]. Bacterial strains were grown overnight at 37 °C in Mueller–Hinton broth (Becton Dickinson, San Jose, CA, USA). Cultures were diluted with the same broth and adjusted to an optical density of 0.0548 at 550 nm (approximately 10^8^ CFU ml^−1^). Culture suspensions were then diluted 3000 times with the same fresh broth. The suspension (95 μl) was added to each well of a 96-well microplate with or without test samples (5 μl in MeOH). The microplate was incubated at 37 °C for 24 hours. Turbidity was assessed by measuring absorbance at 550 nm with an absorption spectrometer. The MIC value was defined as the lowest concentration of the test compounds at which bacterial growth was inhibited by 90% of control growth (no compound).

## Results

### Structural elucidation of liposidomycins

**Fig. 1:**
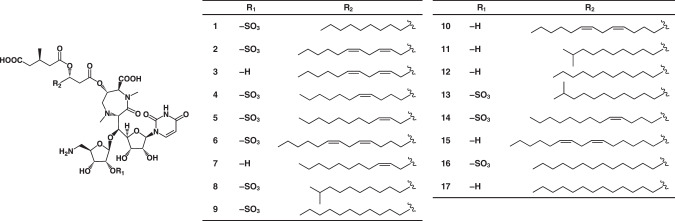
Structures of **1**–**17**

The structures of **3** and **5**–**17** were identified as known liposidomycins Y-III (**3**), Z-I (**5**), A-I (**6**), Z-III (**7**), B-I (**8**), C-I (**9**), A-III (**10**), B-III (**11**), C-III (**12**), isoH-I (**13**), G-I (**14**), K-III (**15**), M-I (**16**), and M-III (**17**), respectively, (Fig. 1) by comparing their various spectroscopic data, including NMR and MS experiments, with those reported in the literature [16–19].

The physicochemical properties of **1,** **2**, and **4** are summarized in Table 1. Compounds **1,** **2**, and **4** showed characteristic absorption maxima at 202–203 and 263–265 nm in UV spectra. Common IR absorption at 3393–3434, 2929–2961, and 1635–1691 cm^−1^ suggested the presence of alcohol, alkyl, and carbonyl moieties in their structures.

**Table 1:** Physico-chemical properties of **1,** **2**, and **4**

	1	2	4
Appearance	White powder	White powder	White powder
Molecular weight	981	1005	1007
Molecular formula	C_40_H_63_N_5_O_21_S	C_42_H_63_N_5_O_21_S	C_42_H_65_N_5_O_21_S
HR-FAB-MS (*m/z*)			
Calcd	982.3815 [C_40_H_63_N_5_O_21_S + H]^+^	1006.3815 [C_42_H_63_N_5_O_21_S + H]^+^	1008.3971 [C_42_H_65_N_5_O_21_S + H]^+^
Found	982.3837 [M + H]^+^	1006.3854 [M + H]^+^	1008.3962 [M + H]^+^
UV (MeOH) λ_max_ (log *ε*)	203 (4.1), 263 (3.8)	202 (4.1), 265 (3.8)	203 (4.1), 263 (3.8)
[α] _D_^25^ (*c* 0.1, MeOH)	23.7	16.0	22.3
IR (KBr) *ν*_max_ (cm^−1^)	3393, 2952, 2929, 1691, 1642, 1402, 1272, 1206, 1137, 1091	3409, 2958, 2931, 1687, 1635, 1402, 1275, 1204, 1135, 1081	3434, 2961, 2929, 1686, 1649, 1402, 1273, 1206, 1140, 1096

**Fig. 2:**
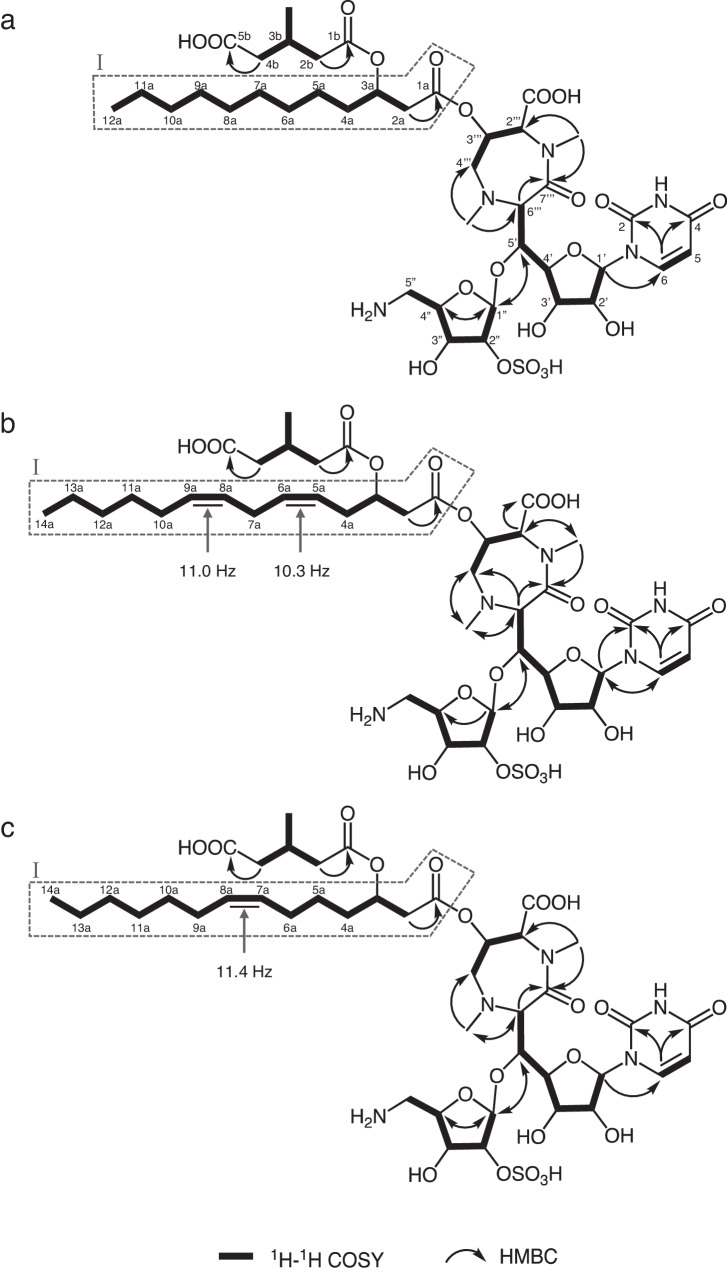
2D NMR experiments on new liposidomycins (a: **1**, b: **2**, and c: **4**)

Compound **1**: Its molecular formula was elucidated as C_40_H_63_N_5_O_21_S based on HR-FAB-MS measurements (*m/z* 982.3837 [M + H]^+^, *Δ*+2.2 mmu). The ^1^H and ^13^C NMR spectra of **1** in CD_3_OD (Table 2) were similar to those of liposidomycin C-I (**9**). In comparisons with the ^1^H and ^13^C NMR spectra of **1** and those of **9,** **1** appeared to possess the same basic structures as **9** containing 5’-substituted uridine, 5-amino-5-deoxyribose-2-sulfate, perhydro-1,4-diazepine, and 3-methylglutaric acid. Moreover, the molecular formula of **1** was smaller than that of **9** (C_42_H_67_N_5_O_21_S) by C_2_H_4_ (ethylene unit), suggesting that **1** possessed a shortened acyl side chain of **9** in a partial structure I. Furthermore, ^1^H-^1^H COSY correlations revealed the presence of a decanoic acid moiety in the partial structure I (Fig. 2). Therefore, the structure of **1** was confirmed by 2D NMR experiments, as shown in Fig. 2a, which fulfilled the molecular formula and degrees of unsaturation.

**Table 2:** ^1^H and ^13^C NMR chemical shifts in **1,** **2**, and **4**

No.	1		2		4	
	*δ*_C_	*δ*_H_ (*J* in Hz)	*δ*_C_	*δ*_H_ (*J* in Hz)	*δ*_C_	*δ*_H_ (*J* in Hz)
Uridine moiety						
2	^b^150.7		150.7		^b^150.7	
4	^b^165.0		164.9		^b^164.9	
5	101.3	5.85 (1H, d, *J* = 8.3)	101.3	5.84 (1H, d, *J* = 7.9)	^b^101.3	5.85 (1H, d, *J* = 7.6)
6	^b^140.5	7.74 (1H, d, *J* = 8.3)	140.5	7.74 (1H, d, *J* = 7.9)	^b^140.5	7.74 (1H, d, *J* = 7.6)
1'	^b^90.7	5.65 (1H, brs)	90.7	5.64 (1H, d, *J* = 2.1)	90.6	5.65 (1H, brs)
2'	74.5	4.08 (1H, brd, *J* = 4.8)	74.5	4.09 (1H, dd, *J* = 2.1, 5.2)	74.5	4.08 (1H, brd, *J* = 4.8)
3'	69.4	4.02 (1H, dd, *J* = 4.8, 7.6)	69.4	4.02 (1H, dd, *J* = 5.2, 7.9)	69.4	4.02 (1H, dd, *J* = 4.8, 7.6)
4'	^b^82.6	4.28 (1H, brd, *J* = 7.6)	82.5	4.27 (1H, dd, *J* = 1.7, 7.9)	^b^82.6	4.28 (1H, brd, *J* = 7.6)
5'	^b^75.9	4.31 (1H, brd, *J* = 9.3)	75.8	4.32 (1H, dd, *J* = 1.7, 9.3)	^b^76.0	4.32 (1H, brd, *J* = 9.0)
5-Amino ribose moiety						
1'	^b^109.6	5.14 (1H, s)	109.6	5.15 (1H, s)	^b^109.6	5.14 (1H, s)
2'	74.5	4.21 (1H, d, *J* = 4.1)	74.5	4.22 (1H, d, *J* = 4.1)	74.5	4.21 (1H, d, *J* = 4.1)
3'	74.5	4.54 (1H, m)	74.5	4.55 (1H, m)	74.5	4.54 (1H, m)
4'	^b^77.1	4.25 (1H, m)	77.0	4.26 (1H, m)	^b^77.2	4.25 (1H, m)
5'	^b^40.8	3.31 (2H, m)	40.8	3.31 (2H, m)	^b^40.8	3.30 (2H, m)
1,4-Diazepanone moiety						
1''-N-CH_3_	37.2	3.11 (3H, s)	37.1	3.12 (3H, s)	^b^37.2	3.11 (3H, s)
2''	63.4	4.58 (1H, d, *J* = 4.1)	63.7	4.62 (1H, d, *J* = 4.7)	^b^63.7	4.59 (1H, d, *J* = 4.1)
2''-CO_2_H	Not observed		169.5		Not observed	
3''	70.5	5.40 (1H, m)	71.0	5.38 (1H, m)	70.4	5.39 (1H, m)
4''	^b^55.8	3.16 (1H, m)	56.1	3.16 (1H, m)	^b^56.1	3.16 (1H, m)
		3.42 (1H, brd, *J* = 15.1)		3.43 (1H, brd, *J* = 15.5)		3.42 (1H, brd, *J* = 15.5)
5'''-N-CH_3_	^b^'36.0	2.44 (3H, s)	36.1	2.45 (3H, s)	^b^36.1	2.44 (3H, s)
6'''	^b^63.4	3.75 (1H, d, *J* = 9.3)	63.4	3.74 (1H, d, *J* = 9.3)	^b^63.4	3.74 (1H, d, *J* = 9.0)
7'''	^b^171.0		171.0		^b^171.0	
Acyl side chain moiety						
1a	^b^169.0		168.4		^b^169.0	
2a	^b^39.0	2.61 (1H, dd, *J* = 8.3, 15.3)	38.3	2.64 (1H, dd, *J* = 8.8, 15.8)	39.0	2.62 (1H, dd, *J* = 8.3, 15.8)
		2.68 (1H, dd, *J* = 4.1, 15.3)		2.70 (1H, dd, *J* = 4.1, 15.8)		2.68 (1H, dd, *J* = 4.1, 15.8)
3a	70.0	5.23 (1H, m)	70.2	5.24 (1H, m)	70.0	5.24 (1H, m)
4a	33.8	1.62 (2H, m)	31.4	2.36 (1H, m)	33.4	1.64 (2H, m)
				2.47 (1H, m)		
5a	24.9	1.30 (2H, m)	123.2	5.34 (1H, m)	25.1	1.37 (2H, m)
6a	^a^29.3	1.27 (2H, m)	131.8	5.51 (1H, m)	26.4	2.03 (2H, m)
7a	^a^29.2	1.27 (2H, m)	25.4	2.81 (2H, m)	128.6	5.31 (1H, m)
8a	^a^29.1	1.27 (2H, m)	127.0	5.30 (1H, m)	130.3	5.36 (1H, m)
9a	^a^29.0	1.27 (2H, m)	130.2	5.36 (1H, m)	26.9	2.01 (2H, m)
10a	31.7	1.27 (2H, m)	26.9	2.05 (2H, dq, *J* = 1.0, 7.2)	28.7	1.29 (2H, m)
11a	22.4	1.27 (2H, m)	29.1	1.35 (2H, m)	29.5	1.29 (2H, m)
12a	13.1	0.88 (3H, t, *J* = 7.2)	31.3	1.30 (2H, m)	31.6	1.30 (2H, m)
13a			22.3	1.30 (2H, m)	22.4	1.27 (2H, m)
14a			13.1	0.89 (3H, t, *J* = 7.2)	13.1	0.88 (3H, t, *J* = 6.9)
3-Methylglutaric acid moiety						
1b	^b^174.8		174.9		^b^174.9	
2b	40.5	2.17 (1H, m)	40.5	2.17 (1H, m)	40.5	2.17 (1H, m)
		2.33 (1H, m)		2.32 (1H, m)		2.32 (1H, m)
3b	27.4	2.35 (1H, m)	27.3	2.32 (1H, m)	27.4	2.32 (1H, m)
3b-CH_3_	18.8	0.99 (3H, d, *J* = 6.2)	18.8	0.99 (3H, d, *J* = 6.5)	18.8	0.99 (3H, d, *J* = 6.2)
4b	40.1	2.20 (1H, m)	40.1	2.20 (1H, m)	40.1	2.20 (1H, m)
		2.38 (1H, m)		2.38 (1H, m)		2.38 (1H, m)
5b	^b^172.0		172.2		^b^172.4	

^13^C (150 MHz) and ^1^H (600 MHz) spectra were taken on the JNM-ECZ600R/S1 (JEOL) in methanol-*d*_4_, and the solvent peaks were used as internal standards at 3.31 ppm for ^1^H NMR and at 49.0 ppm for ^13^C NMR

^a^Signals are interchangeable within the same letter

^b13^C NMR chemical shift values are assigned on the basis of 2D NMR correlations

Compound **2**: Its molecular formula was elucidated as C_42_H_63_N_5_O_21_S based on HR-FAB-MS measurements (*m/z* 1006.3854 [M + H]^+^, *Δ*+3.9 mmu), indicating that **2** was bigger than **1** by C_2_. In comparisons with the ^1^H and ^13^C NMR spectra (CD_3_OD) of **2** and those of **1** (Table 2), four *sp*^2^ methine signals: C-5a (*δ*_C_ 123.2, *δ*_H_ 5.34), C-6a (*δ*_C_ 131.8, *δ*_H_ 5.51), C-8a (*δ*_C_ 127.0, *δ*_H_ 5.30), and C-9a (*δ*_C_ 130.2, *δ*_H_ 5.36), were newly observed in **2**, suggesting that **2** possessed a C_2_ unit long polyunsaturated fatty acid moiety. Moreover, a ^1^H-^1^H COSY analysis revealed the presence of tetradec-5,8-dienoic acid in a partial structure I (Fig. 2b). Regarding the *cis*-*trans* configurations of two double bonds, the ^1^H-^1^H coupling constants between H-5a and H-6a (*J*_H5a-H6a_ = 10.3 Hz) and between H-8a and H-9a (*J*_H8a-H9a_ = 11.0 Hz) were elucidated by ^1^H-^1^H decoupling experiments, and both appeared to be *cis* forms (Fig. 2b). The structure of **2** was confirmed by 2D NMR experiments, as shown in Fig. 2b, which fulfilled the molecular formula and degrees of unsaturation.

Compound **4**: Its molecular formula was elucidated as C_42_H_65_N_5_O_21_S based on HR-FAB-MS measurements (*m/z* 1008.3962 [M + H]^+^, *Δ*-0.9 mmu), indicating that **3** was bigger than **1** by C_2_H_2_. In comparisons with the ^1^H and ^13^C NMR spectra (CD_3_OD) of **4** and those of **1** (Table 2), two *sp*^2^ methine signals, C-7a (*δ*_C_ 128.6, *δ*_H_ 5.31) and C-8a (*δ*_C_ 130.3, *δ*_H_ 5.36), were newly observed in **4**, suggesting that **4** possessed a C_2_ unit long monounsaturated fatty acid moiety. Moreover, a ^1^H-^1^H COSY analysis revealed the presence of 7-tetradecenoic acid in a partial structure I (Fig. 2b). Regarding the *cis*-*trans* configurations of the double bond, the ^1^H-^1^H coupling constants between H-7a and H-8a (*J*_H7a-H8a_ = 11.4 Hz) were elucidated by ^1^H-^1^H decoupling experiments, and appeared to be the *cis* form (Fig. 2c). The structure of **4** was confirmed by 2D NMR experiments, as shown in Fig. 2c, which fulfilled the molecular formula and degrees of unsaturation.

The stereochemistries of **1,** **2**, and **4** were elucidated by ROESY experiments (Fig. S3, S9, S15, and S21) and ^1^H-^1^H coupling constants (Table 2), and the results obtained showed that the relative stereochemistries of **1,** **2**, and **4** were in good agreement with those of liposidomycin B-I (**8**) in the literature [17]. In addition, the optical rotations of **1,** **2**, and **4** have the same value as that of **8** ([α]_D_^24^ (*c* 0.4, H_2_O) = 17.3°) in the literature [16].

### In vitro antimicrobial activity using the microdilution method

The MIC values of **1**–**17** against eight test microorganisms in the microdilution method are listed in Table 3. Compounds **1**–**17** exhibited anti-MAC activity against *M. avium* and *M. intracellulare*, with MIC values ranging between 2.0 and 64 μg ml^−1^. Moreover, **5**–**7,** **9**–**12,** **15**, and **17** exhibited weak antimycobacterial activity against *M. smegmatis* and/or *M. bovis* BCG, with MIC values ranging between 16 and 64 μg ml^−1^. On the other hand, **1**–**17** did not exhibit antibacterial activity, even at 64 µg ml^−1^, against *B. subtilis*, *S. aureus*, *E. coli*, or *P. aeruginosa*.

**Table 3:** MIC and ED_50_ values of compounds **1**–**17**

Microorganism		1	2	3	4	5	6	7	8	9	10	11	12	13	14	15	16	17	CAM
*M. avium*	MIC	64	16	32	32	8.0	8.0	8.0	8.0	8.0	4.0	16	32	4.0	8.0	64	16	8.0	0.098
	ED_50_	0.26	0.52	0.68	0.95	0.44	0.70	0.12	0.16	0.70	0.76	0.72	2.7	0.95	0.76	NT	0.84	2.7	23
*M. intracellulare*	MIC	4.0	8.0	8.0	4.0	4.0	4.0	2.0	2.0	2.0	2.0	64	8.0	4.0	8.0	64	16	4.0	0.012
	ED_50_	1.2	0.26	0.76	1.1	0.52	1.2	0.90	0.44	1.5	0.52	3.7	0.84	0.44	0.20	NT	1.5	0.72	42
*M. bovis* BCG	MIC	>64	>64	>64	>64	>64	>64	64	>64	>64	64	>64	64	>64	>64	64	>64	64	NT
*M. smegmatis*	MIC	>64	>64	>64	>64	64	64	>64	>64	32	16	64	64	>64	>64	>64	>64	64	NT
*B. subtilis*	MIC	>64	>64	>64	>64	>64	>64	>64	>64	>64	>64	>64	>64	>64	>64	>64	>64	>64	NT
*S. aureus*	MIC	>64	>64	>64	>64	>64	>64	>64	>64	>64	>64	>64	>64	>64	>64	>64	>64	>64	NT
*E. coli*	MIC	>64	>64	>64	>64	>64	>64	>64	>64	>64	>64	>64	>64	>64	>64	>64	>64	>64	NT
*P. aeruginosa*	MIC	>64	>64	>64	>64	>64	>64	>64	>64	>64	>64	>64	>64	>64	>64	>64	>64	>64	NT

MIC (µg ml^−1^), ED_50_ (µg larva^−1^ g^−1^), CAM: clarithromycin

### In vivo-mimic anti-MAC activity using the silkworm infection model

**Fig. 3:**
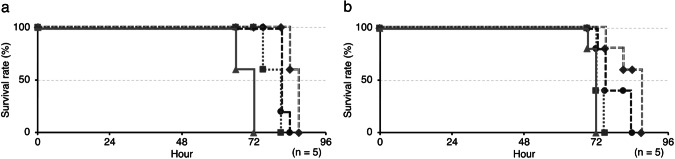
Therapeutic effects of **1** in the silkworm infection model with (**a**) *M. avium* and (**b**) *M. intracellulare*. ◆: 4, ●: 2, ■: 1, ▲: 0 µg larva^−1^ g^−1^

Compounds **1**–**17**, except for **15**, were evaluated in the silkworm infection model with *M. avium* and *M. intracellulare* (*n* = 5), and their ED_50_ values are summarized in Table 3. As an example, the therapeutic effects of **1** against *M. avium* and *M. intracellulare* are shown in Fig. 3a and b, respectively. All infected silkworms without a compound (control) died within 72 hours. When **1**–**17** were administered to silkworms infected with *M. avium*, potent therapeutic effects were confirmed in a dose-dependent manner with ED_50_ values ranging between 0.12 and 2.7 μg larva^−1^ g^−1^. Similarly, **1**–**17** exerted potent therapeutic effects in the silkworm infection model with *M. intracellulare*, with ED_50_ values ranging between 0.44 and 3.7 μg larva^−1^ g^−1^. Incidentally, **1**–**17** alone did not exhibit any toxicity towards silkworms at a dose of 32 µg larva^−1^ g^−1^ for 96 hours (data not shown).

## Discussion

In the present study, three new liposidomycins congeners (**1,** **2**, and **4**), together with 14 known liposidomycins (**3** and **5**–**17**), were isolated from *Streptomyces* sp. TMPU-20A065 as selective anti-MAC compounds with therapeutic effects in the silkworm infection model. The in vivo-mimic silkworm infection model has several advantages as an alternative mammalian model, such as similar therapeutic effects and pharmacokinetics of antibiotics with the mouse model and no ethical issues [20, 21]. Moreover, this model evaluates the therapeutic efficacy of test samples in a few days. Therefore, we have been adapting this model in the early stages of various screenings for antibiotics of microbial origin [5–13], which has resulted in the identification of new liposidomycin congeners in the present study.

Liposidomycins are classified as types I–IV based on the presence or absence of a 3-methylglutaric acid and sulfate group [18]. Consequently, **1,** **2,** **4**–**6,** **8,** **9,** **13,** **14**, and **16** belong to type I liposidomycins with both 3-methylglutaric acid and a sulfate group, while **3,** **7,** **10**–**12,** **15**, and **17** belong to type III liposidomycins with 3-methylglutaric acid and no sulfate group; therefore, new congeners **1,** **2**, and **4** are type-I liposidomycins with different acyl side chains in the partial structure I (Fig. 2). However, the production of types II and IV liposidomycins without 3-methylglutaric acid was not detected in the culture broth of *Streptomyces* sp. TMPU-20A065.

In 1985, liposidomycins were isolated from *Streptomyces griseosporeus* RK-1061 as uridyl liponucleoside antibiotics exhibiting antibacterial activity, including against *M. phlei* [16], and were found to inhibit phospho-*N*-acetyl muramyl pentapeptide transferase (MraY) in the peptidoglycan synthesis of *E. coli* [22]. Caprazamycin B, which is also a uridyl liponucleoside acid antibiotic, was isolated from *Streptomyces* sp. in 2003 and exhibited potent antimycobacterial activity, including against multidrug-resistant *M. tuberculosis*, through the inhibition of MraY without significant toxicity in mice [23]. Furthermore, CPZEN-45 developed by structure-activity relationship studies on caprazamycin B [24] exhibited selective growth inhibitory activity against *M. tuberculosis* by inhibiting the phospho-*N*-acetylglucosaminyltransferase WecA [25], which plays a role in mycolyl-arabinogalactan biosynthesis. Therefore, CPZEN-45 is currently being developed as a drug candidate for anti-TB drugs with a new mechanism of action. Caprazamycin B and CPZEN-45 both exhibit in vitro anti-MAC activity [23, 24, 26]; however, their in vivo efficacy has not yet been clarified.

We herein demonstrated the in vivo efficacy of liposidomycins in the silkworm infection model with *M. avium* and *M. intracellulare*. The ED_50_ values of each liposidomycin in the silkworm infection model were lower than their corresponding MIC values in the microdilution method. For example, the MIC value of **1** against *M. avium* was 64 µg ml^−1^, whereas the ED_50_ value was 0.26 µg larva^−1^ g^−1^, indicating that in vivo activity was 250-fold stronger than in vitro activity. Several compounds are reported to show ED_50_ values lower than the MIC values due to enhancement of the in vivo activity by host factors [27, 28] or inhibition of antimicrobial activity by serum albumin or other substances in the culture medium [29]. Therefore, similar causes for the activity of liposidomycins are also possible and need to be clarified in the future.

The MAC therapeutic agent, CAM, was previously reported to only be effective at high doses (50–200 mg g^−1^) in the mouse infection model [30], with similar results being obtained in the silkworm model (Table 3). The ED_50_ values of CAM in the silkworm model with *M. avium* and *M. intracellulare* were 23 and 42 µg larva^−1^ g^−1^, respectively, while the ED_50_ values of **1**–**17** were markedly lower than those of CAM. Further studies are warranted to obtain more information on their in vivo activity in mice. Moreover, **1**–**17** exhibited selective anti-MAC activity without antimicrobial activity against Gram-positive bacteria. These experimental data show the potential of liposidomycins as candidate anti-MAC agents. However, the interpretation of this selective activity cannot be resolved by MraY inhibitory activity alone. Therefore, the possibility of other factors, such as targets other than MraY or selective transporters, is suggested.

In conclusion, we used an in vivo mimetic silkworm infection model to screen for anti-MAC agents and discovered liposidomycins with therapeutic effects. Although the target molecule of liposidomycins was previously reported to be MraY, it remains unclear whether this prominent in vivo anti-MAC activity is due to the direct inhibition of MraY or another mechanism of action. Therefore, future research on the anti-MAC activity of not only liposidomycins, but also other MraY inhibitors is needed to clarify this issue.

### Supplementary information

Supplementary Information
