# Supplementary material for: New liposidomycin congeners produced by Streptomyces sp. TMPU-20A065, anti-Mycobacterium avium complex agents with therapeutic efficacy in a silkworm infection model
Source: J Antibiot (Tokyo). 2024 May 8;77(7):412–21. doi: 10.1038/s41429-024-00724-4 (PMC11208132; doi:10.1038/s41429-024-00724-4)
Supplement: Supplementary file 1 — Supplementary Information [file 41429_2024_724_MOESM1_ESM.docx]

**Supplementary Information**

**New liposidomycin congeners produced by *Streptomyces* sp. TMPU- 20A065, anti-*Mycobacterium avium* complex agents with therapeutic efficacy in silkworm infection model**

Akiho Yagi, Mayu Fujiwara, Mayu Sato, Yuzu Abe, and Ryuji Uchida

Scheme 1 Fermentation and isolation procedure of **1**–**17** ………………………………5

Figure S1 HPLC profile of extract containing **1** to **4**……………………………………6

Figure S2 HPLC profile of extract containing **5** to **17**……………………………………6

Figure S3 NOE correlations and ^1^H-^1^H coupling constants of new liposidomycins (a: **1**, b: **2**, and c: **4**)……………………………………………………………………………7

Figure S4 ^1^H NMR spectrum of **1** (600 MHz, CD_3_OD)………………………………8

Figure S5 ^13^C NMR spectrum of **1** (150 MHz, CD_3_OD)……………………………8

Figure S6 HMQC spectrum of **1** (600 MHz, CD_3_OD)………….…………………….9

Figure S7 COSY spectrum of **1** (600 MHz, CD_3_OD)……………………………….9

Figure S8 HMBC spectrum of **1** (600 MHz, CD_3_OD)………………………………10

Figure S9 ROESY spectrum of **1** (600 MHz, CD_3_OD)……………………………….10

Figure S10 ^1^H NMR spectrum of **2** (600 MHz, CD_3_OD)………………………………11

Figure S11 ^13^C NMR spectrum of **2** (150 MHz, CD_3_OD)……………………………11

Figure S12 HMQC spectrum of **2** (600 MHz, CD_3_OD)………………………………12

Figure S13 COSY spectrum of **2** (600 MHz, CD_3_OD)………………………………12

Figure S14 HMBC spectrum of **2** (600 MHz, CDCl_3_)………….…...………………13

Figure S15 ROESY spectrum of **2** (600 MHz, CD_3_OD)…………………………….13

Figure S16 ^1^H NMR spectrum of **4** (600 MHz, CD_3_OD)……………………………14

Figure S17 ^13^C NMR spectrum of **4** (150 MHz, CD_3_OD)……………………………14

Figure S18 HMQC spectrum of **4** (600 MHz, CD_3_OD)……………………………15

Figure S19 COSY spectrum of **4** (600 MHz, CD_3_OD)……………………………15

Figure S20 HMBC spectrum of **4** (600 MHz, CD_3_OD)……………………………16

Figure S21 ROESY spectrum of **4** (600 MHz, CD_3_OD)……………………………16

Figure S22 ^1^H NMR spectrum of **3** (600 MHz, CD_3_OD)………………………………17

Figure S23 HRESIMS spectrum of **3**…………………………………………………17

Figure S24 UV spectrum of **3**…………………………………………………………18

Figure S25 ^1^H NMR spectrum of **5** (600 MHz, CD_3_OD)………………………………18

Figure S26 HRESIMS spectrum of **5**…………………………………………………19

Figure S27 UV spectrum of **5**…………………………………………………………19

Figure S28 ^1^H NMR spectrum of **6** (600 MHz, CD_3_OD)………………………………20

Figure S29 HRESIMS spectrum of **6**…………………………………………………20

Figure S30 UV spectrum of **6**…………………………………………………………21

Figure S31 ^1^H NMR spectrum of **7** (600 MHz, CD_3_OD)………………………………21

Figure S32 HRESIMS spectrum of **7**…………………………………………………22

Figure S33 UV spectrum of **7**…………………………………………………………22

Figure S34 ^1^H NMR spectrum of **8** (600 MHz, CD_3_OD)………………………………23

Figure S35 HRESIMS spectrum of **8**…………………………………………………23

Figure S36 UV spectrum of **8**…………………………………………………………24

Figure S37 ^1^H NMR spectrum of **9** (600 MHz, CD_3_OD)………………………………24

Figure S38 HRESIMS spectrum of **9**…………………………………………………25

Figure S39 UV spectrum of **9**…………………………………………………………25

Figure S40 ^1^H NMR spectrum of **10** (600 MHz, CD_3_OD)………………………………26

Figure S41 HRESIMS spectrum of **10**…………………………………………………26

Figure S42 UV spectrum of **10**…………………………………………………………27

Figure S43 ^1^H NMR spectrum of **11** (600 MHz, CD_3_OD)………………………………27

Figure S44 HRESIMS spectrum of **11**…………………………………………………28

Figure S45 UV spectrum of **11**…………………………………………………………28

Figure S46 ^1^H NMR spectrum of **12** (600 MHz, CD_3_OD)………………………………29

Figure S47 HRESIMS spectrum of **12**…………………………………………………29

Figure S48 UV spectrum of **12**…………………………………………………………30

Figure S49 ^1^H NMR spectrum of **13** (600 MHz, CD_3_OD)………………………………30

Figure S50 HRESIMS spectrum of **13**…………………………………………………31

Figure S51 UV spectrum of **13**…………………………………………………………31

Figure S52 ^1^H NMR spectrum of **14** (600 MHz, CD_3_OD)………………………………32

Figure S53 HRESIMS spectrum of **14**…………………………………………………32

Figure S54 UV spectrum of **14**…………………………………………………………33

Figure S55 ^1^H NMR spectrum of **15** (600 MHz, CD_3_OD)………………………………33

Figure S56 HRESIMS spectrum of **15**…………………………………………………34

Figure S57 UV spectrum of **15**…………………………………………………………34

Figure S58 ^1^H NMR spectrum of **16** (600 MHz, CD_3_OD)………………………………35

Figure S59 HRESIMS spectrum of **16**…………………………………………………35

Figure S60 UV spectrum of **16**…………………………………………………………36

Figure S61 ^1^H NMR spectrum of **17** (600 MHz, CD_3_OD)………………………………36

Figure S62 HRESIMS spectrum of **17**…………………………………………………37

Figure S63 UV spectrum of **17**…………………………………………………………37

**Scheme 1 Fermentation and isolation procedure of 1–17**

**Figure S1 HPLC profile of extract containing 1 to 4**

The extract containing **1** to **4** was purified by preparative HPLC (column, PEGASIL ODS SP100 (Senshu scientific Co., Tokyo, Japan, i.d. 4.6 × 250 mm); mobile phase, 37% CH_3_CN, 0.05% TFA isocratic; detection, UV at 210 nm; flow rate, 1.0 ml min^-1^)

**Figure S2 HPLC profile of extract containing 5 to 17**

The extract containing **5** to **17** was purified by preparative HPLC (column, PEGASIL ODS SP100 (Senshu scientific Co., Tokyo, Japan, i.d. 4.6 × 250 mm); mobile phase, 35–60% CH_3_CN, 0.05% TFA gradient (0–50 min); detection, UV at 210 nm; flow rate, 1.0 ml min^-1^)

**Figure S3 NOE correlations and ^1^H-^1^H coupling constants of new liposidomycins (a: 1, b: 2, and c: 4)**

**
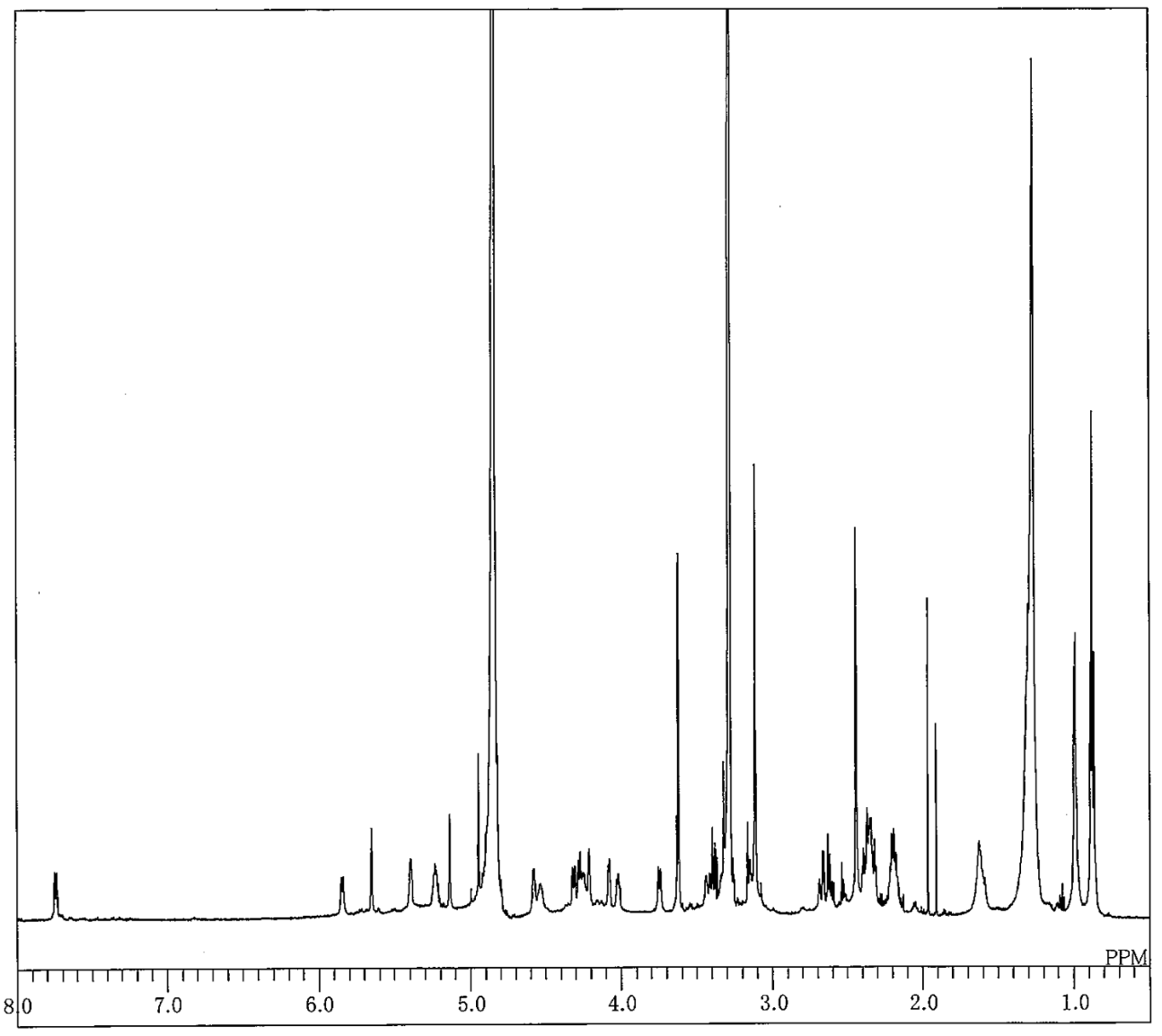
**

**Figure S4 ^1^H NMR spectrum of 1 (600 MHz, CD_3_OD)**

**
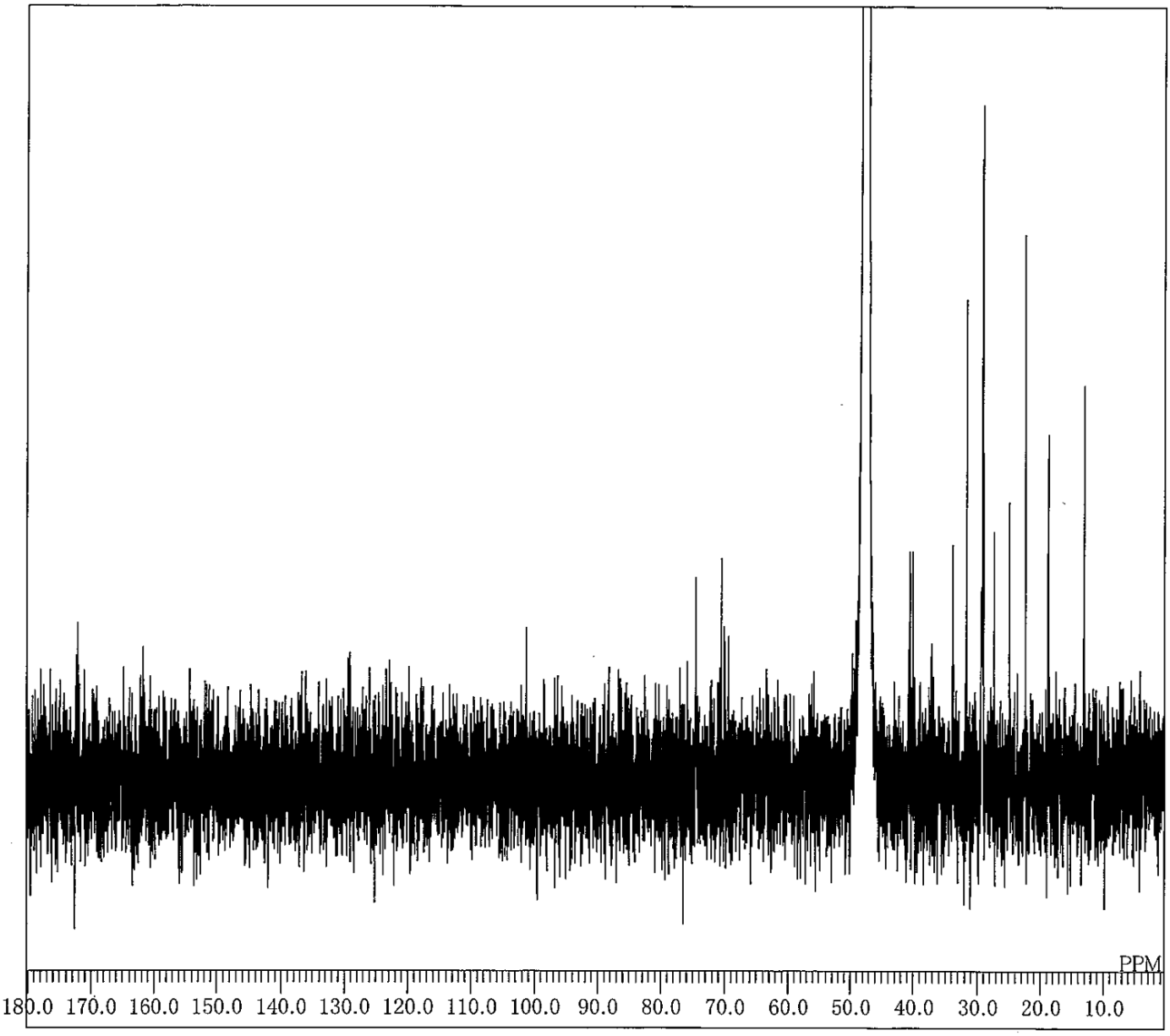
**

**Figure S5 ^13^C NMR spectrum of 1 (150 MHz, CD_3_OD)**

**
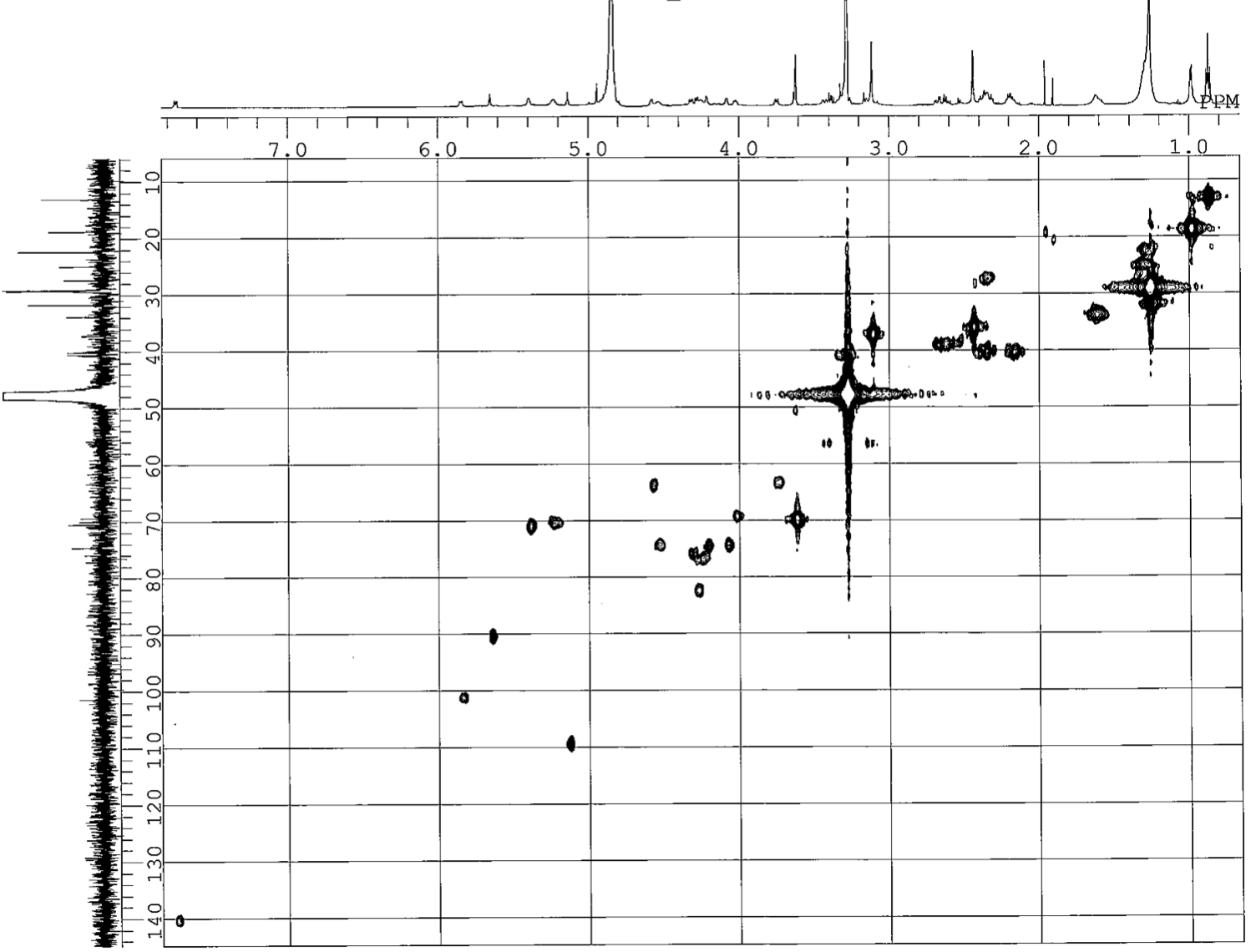
**

**Figure S6 HMQC spectrum of 1 (600 MHz, CD_3_OD)**

**
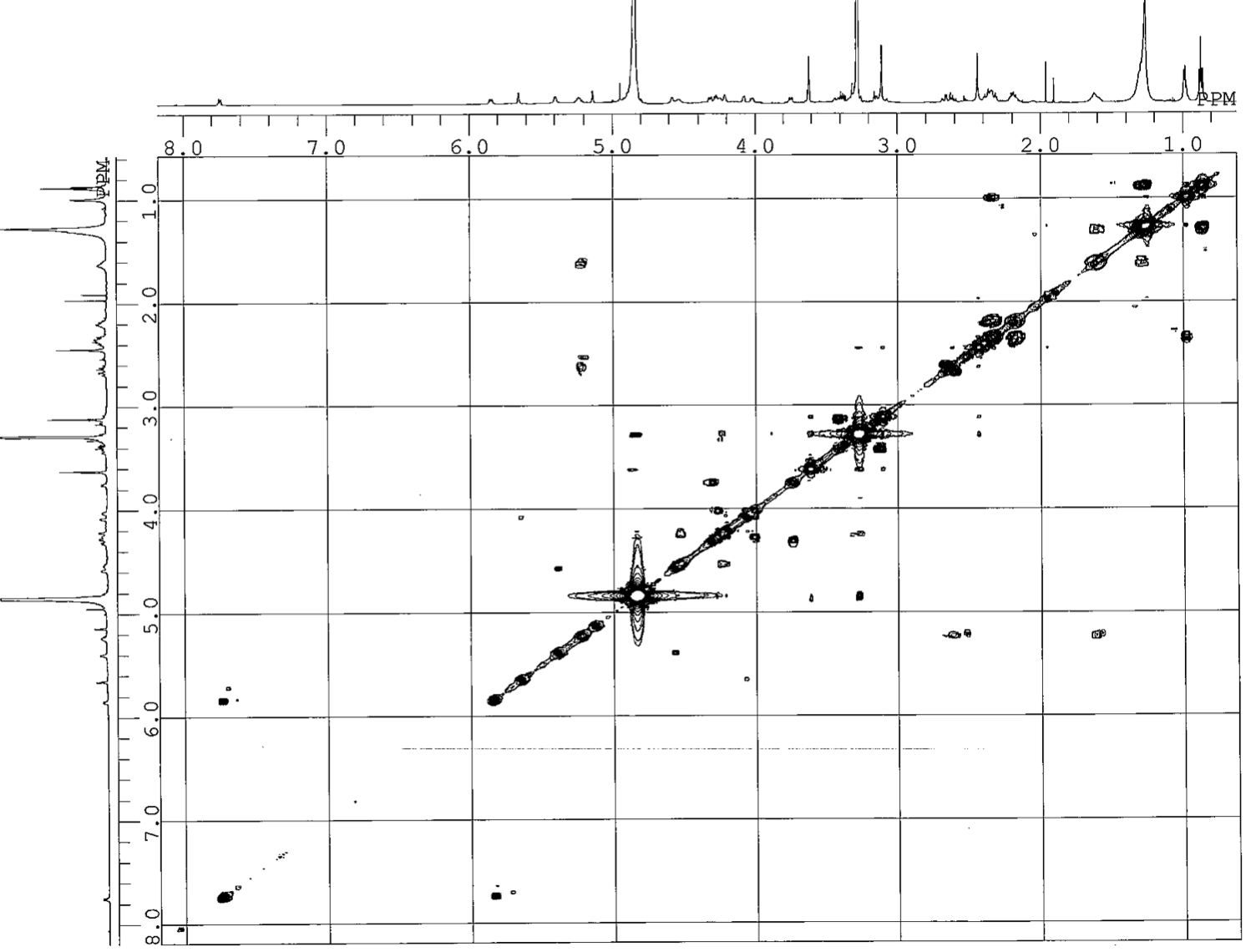
**

**Figure S7 COSY spectrum of 1 (600 MHz, CDCl_3_)**

**
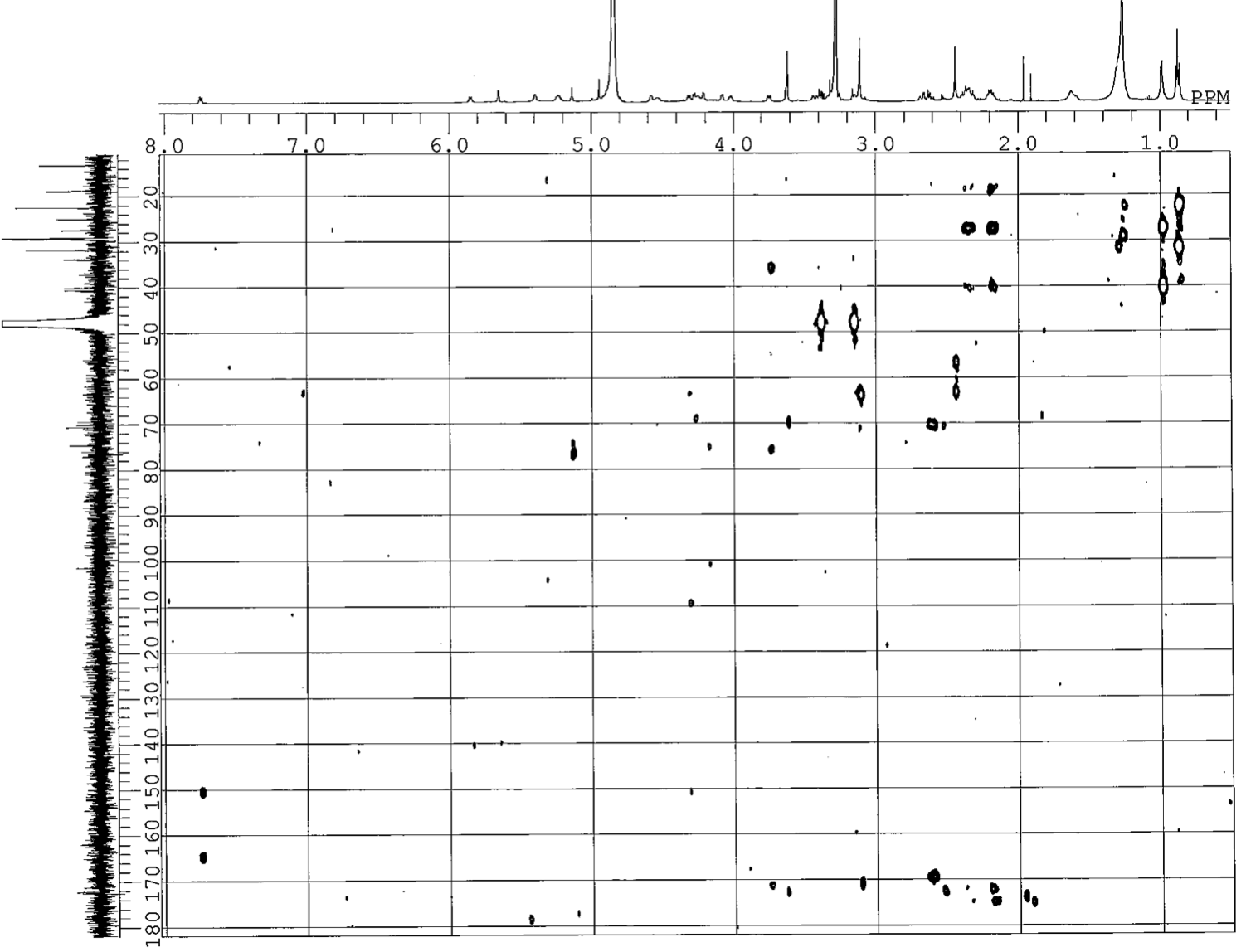
**

**Figure S8 HMBC spectrum of 1 (600 MHz, CD_3_OD)**

**
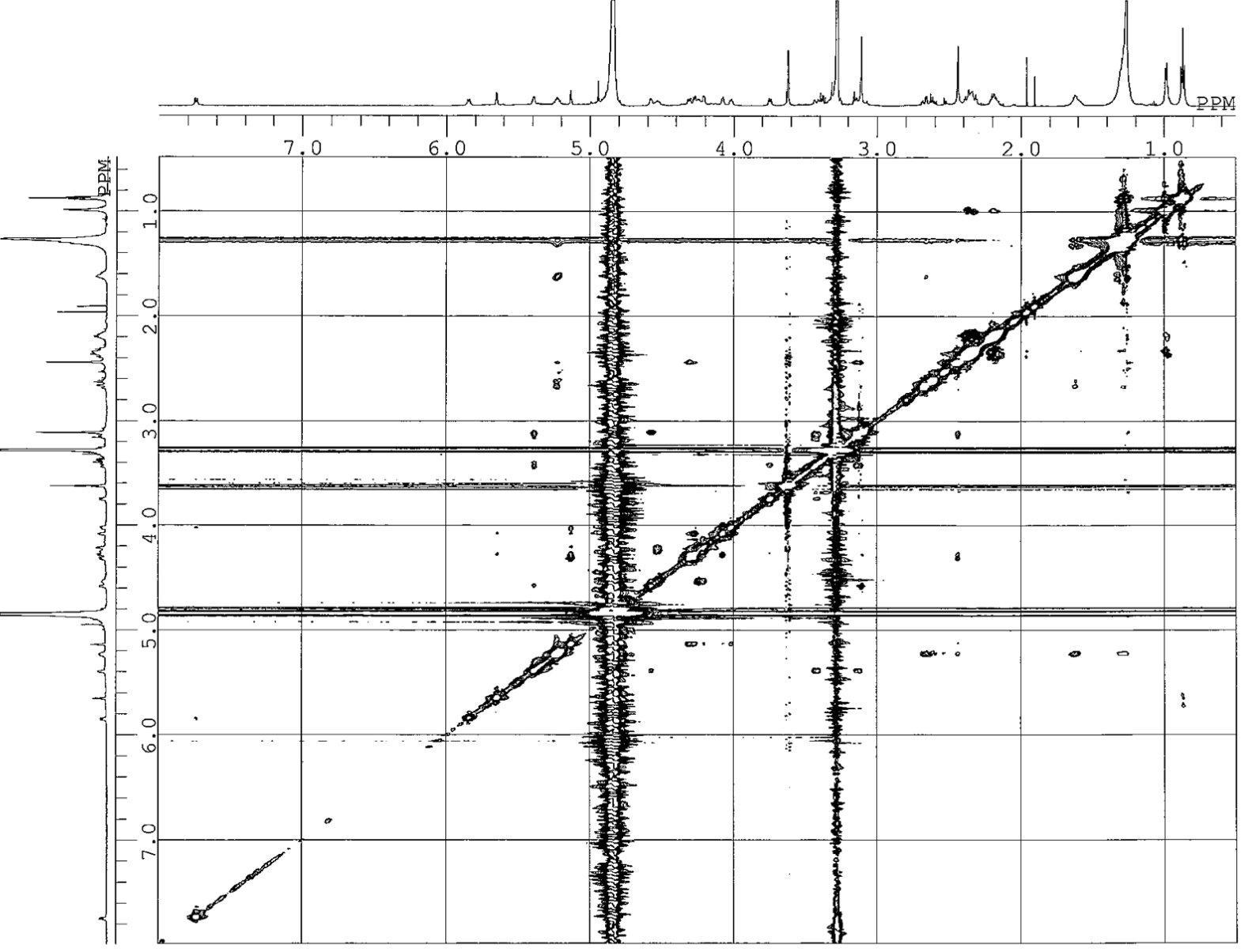
**

**Figure S9 ROESY spectrum of 1 (600 MHz, CD_3_OD)**

**
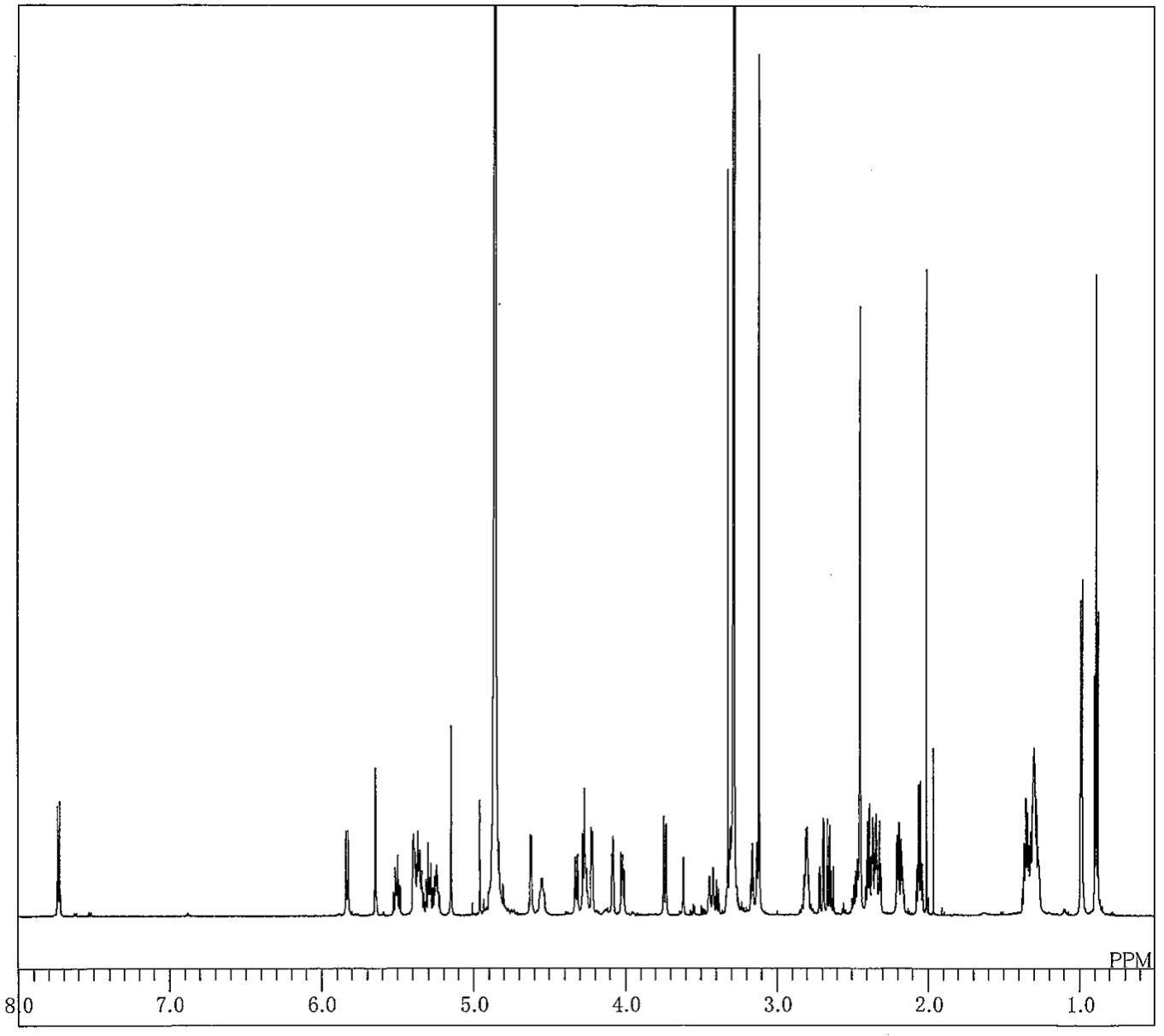
**

**Figure S10 ^1^H NMR spectrum of 2 (600 MHz, CD_3_OD)**

**
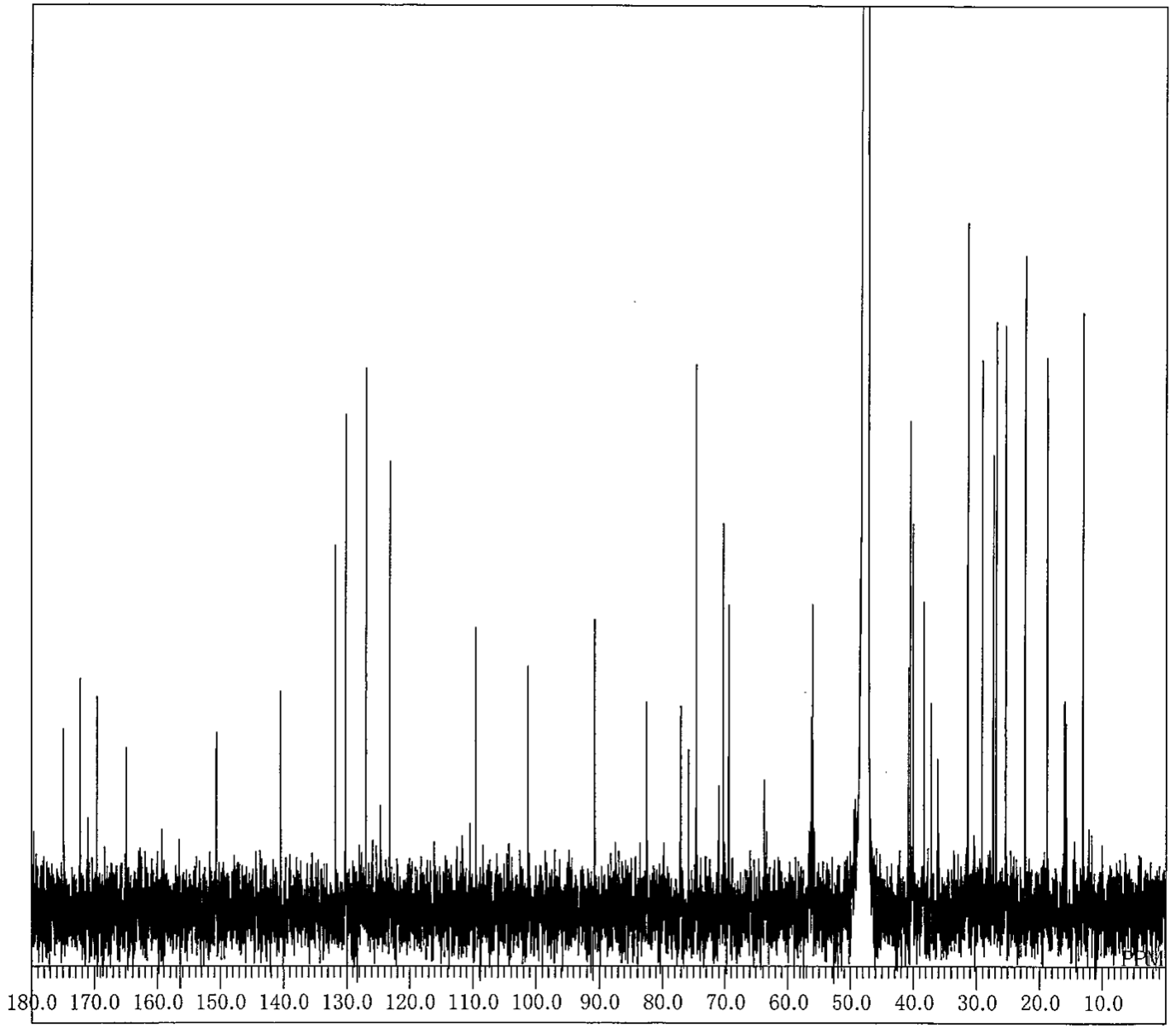
**

**Figure S11 ^13^C NMR spectrum of 2 (150 MHz, CD_3_OD)**


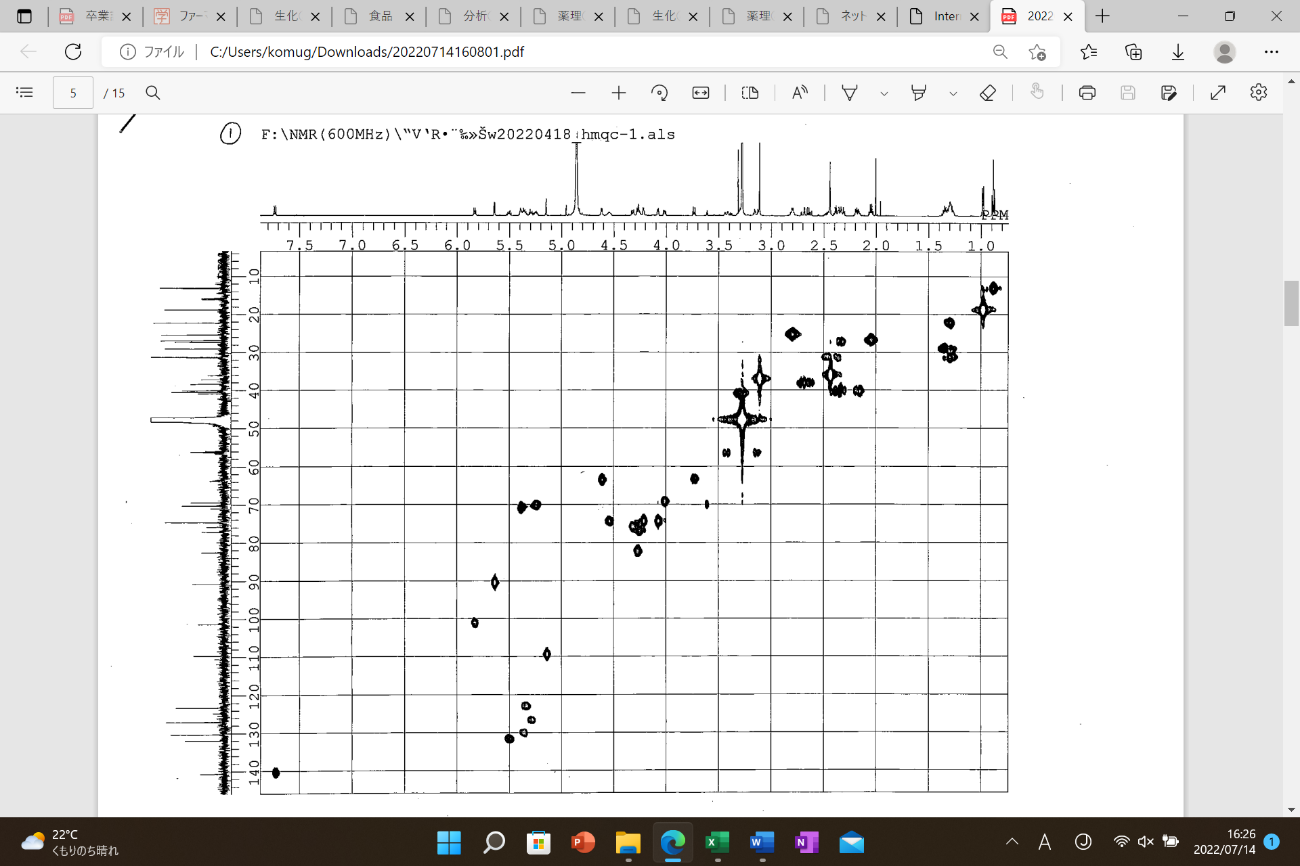


**Figure S12 HMQC spectrum of 2 (600 MHz, CD_3_OD)**

**
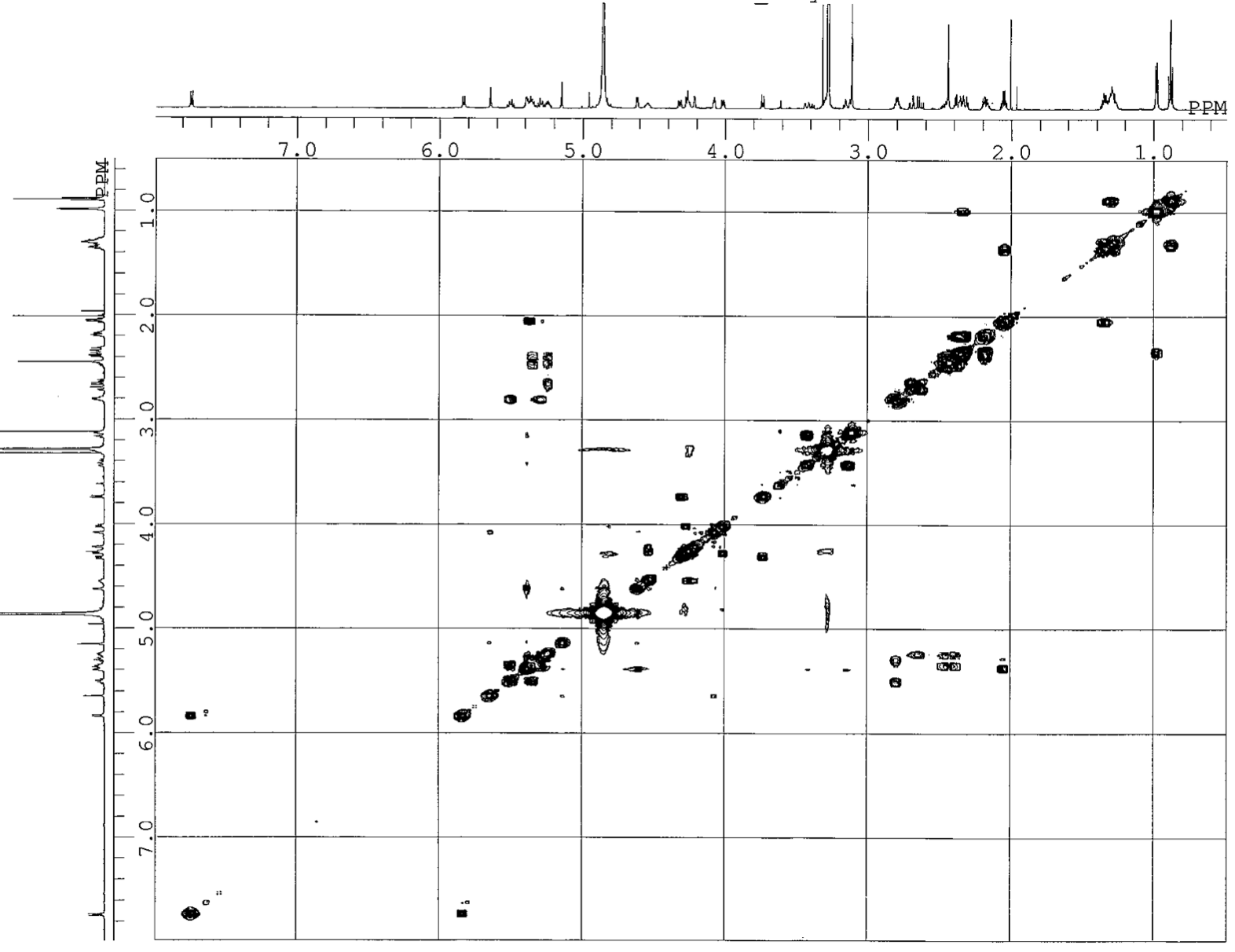
**

**Figure S13 COSY spectrum of 2 (600 MHz, CD_3_OD)**


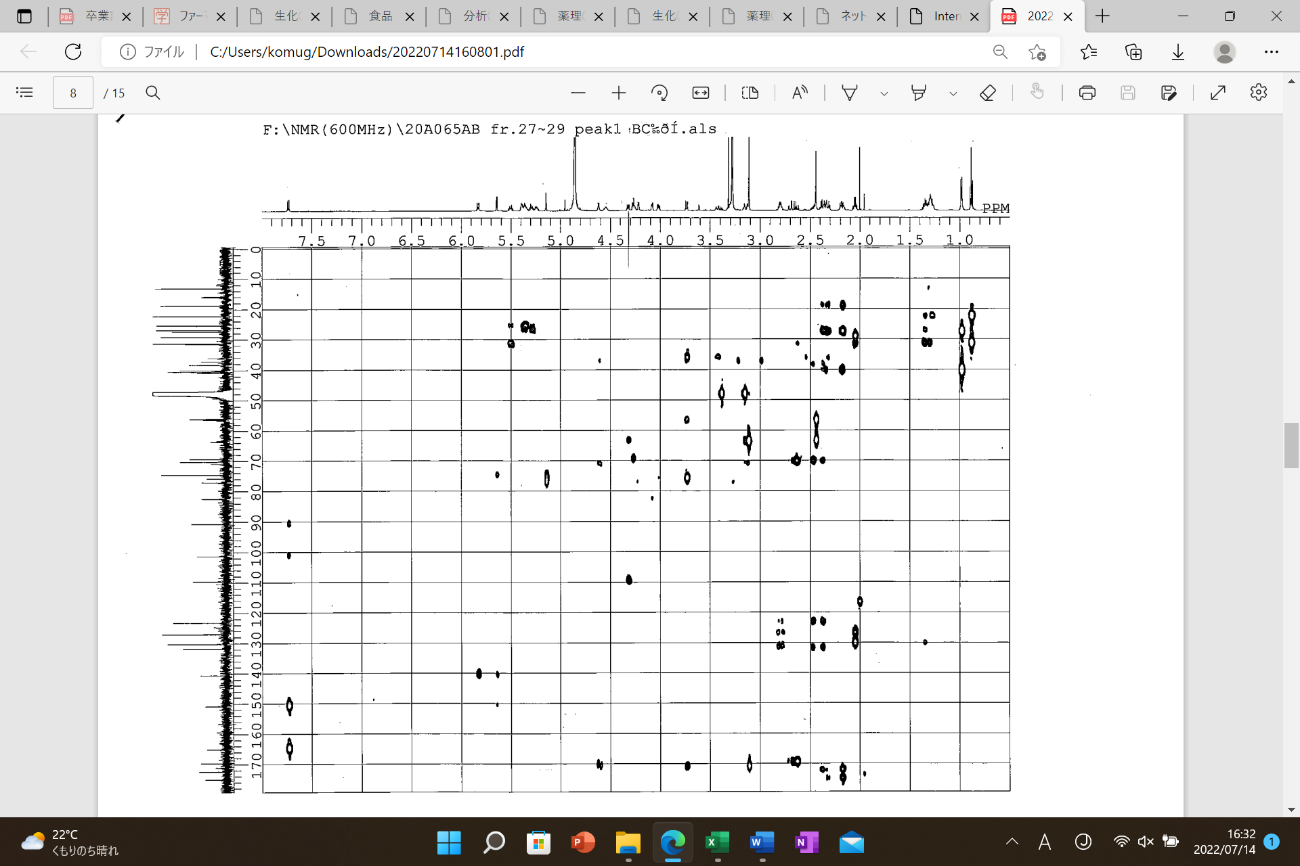


**Figure S14 HMBC spectrum of 4 (600 MHz, 150 MHz, CD_3_OD)**

**
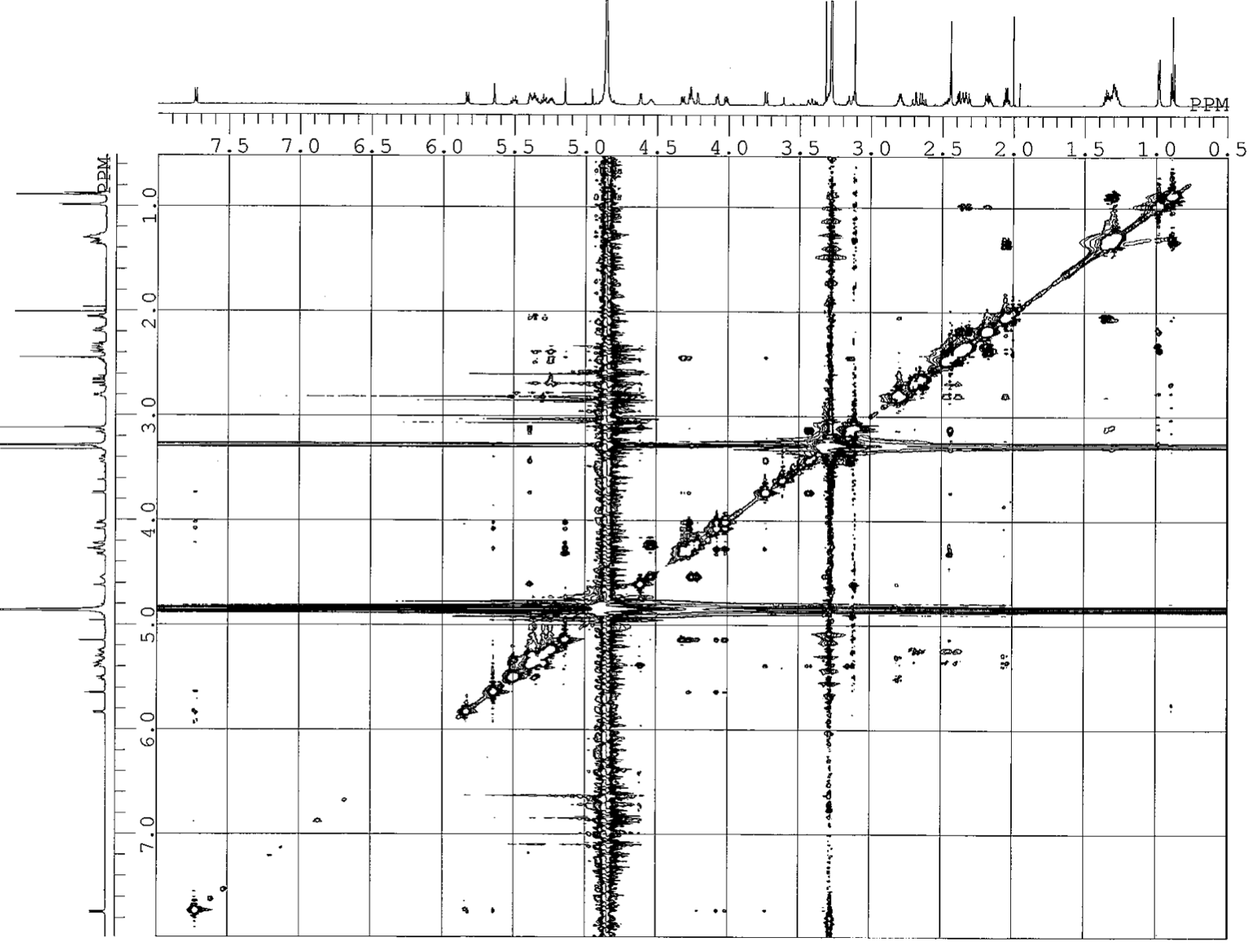
**

**Figure S15 ROESY spectrum of 2 (600 MHz, CD_3_OD)**

**
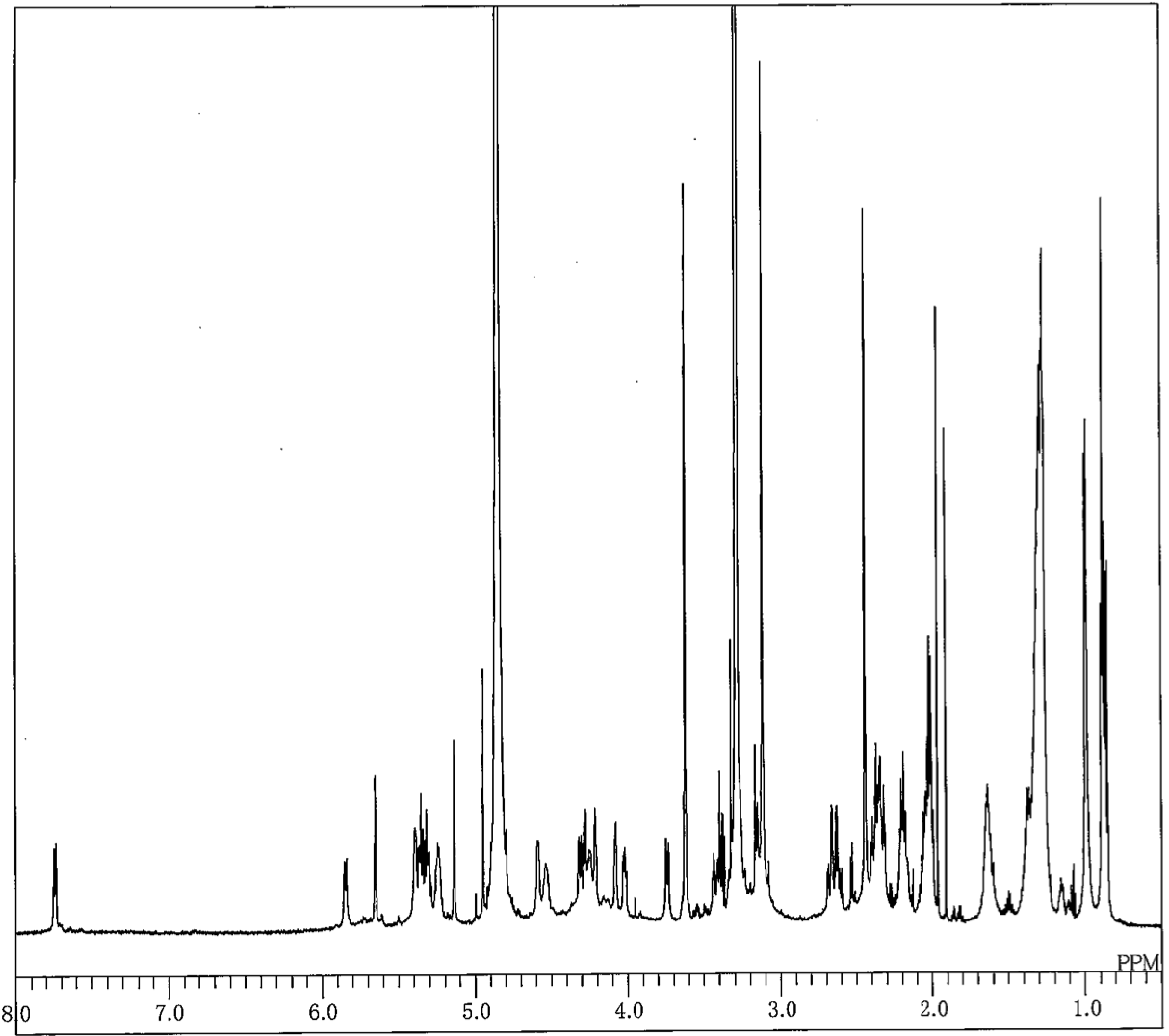
**

**Figure S16 ^1^H NMR spectrum of 4 (600 MHz, CD_3_OD)**

**
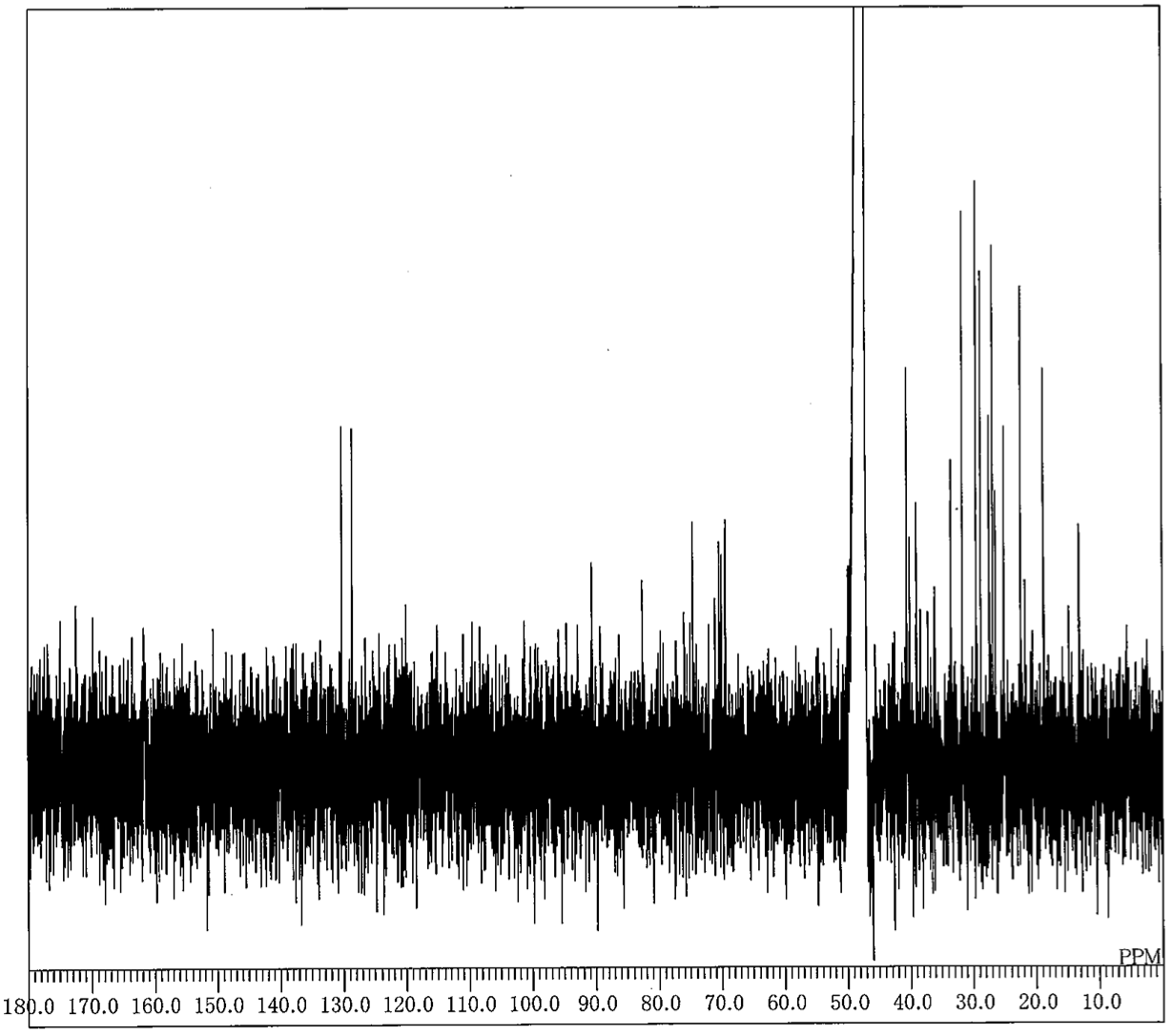
**

**Figure S17 ^13^C NMR spectrum of 4 (150 MHz, CD_3_OD)**

**
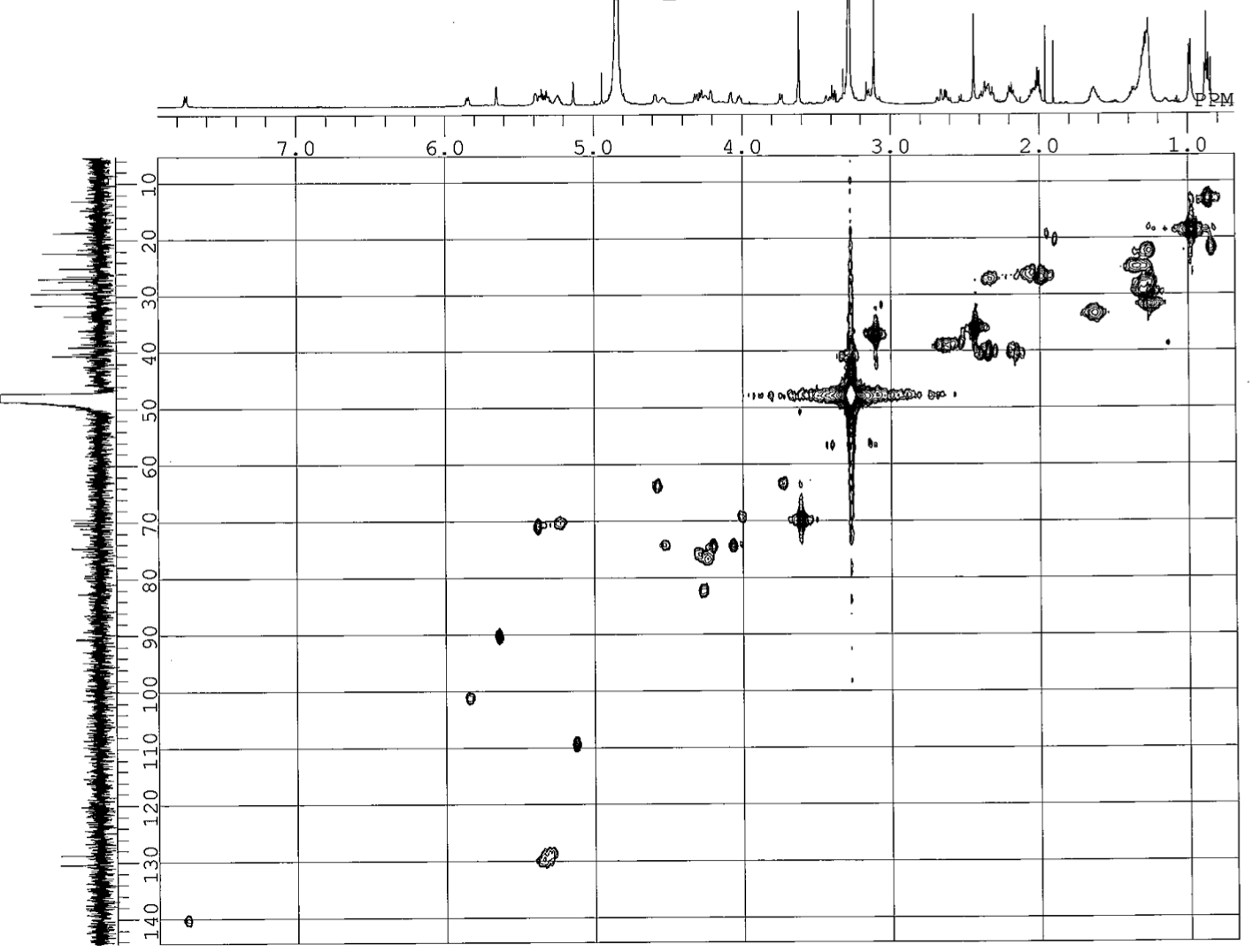
**

**Figure S18 HMQC spectrum of 4 (600 MHz, CD_3_OD)**

**
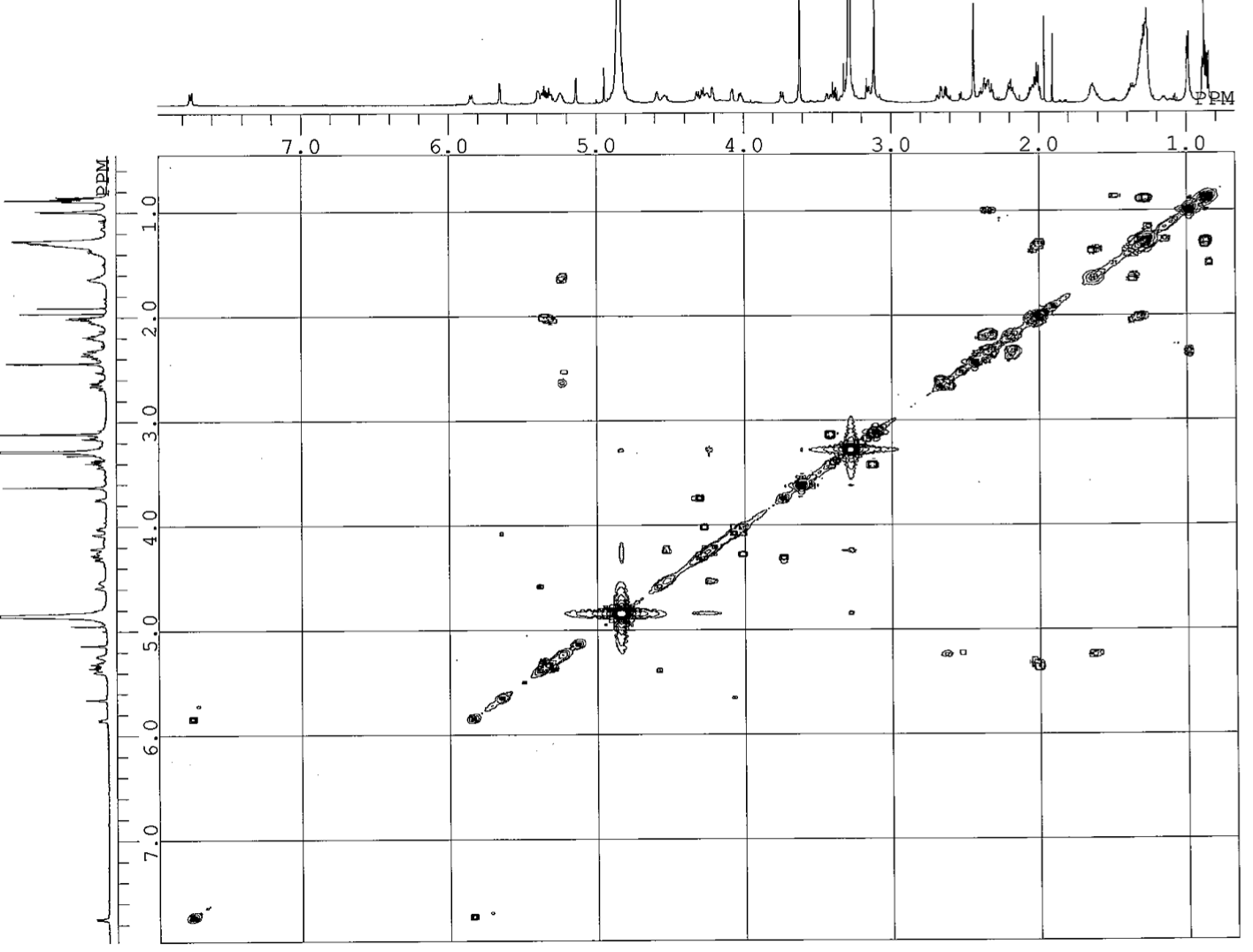
**

**Figure S19 COSY spectrum of 4 (600 MHz, CD_3_OD)**

**
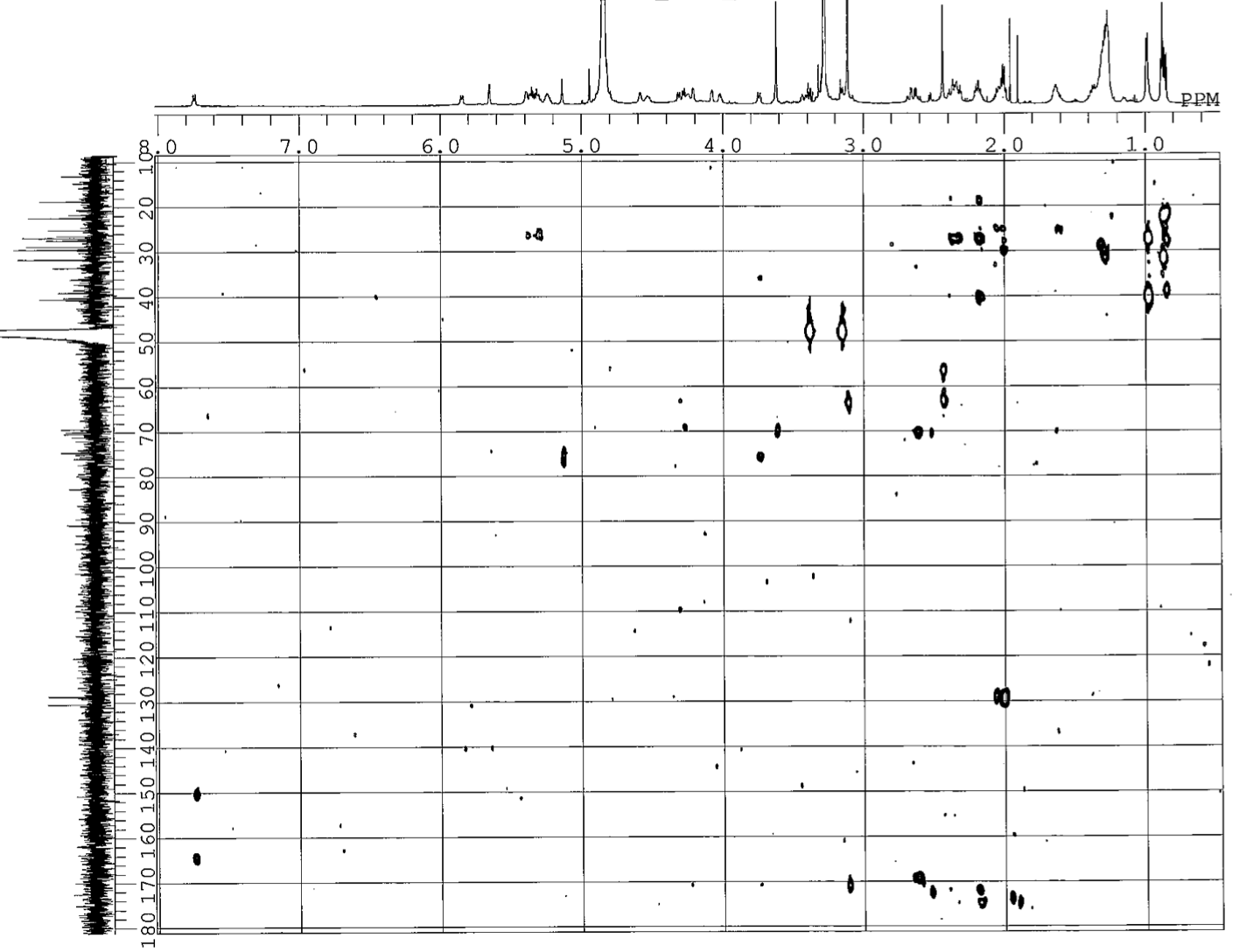
**

**Figure S20 HMBC spectrum of 4 (600 MHz, CD_3_OD)**

**
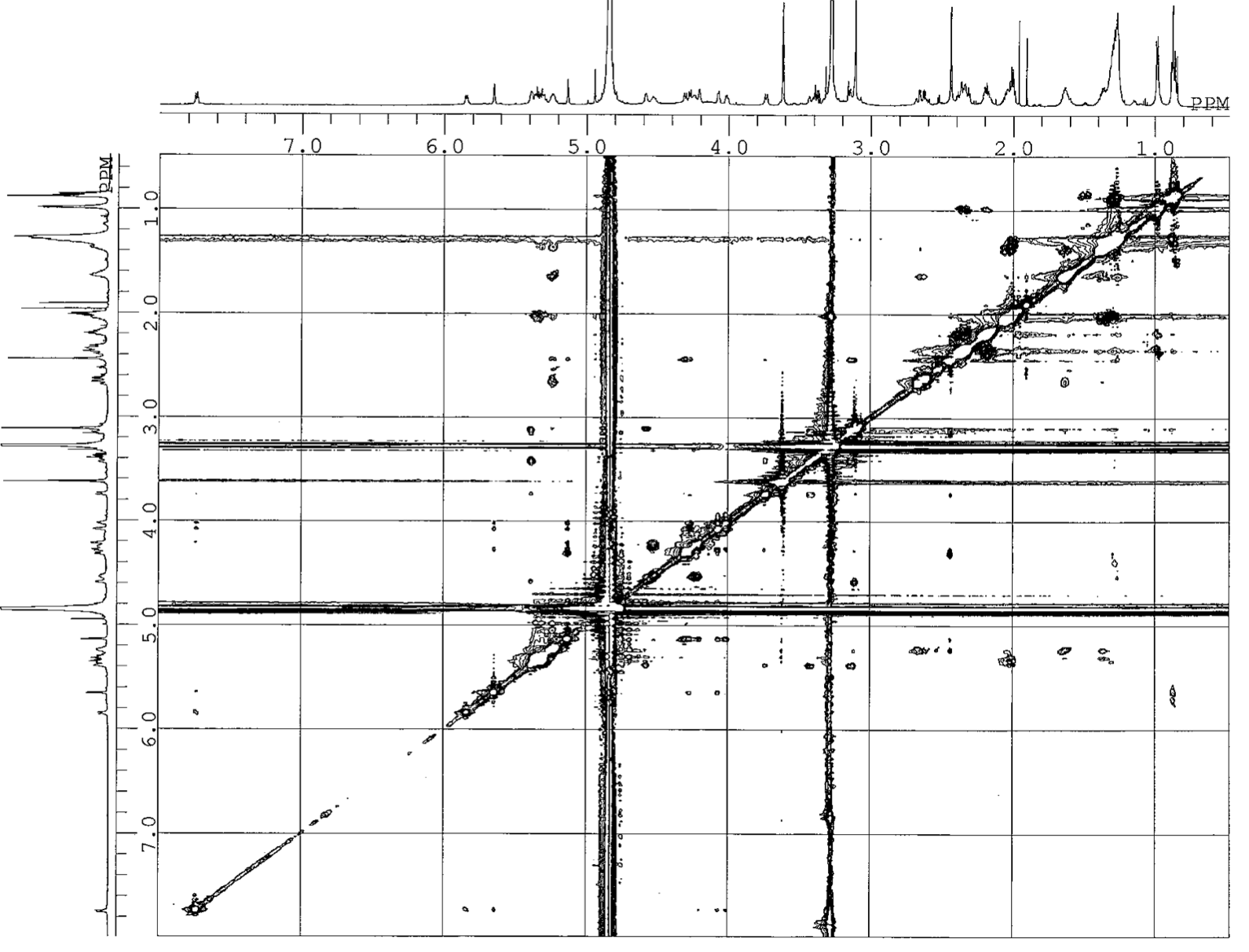
**

**Figure S21 ROESY spectrum of 4 (600 MHz, CD_3_OD).**

**
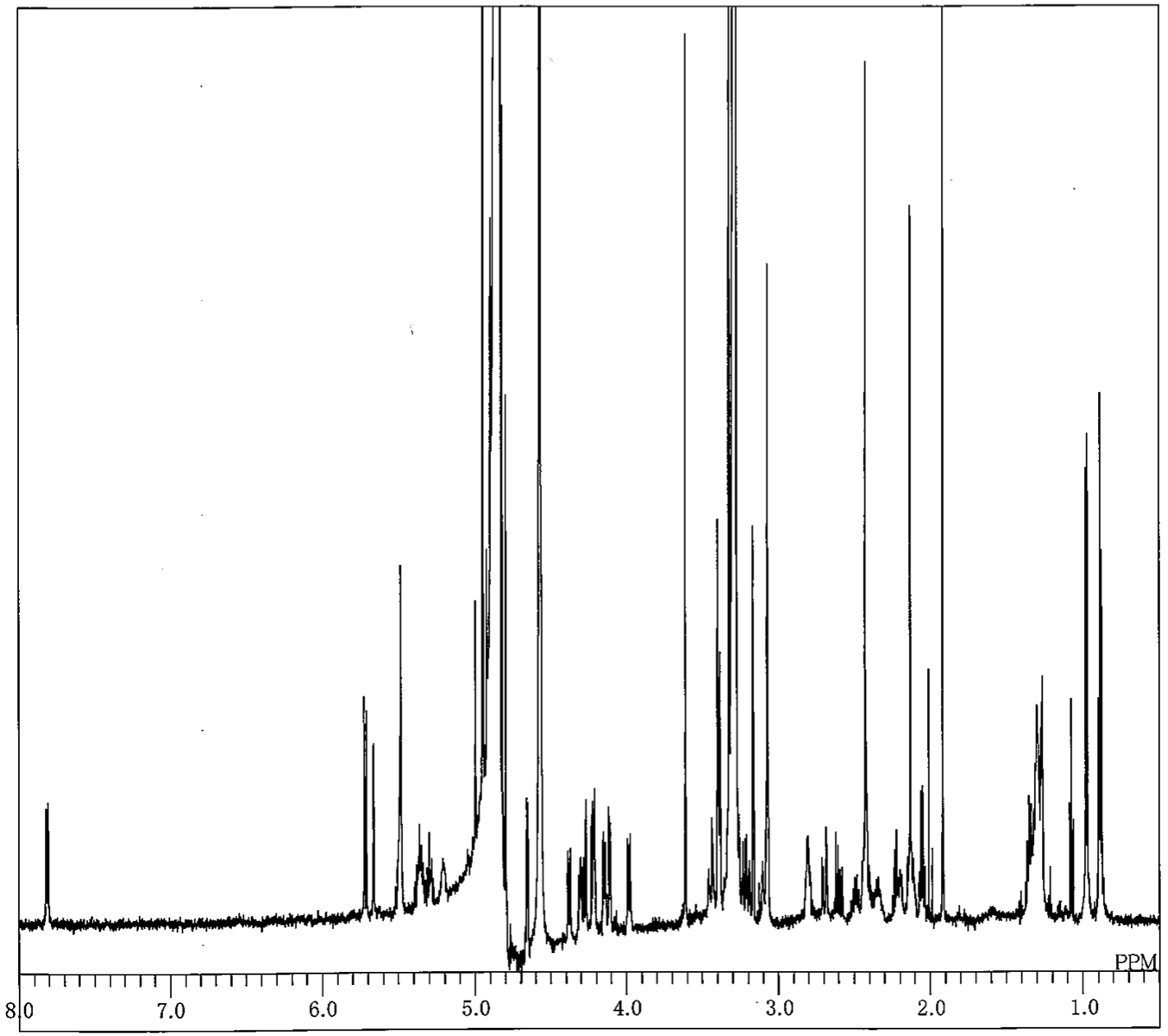
**

**Figure S22 ^1^H NMR spectrum of 3 (600 MHz, CD_3_OD)**

**
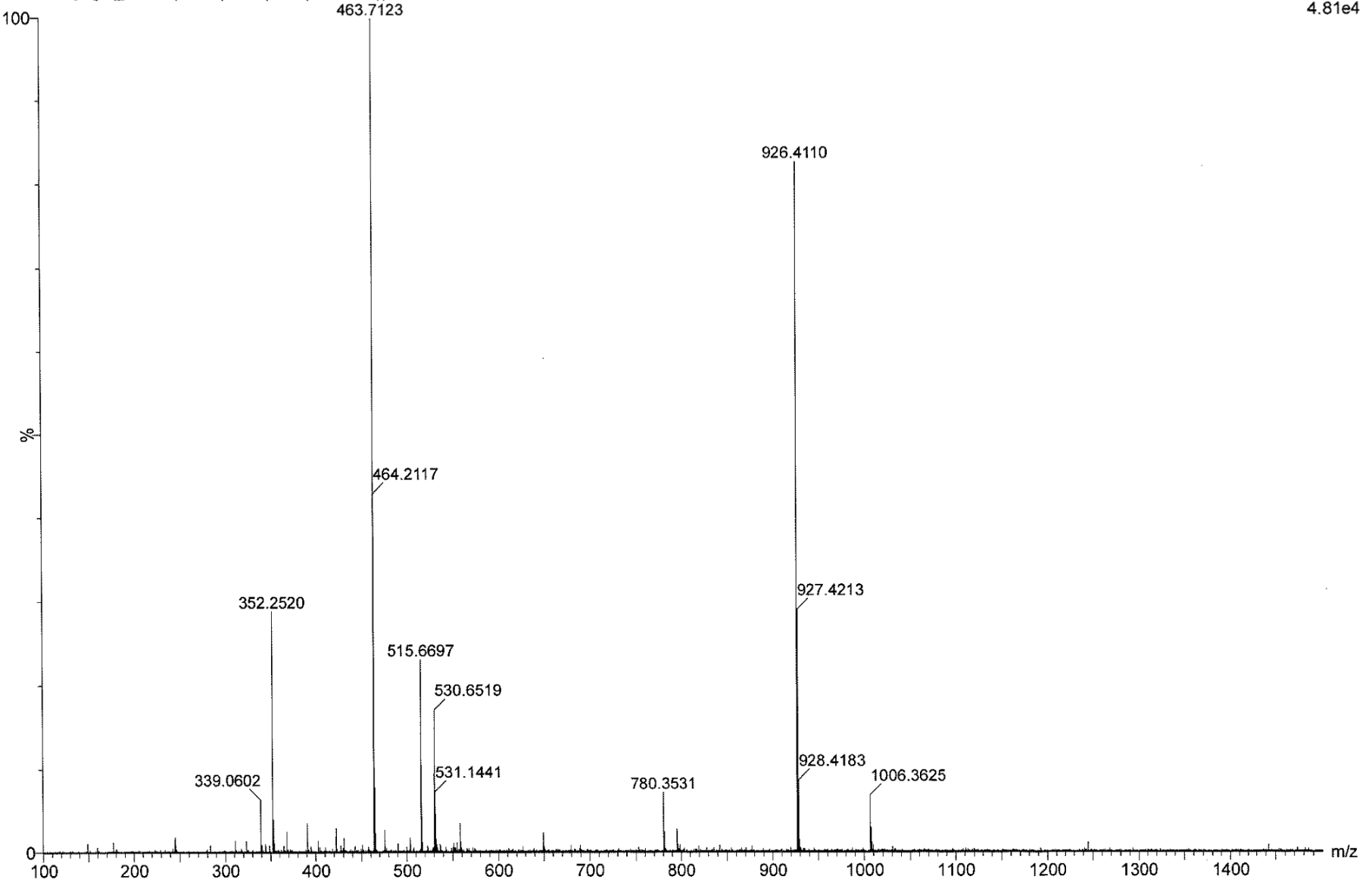
**

[M + H]^+^

**Figure S23 HRESIMS spectrum of 3**

**Figure S24 UV spectrum of 3**

**
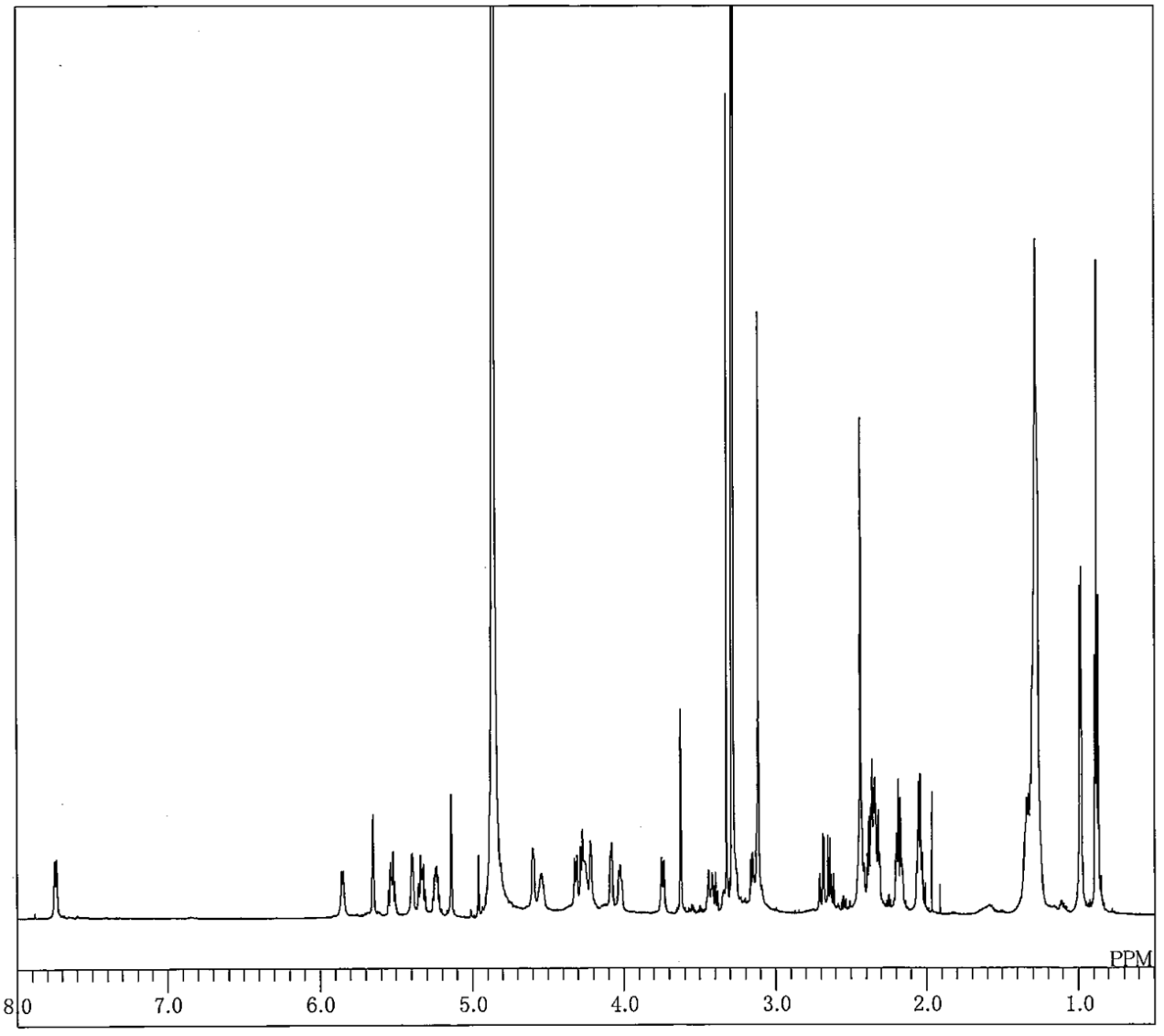
**

**Figure S25 ^1^H NMR spectrum of 5 (600 MHz, CD_3_OD)**

**
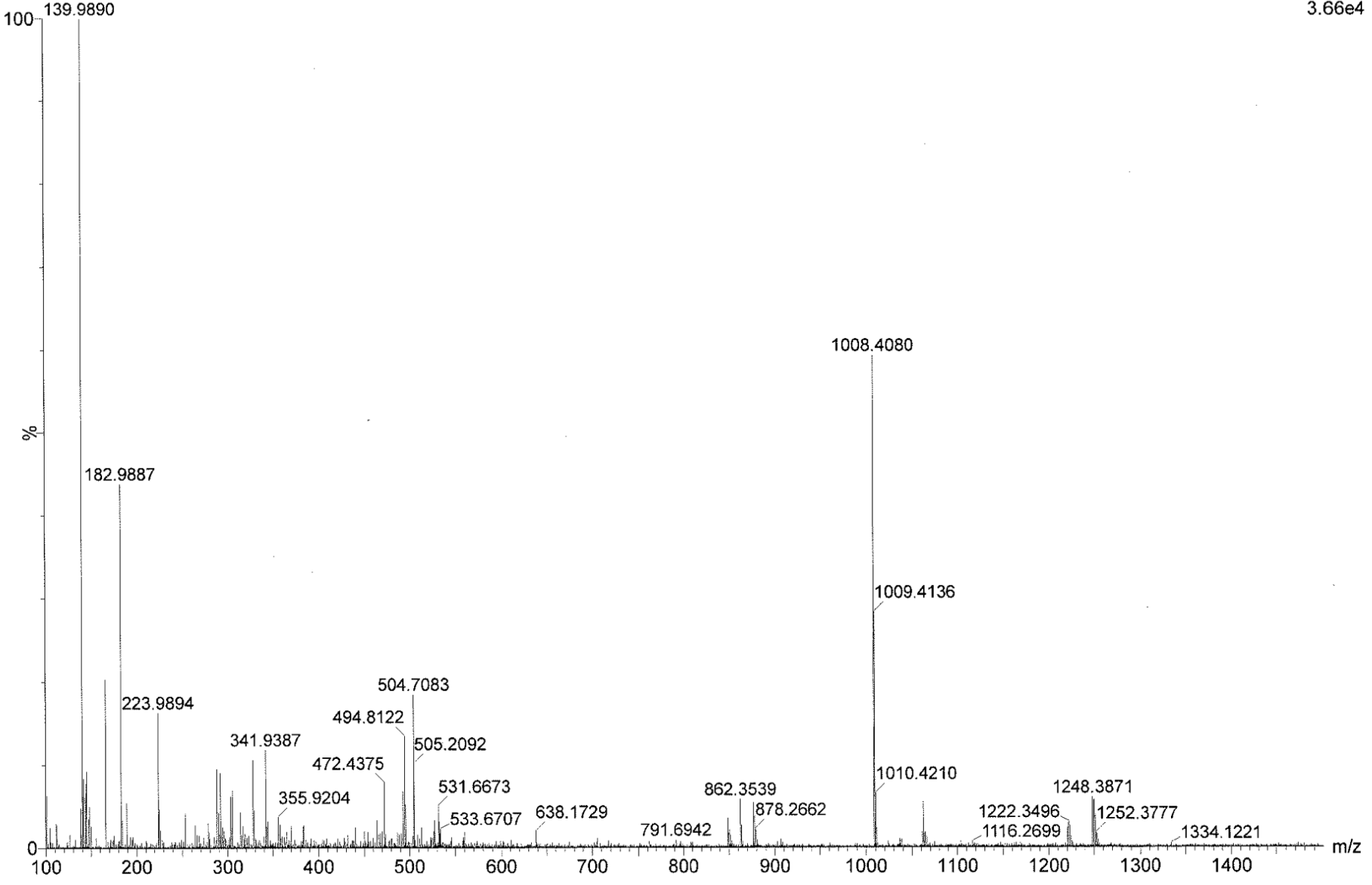
**

[M + H]^+^

**Figure S26 HRESIMS spectrum of 5**

**Figure S27 UV spectrum of 5**

**
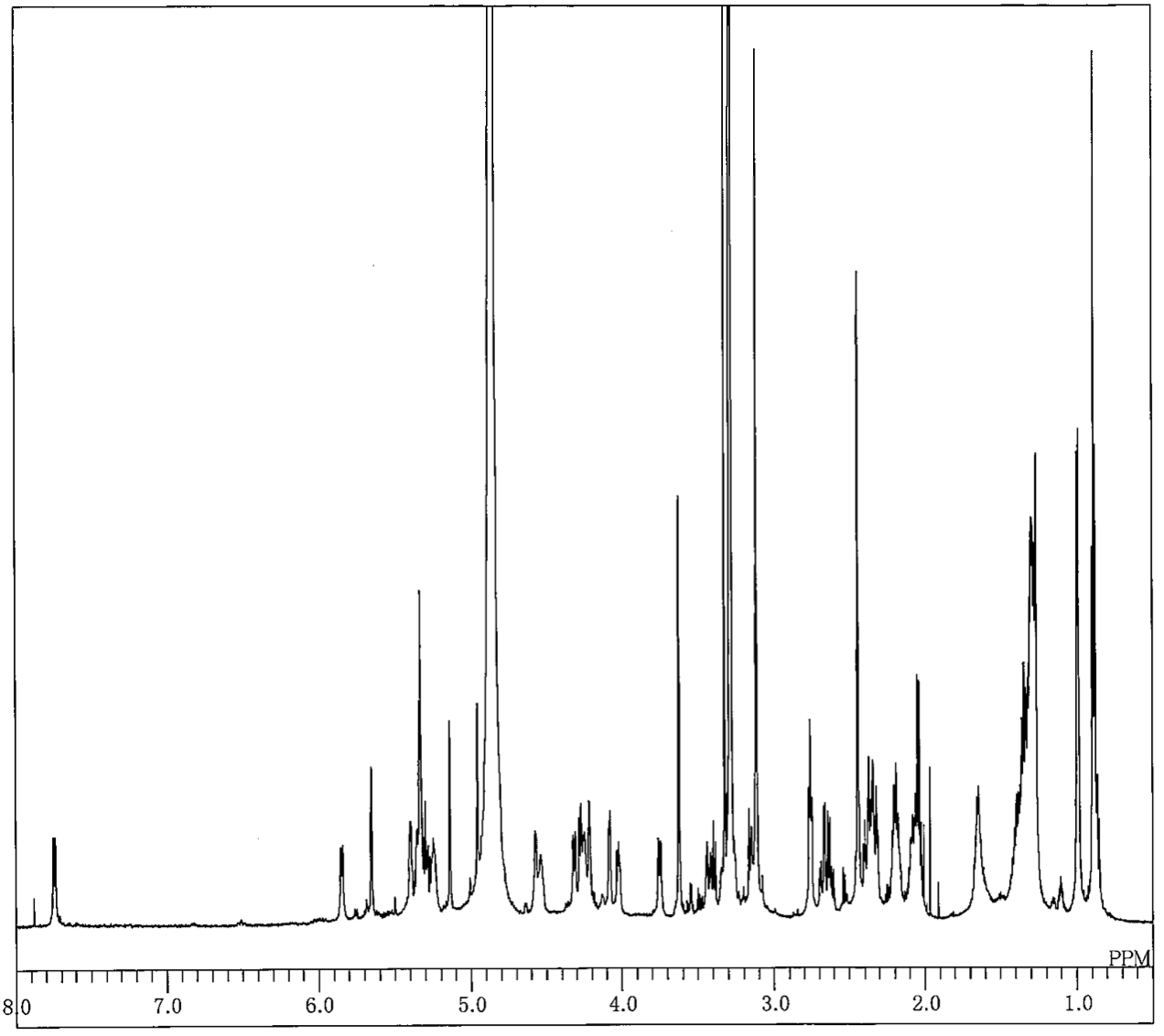
**

Figure S28 ^1^H NMR spectrum of **6** (600 MHz, CD_3_OD)


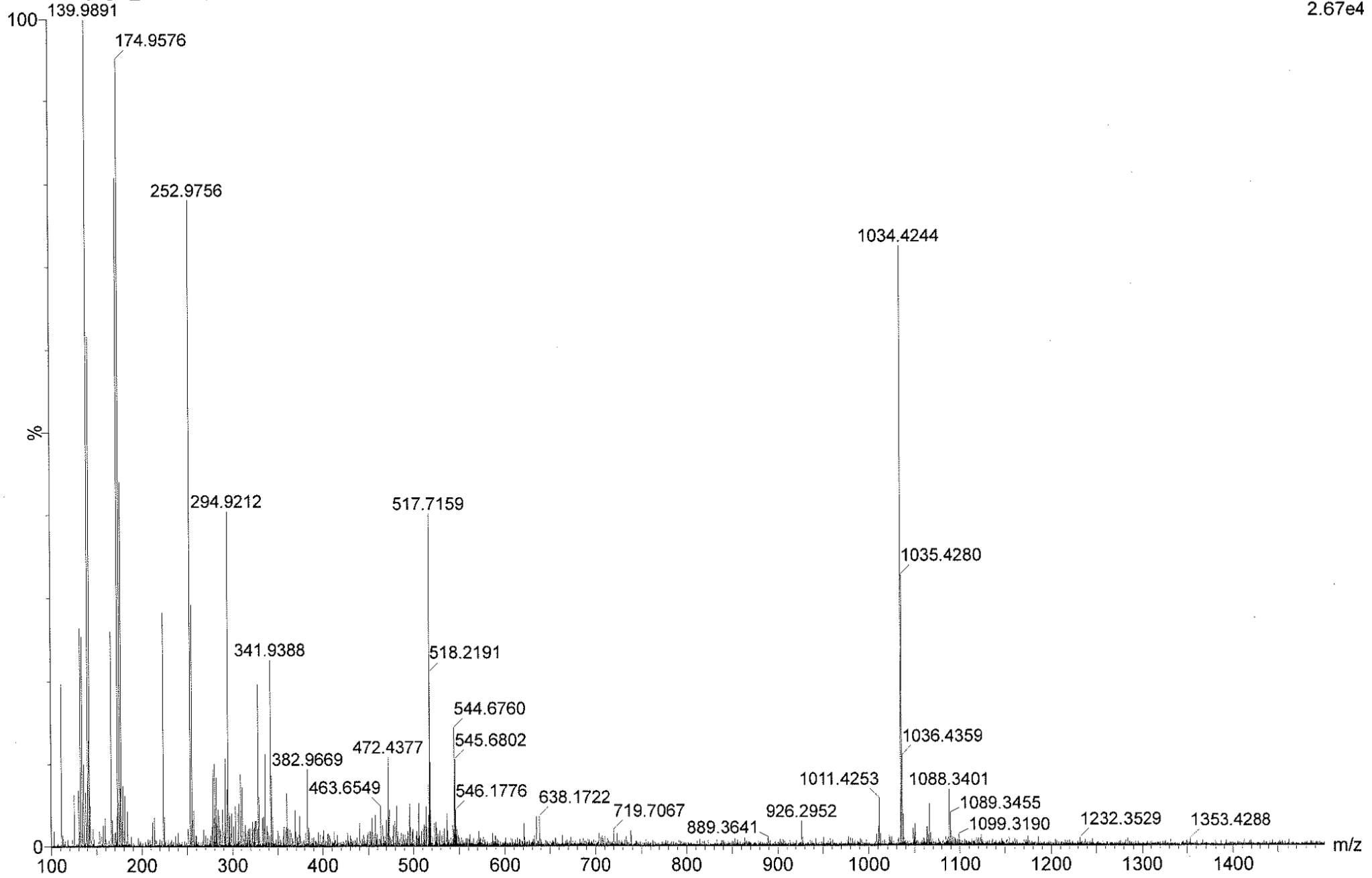


[M + H]^+^

Figure S29 HRESIMS spectrum of **6**

Figure S30 UV spectrum of **6**


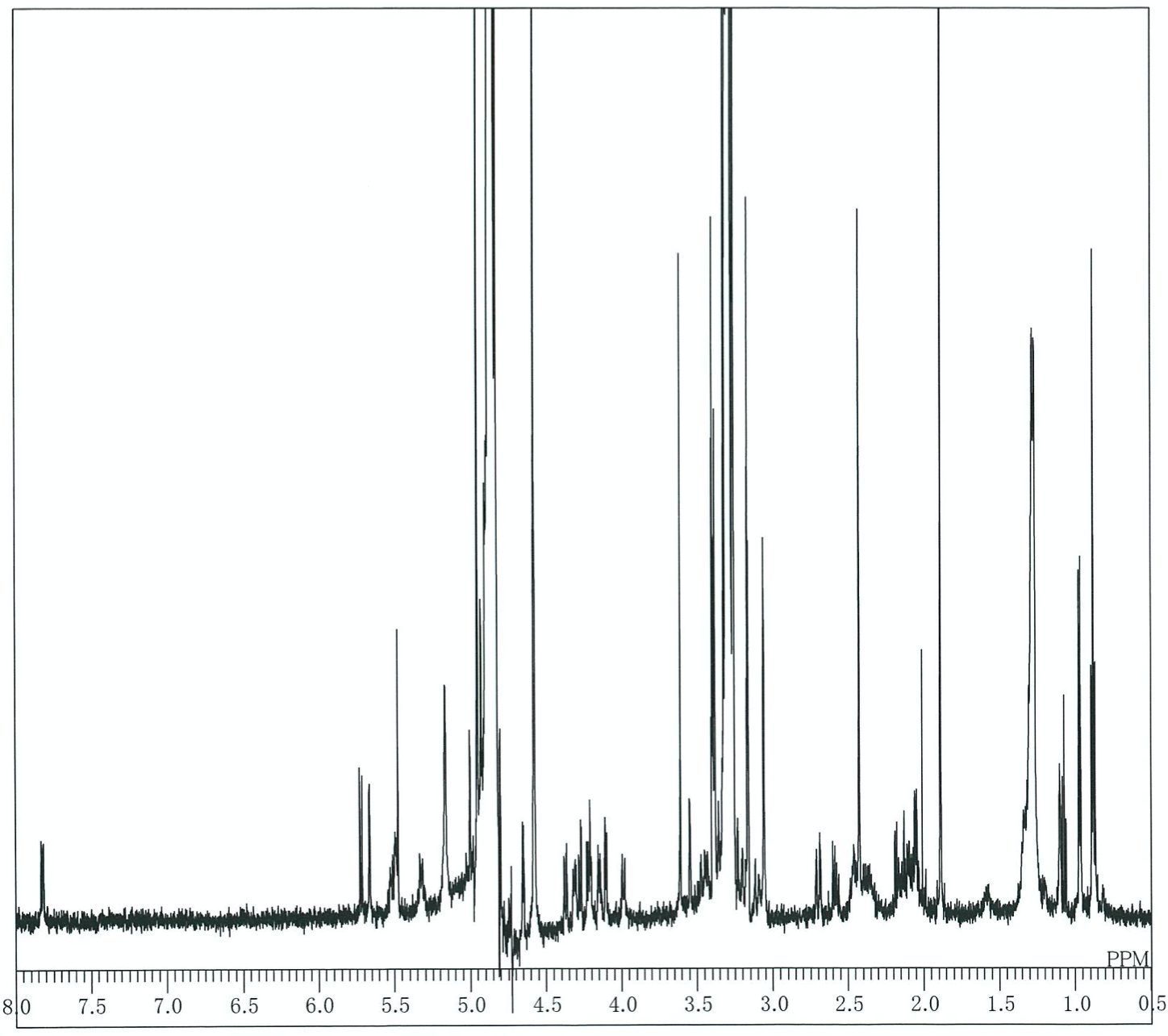


Figure S31 ^1^H NMR spectrum of **7** (600 MHz, CD_3_OD)


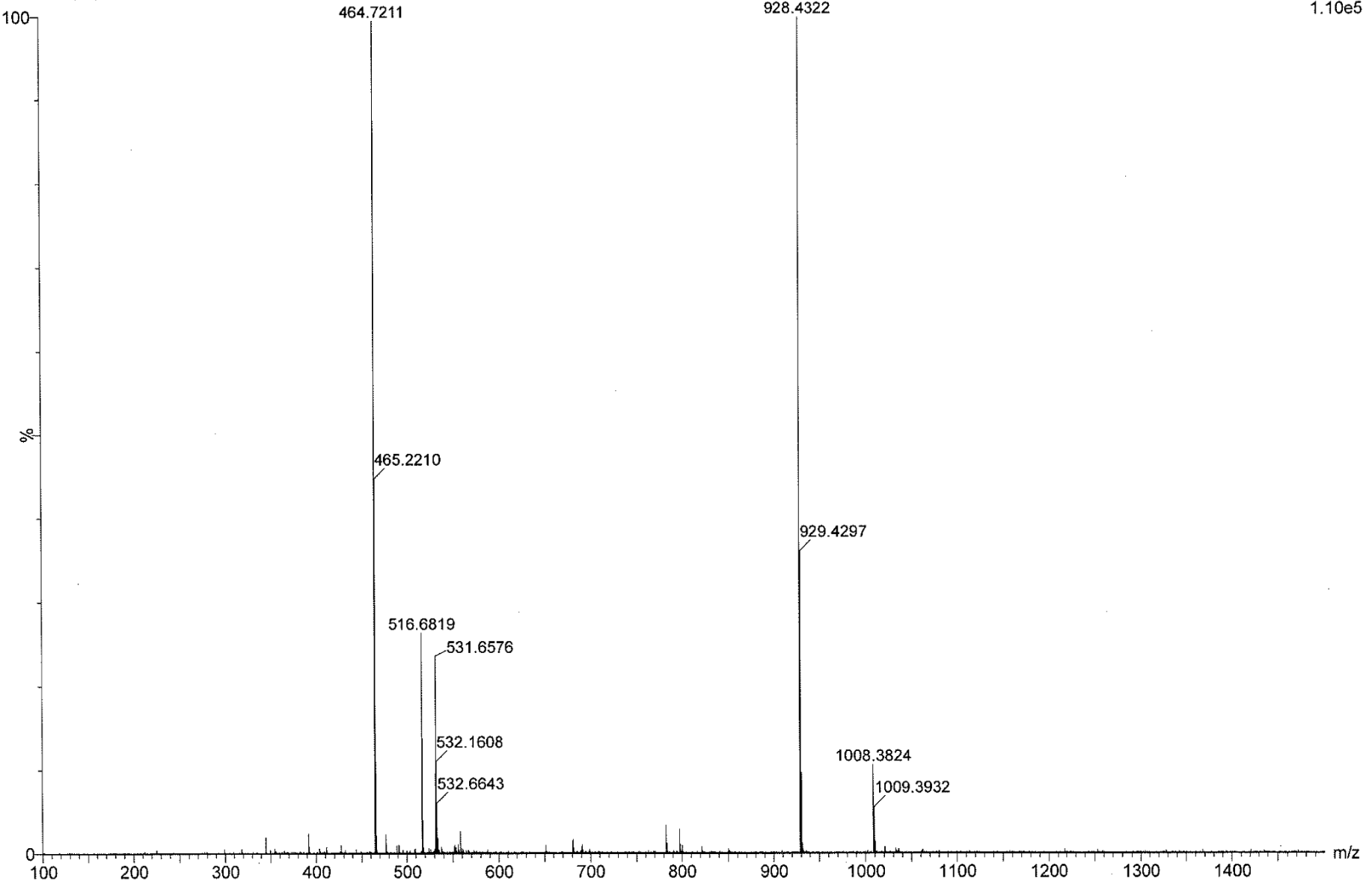


[M + H]^+^

Figure S32 HRESIMS spectrum of **7**

Figure S33 UV spectrum of **7**


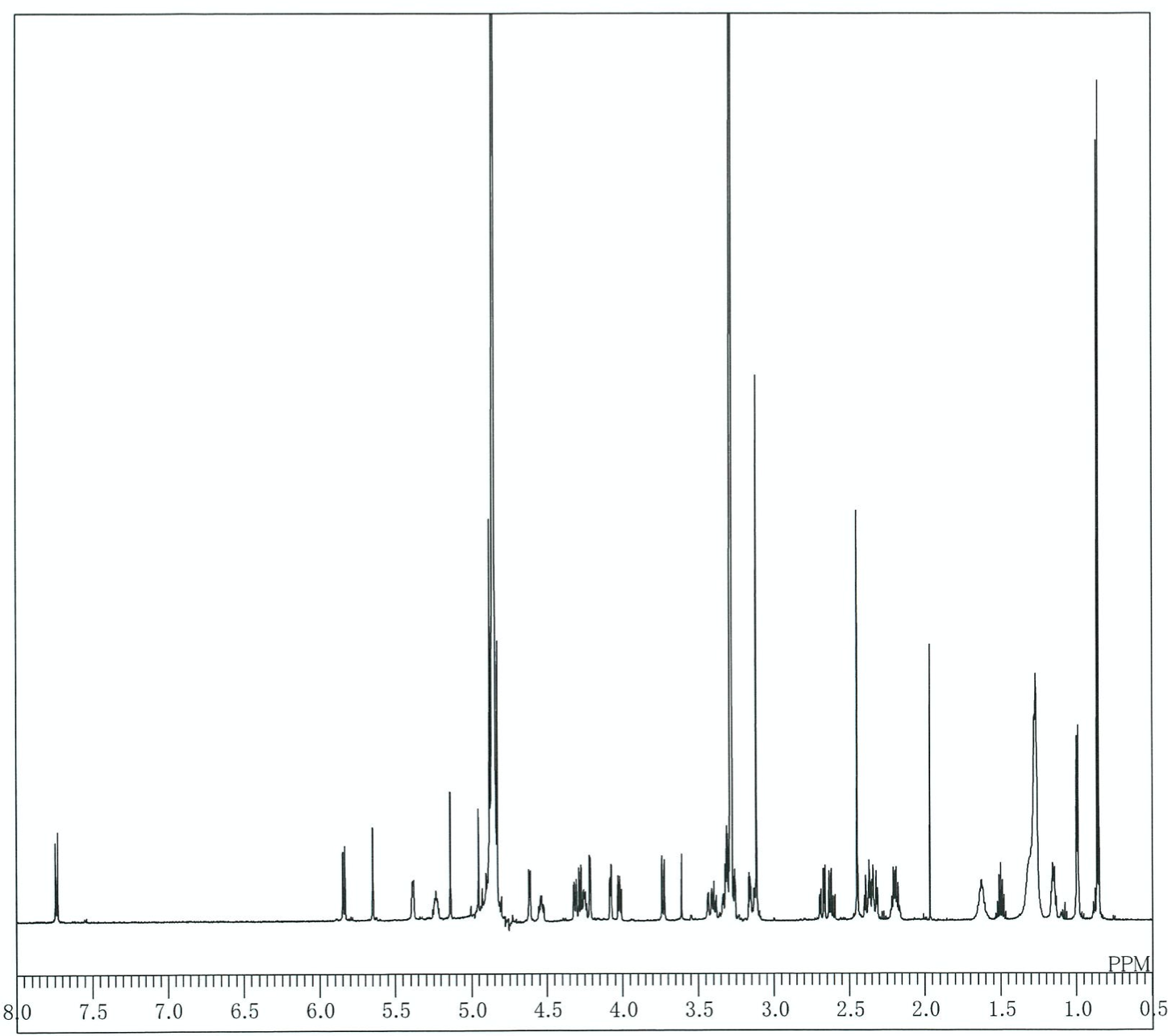


Figure S34 ^1^H NMR spectrum of **8** (600 MHz, CD_3_OD)


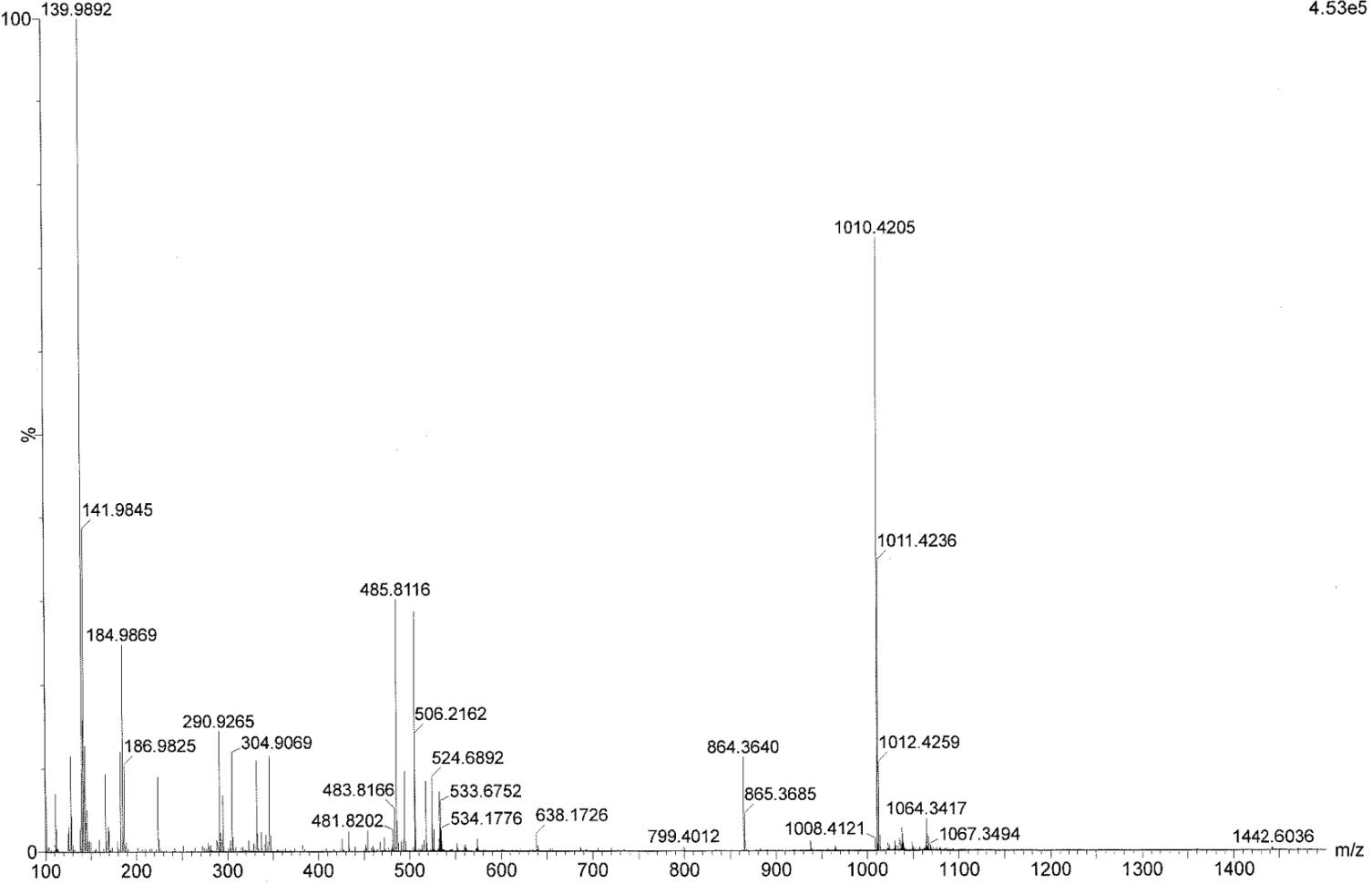


[M + H]^+^

Figure S35 HRESIMS spectrum of **8**

Figure S36 UV spectrum of **8**


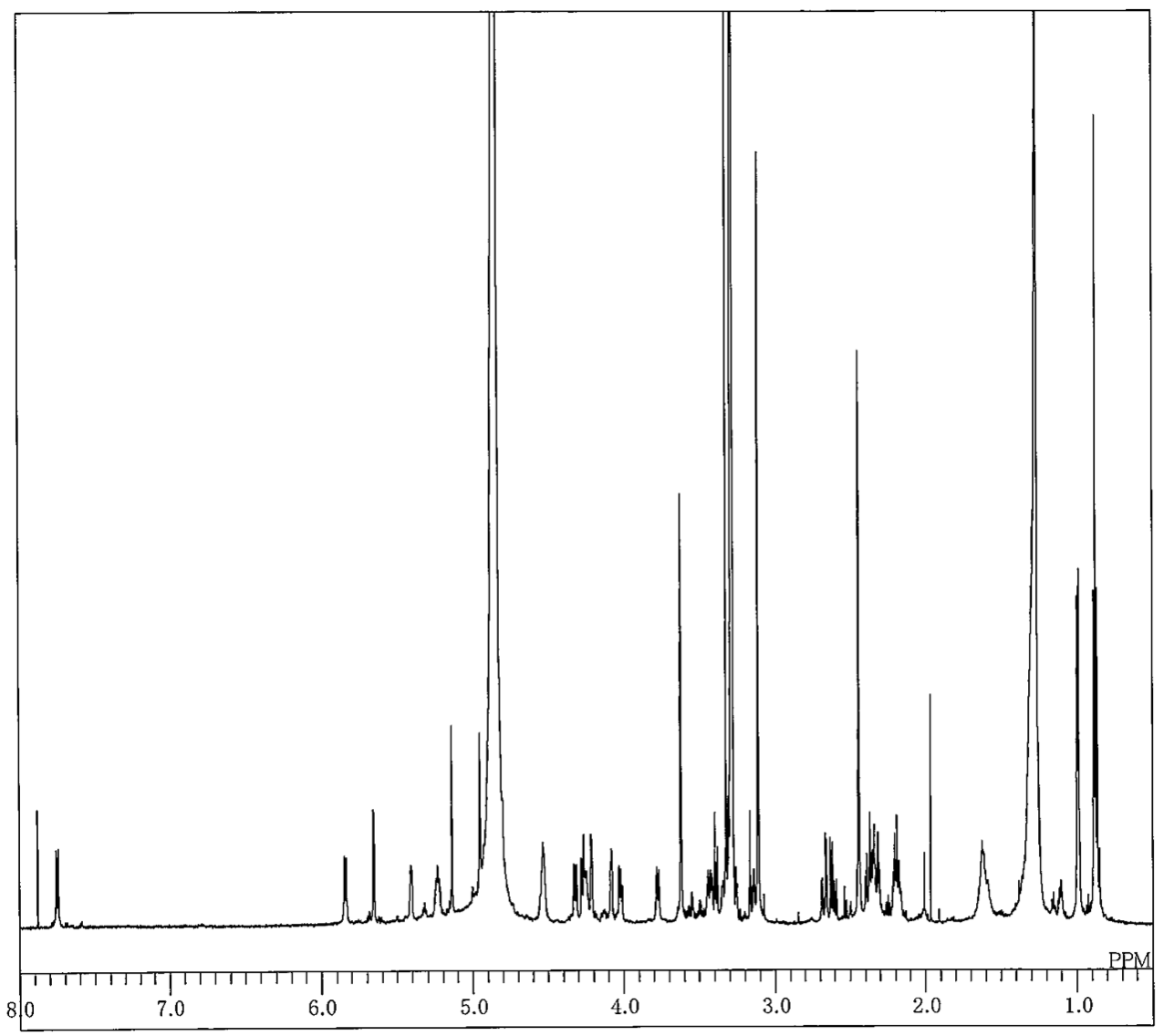


Figure S37 ^1^H NMR spectrum of **9** (600 MHz, CD_3_OD)


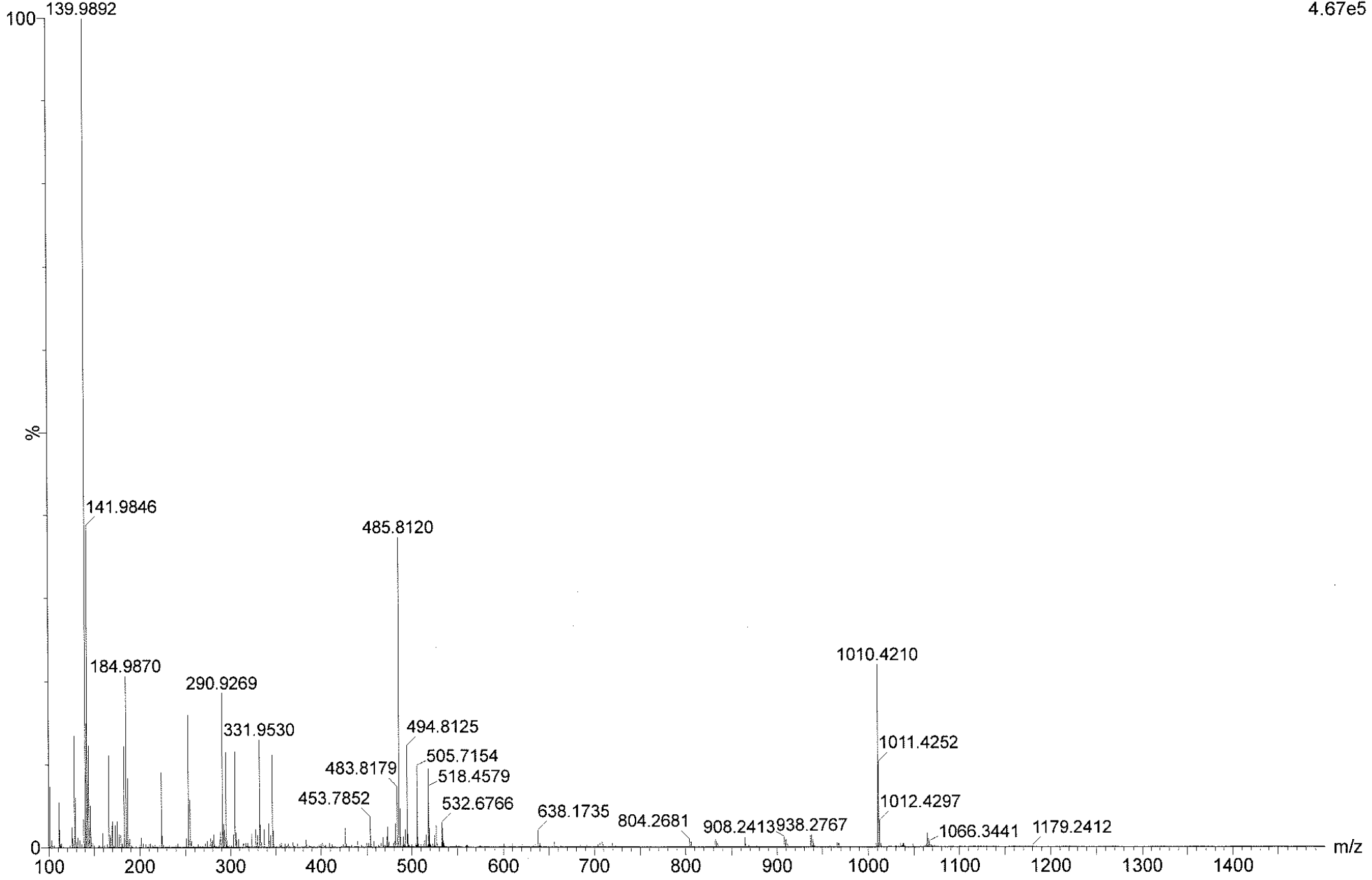


[M + H]^+^

Figure S38 HRESIMS spectrum of **9**

Figure S39 UV spectrum of **9**


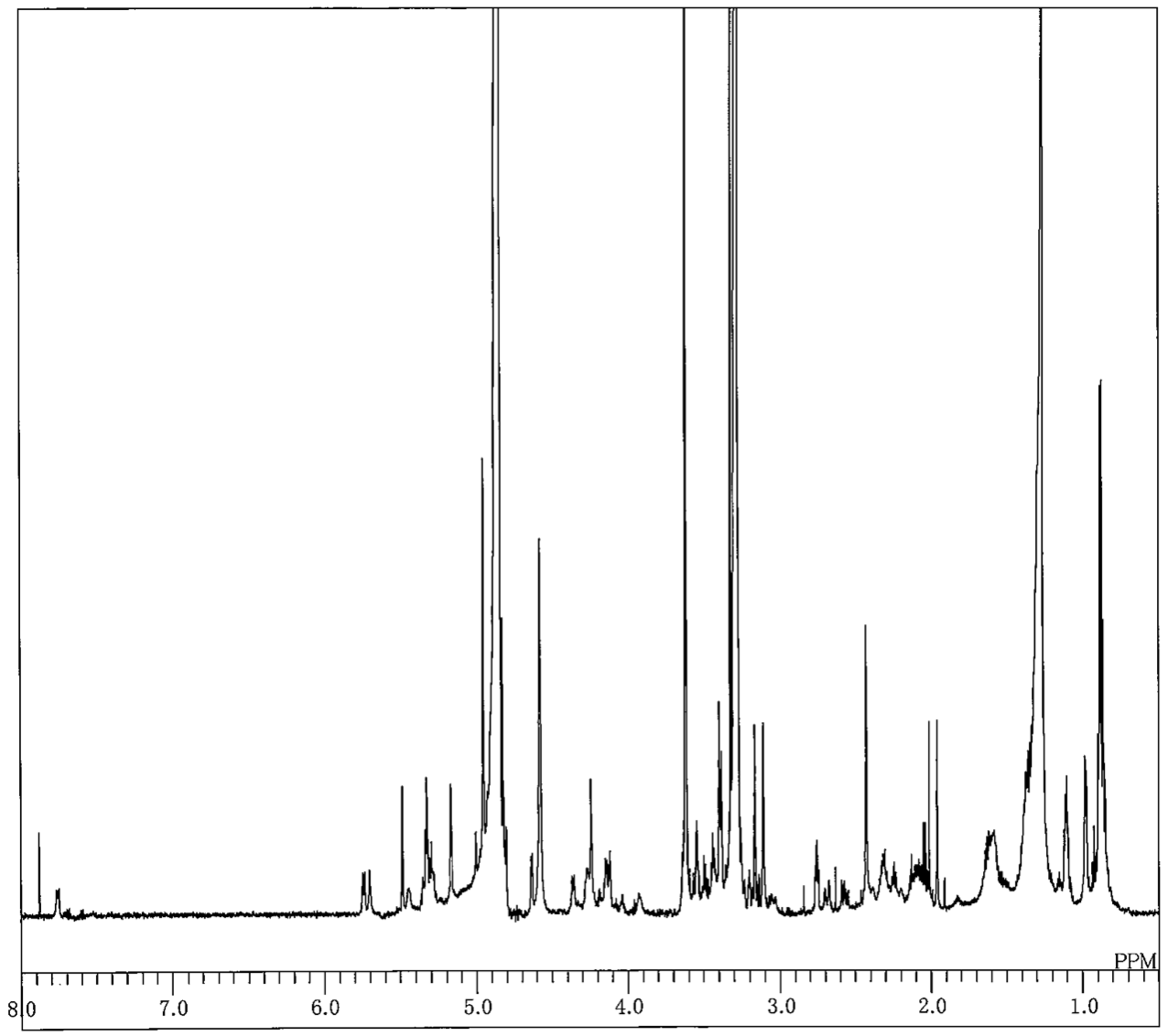


Figure S40 ^1^H NMR spectrum of **10** (600 MHz, CD_3_OD)


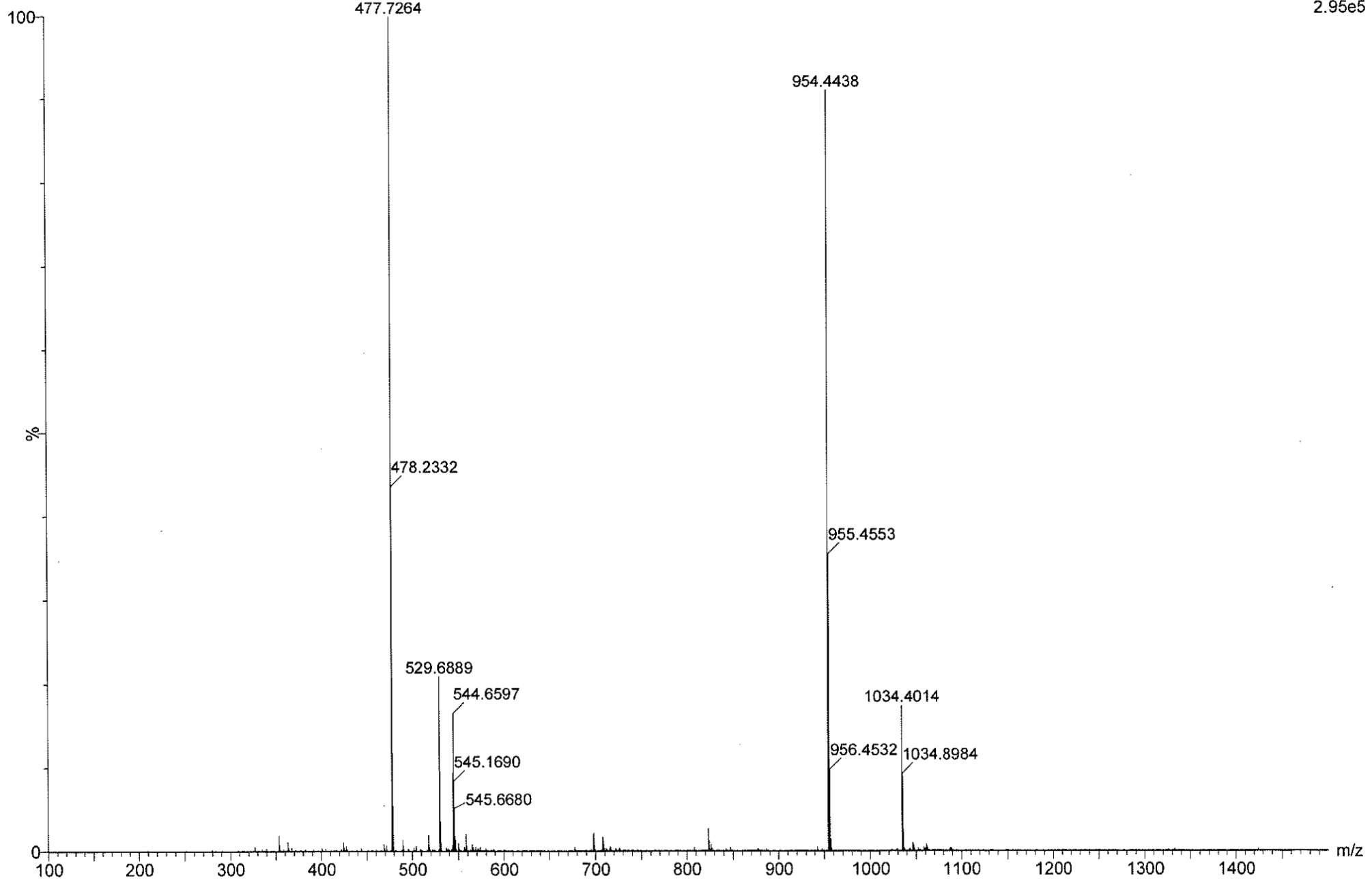


[M + H]^+^

Figure S41 HRESIMS spectrum of **10**

Figure S42 UV spectrum of **10**


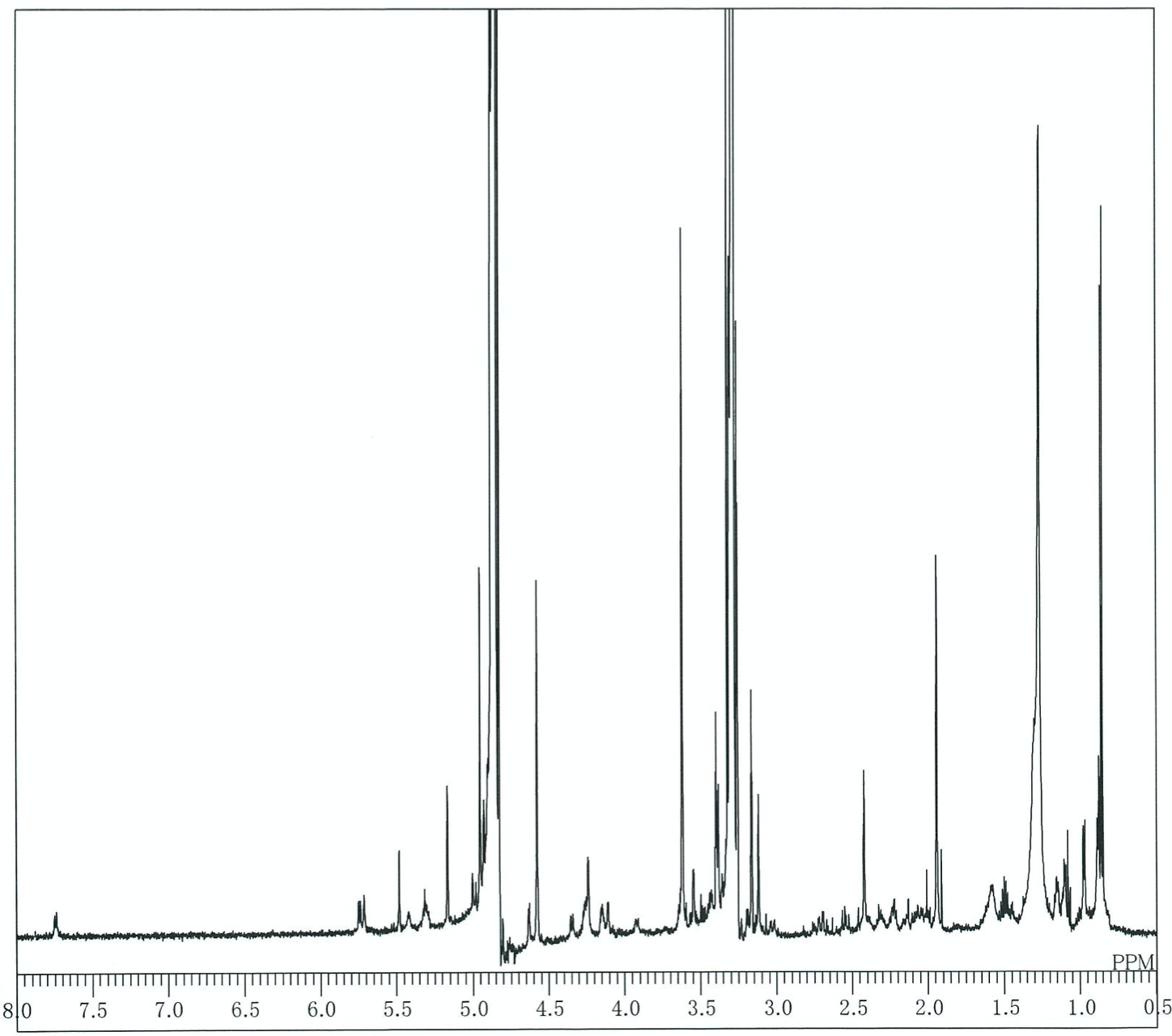


Figure S43 ^1^H NMR spectrum of **11** (600 MHz, CD_3_OD)


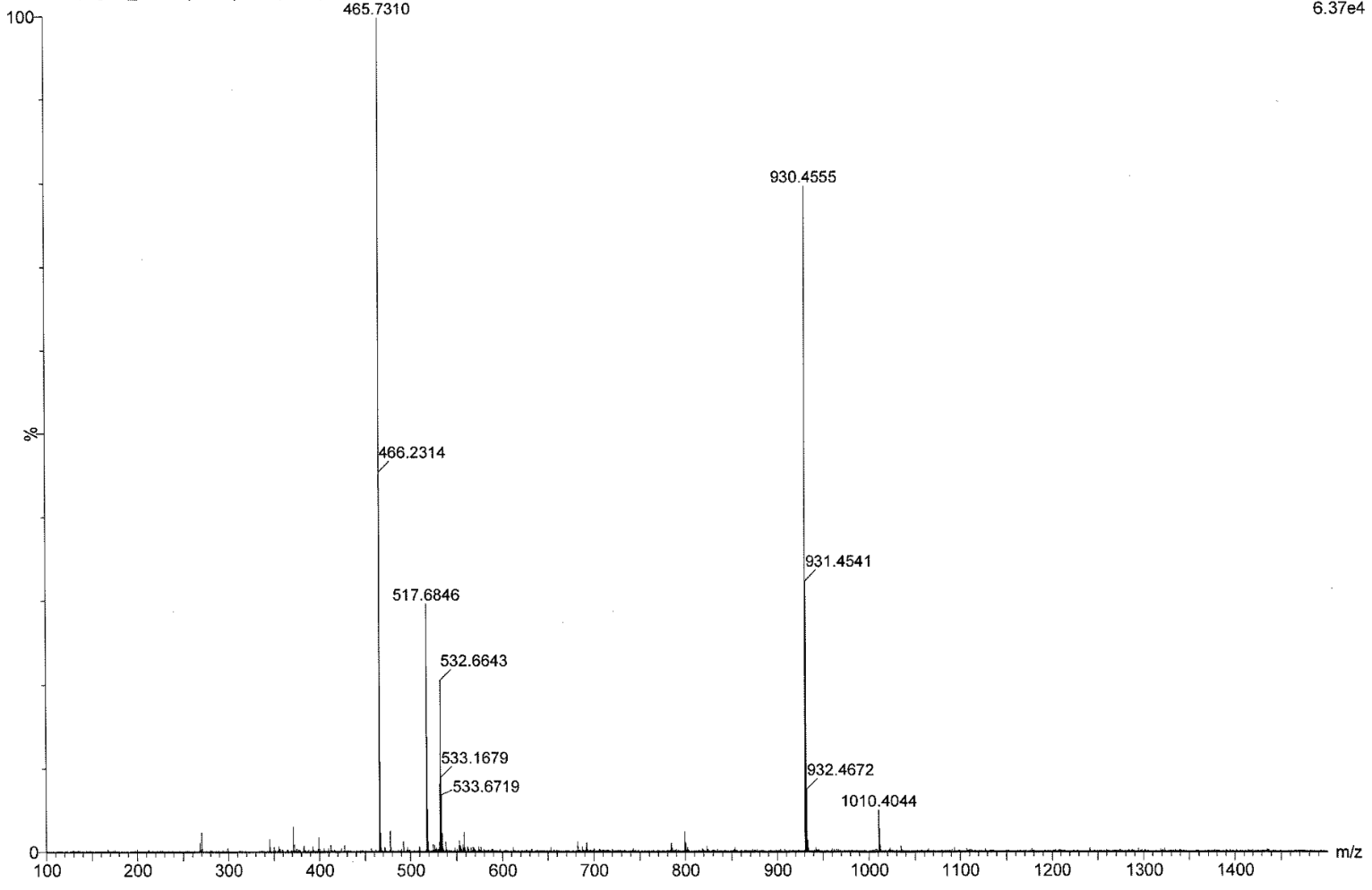


[M + H]^+^

Figure S44 HRESIMS spectrum of **11**

Figure S45 UV spectrum of **11**


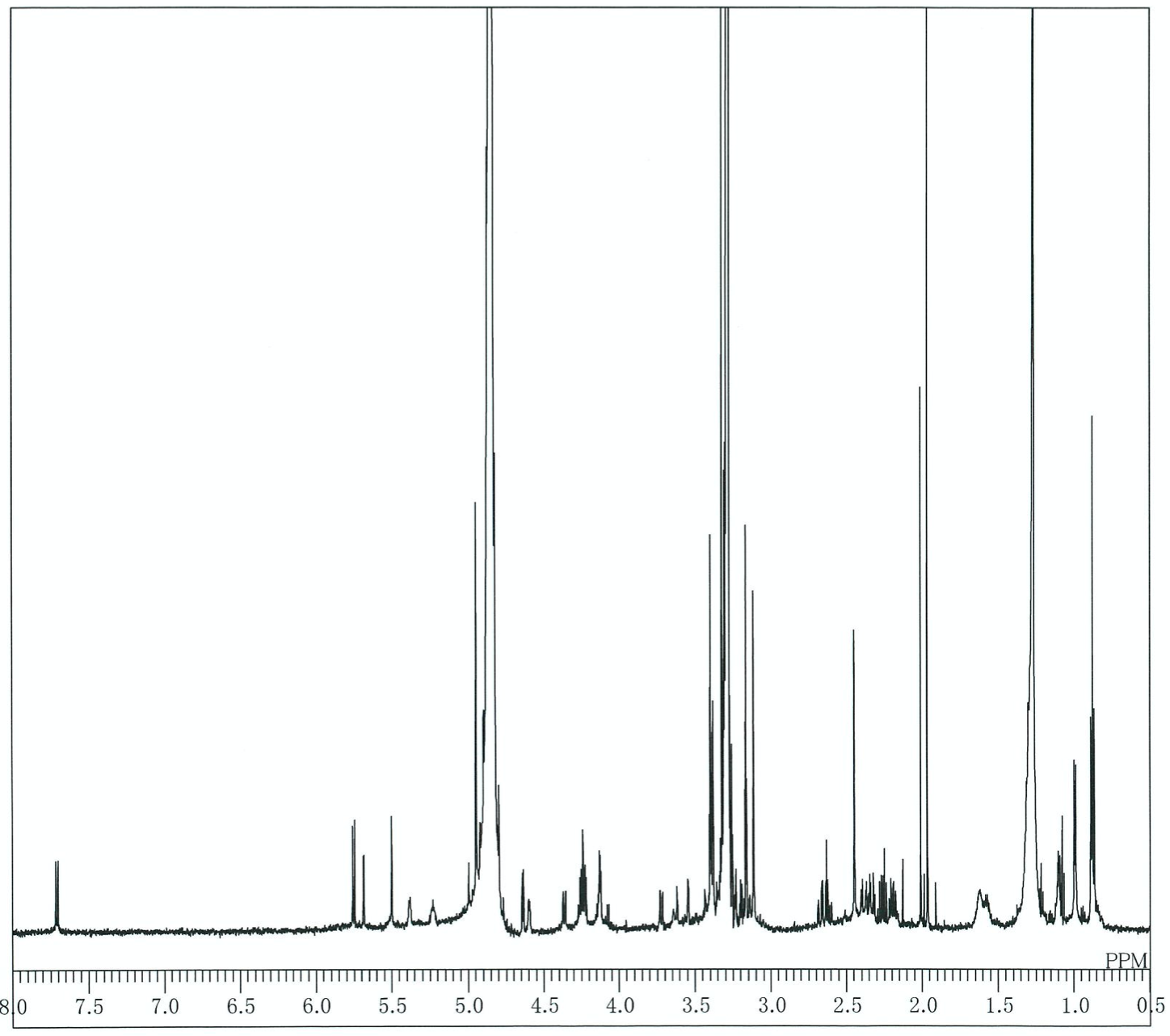


Figure S46 ^1^H NMR spectrum of **12** (600 MHz, CD_3_OD)


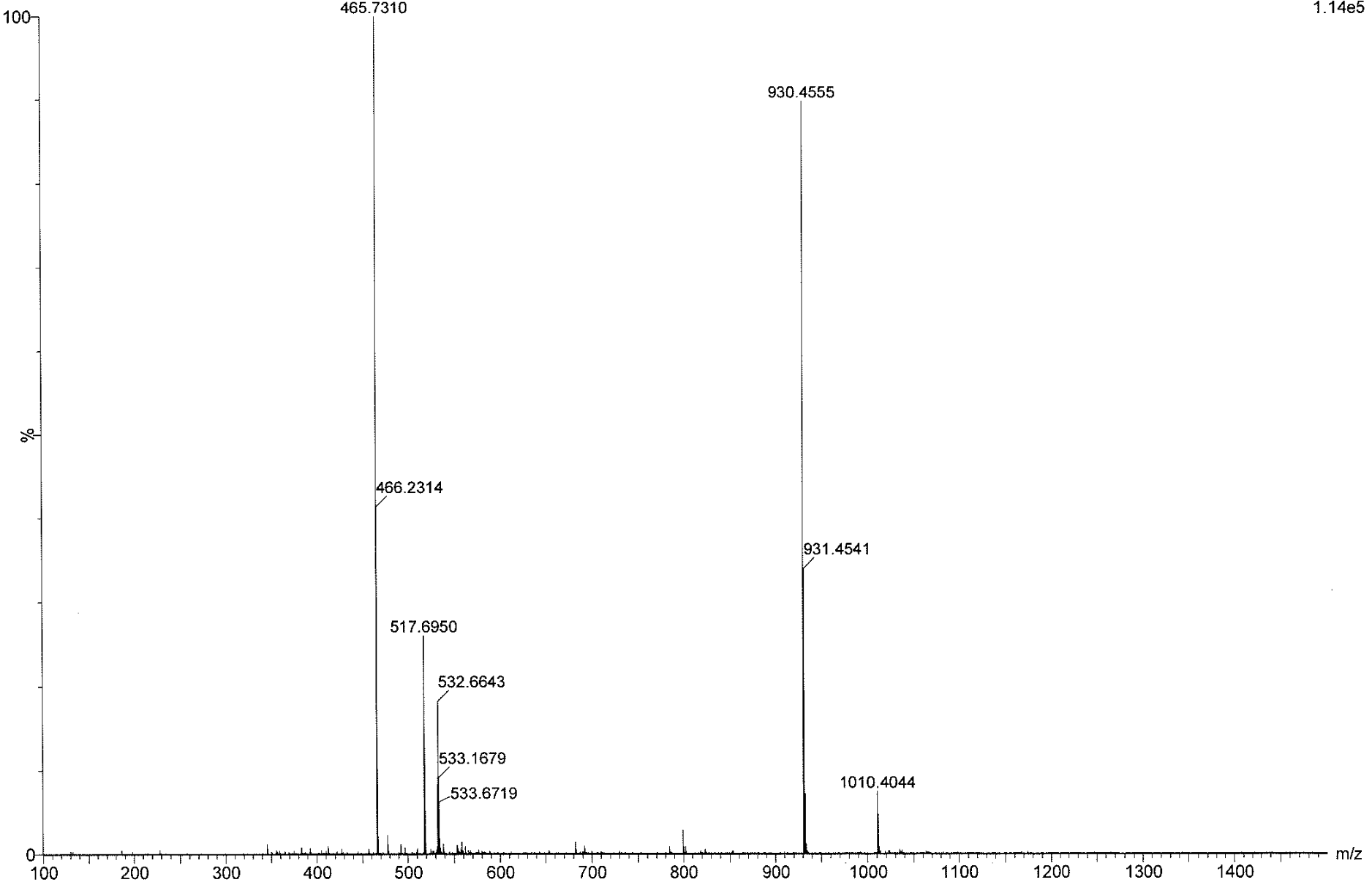


[M + H]^+^

Figure S47 HRESIMS spectrum of **12**

Figure S48 UV spectrum of **12**


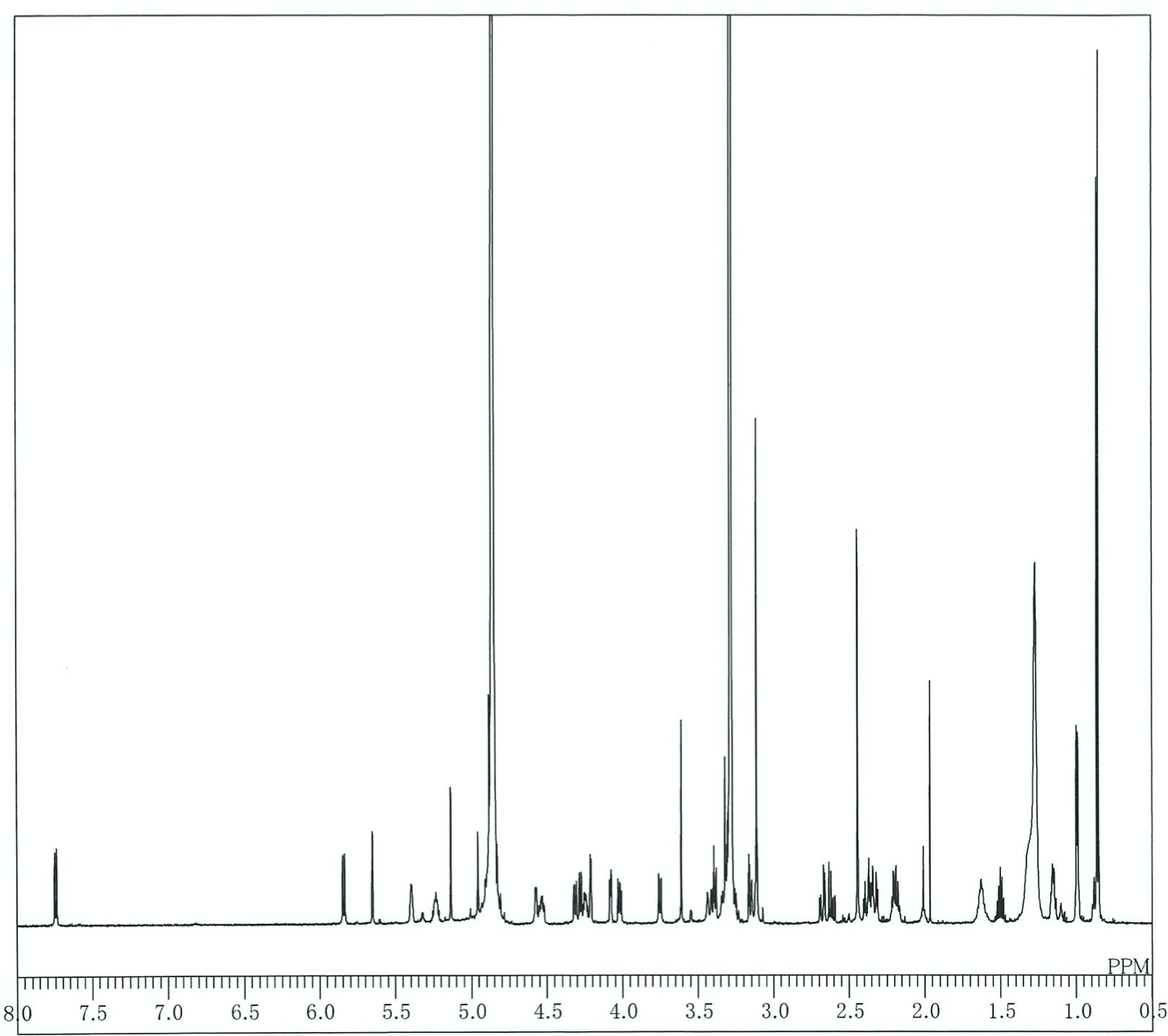


Figure S49 ^1^H NMR spectrum of **13** (600 MHz, CD_3_OD)


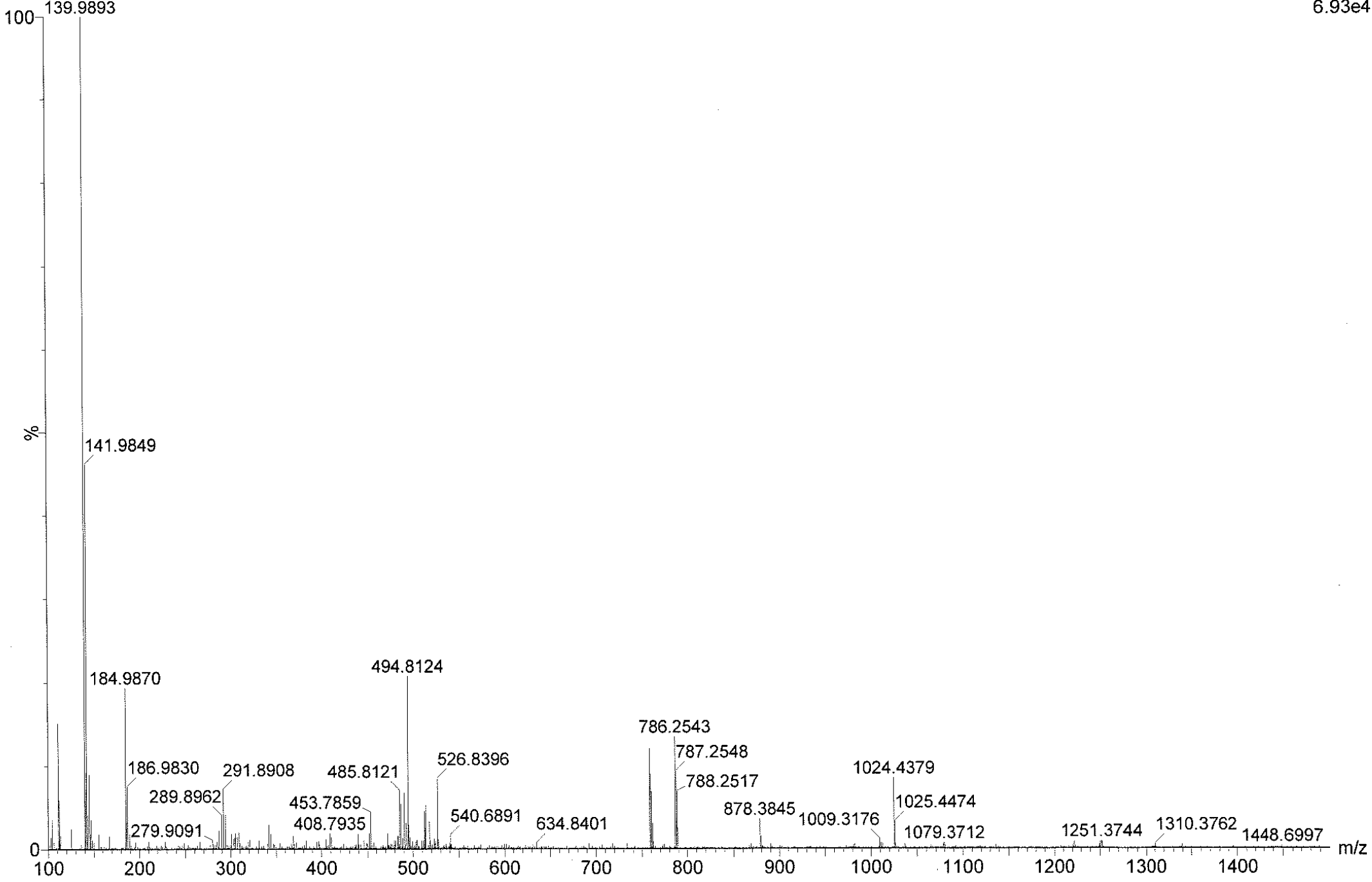


[M + H]^+^

Figure S50 HRESIMS spectrum of **13**

Figure S51 UV spectrum of **13**


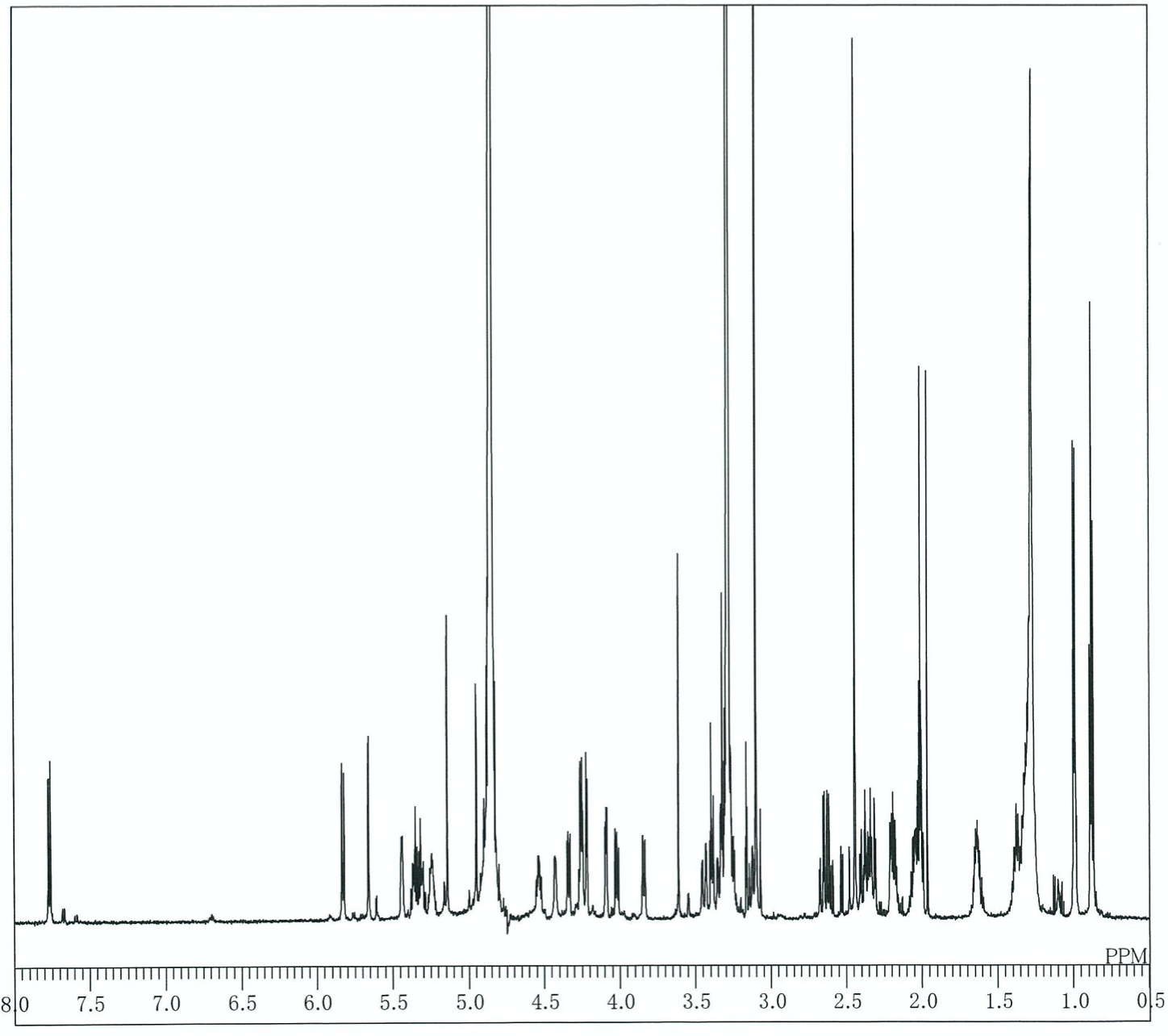


Figure S52 ^1^H NMR spectrum of **14** (600 MHz, CD_3_OD)


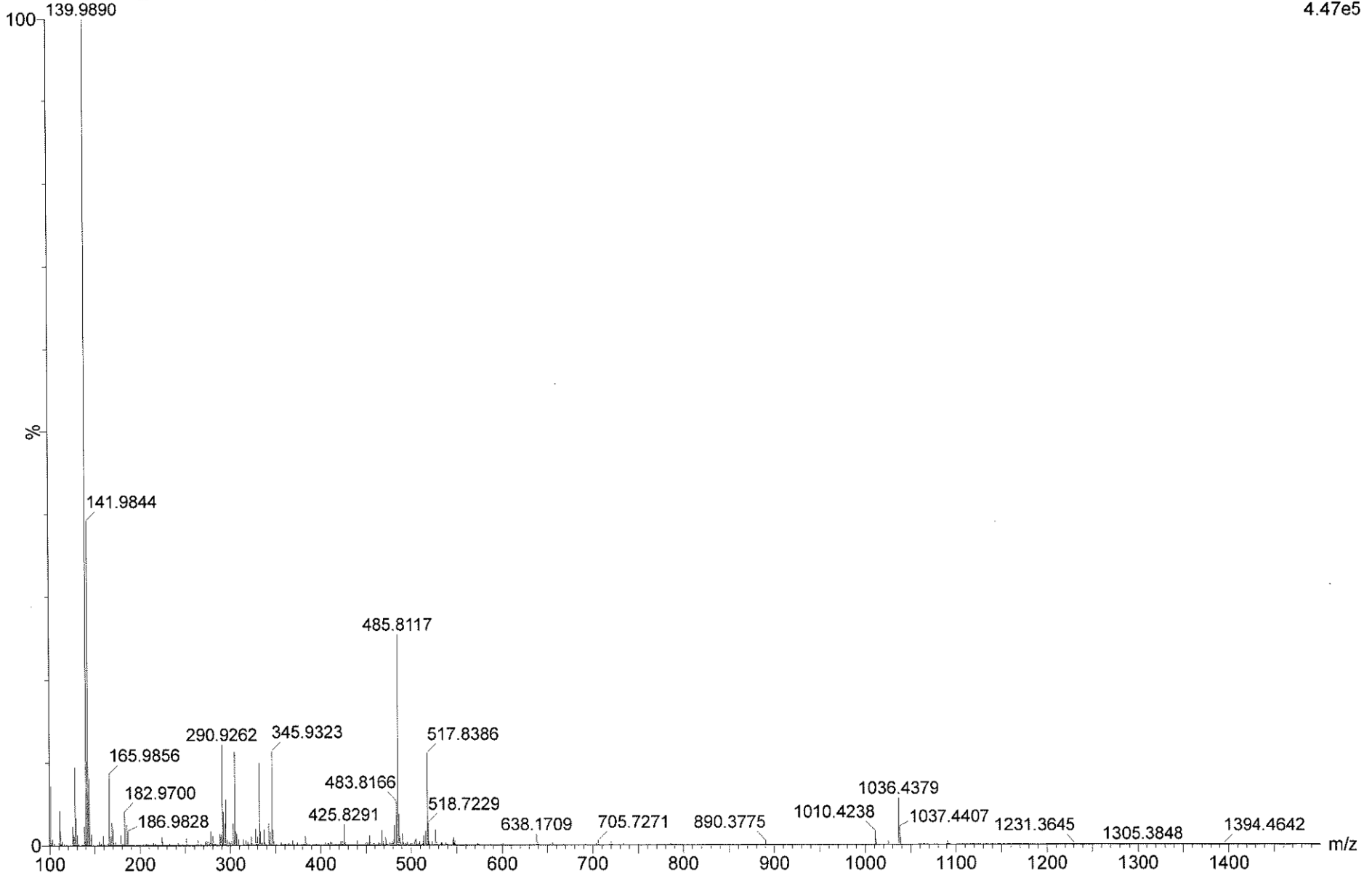


[M + H]^+^

Figure S53 HRESIMS spectrum of **14**

Figure S54 UV spectrum of **14**


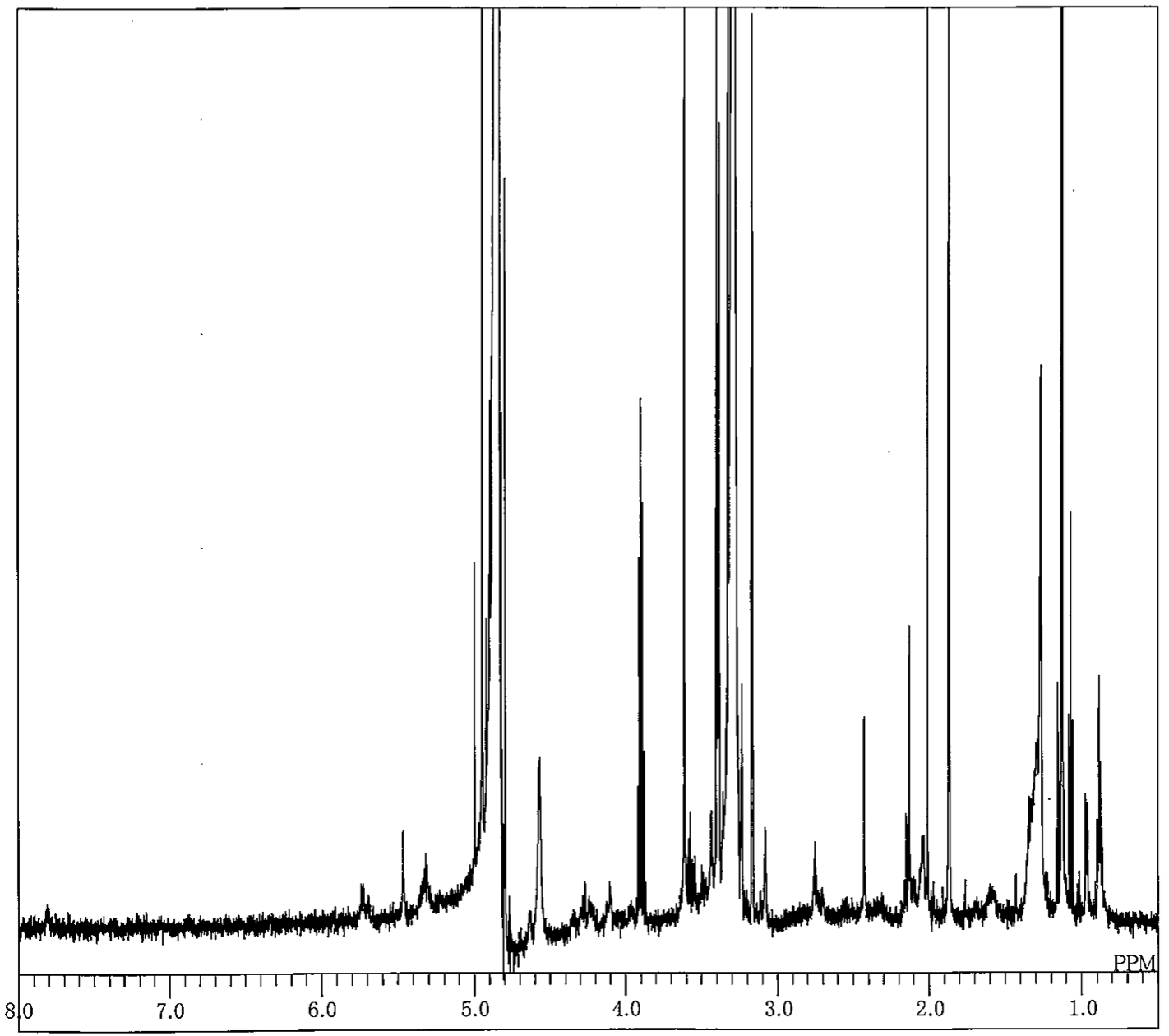


Figure S55 ^1^H NMR spectrum of **15** (600 MHz, CD_3_OD)


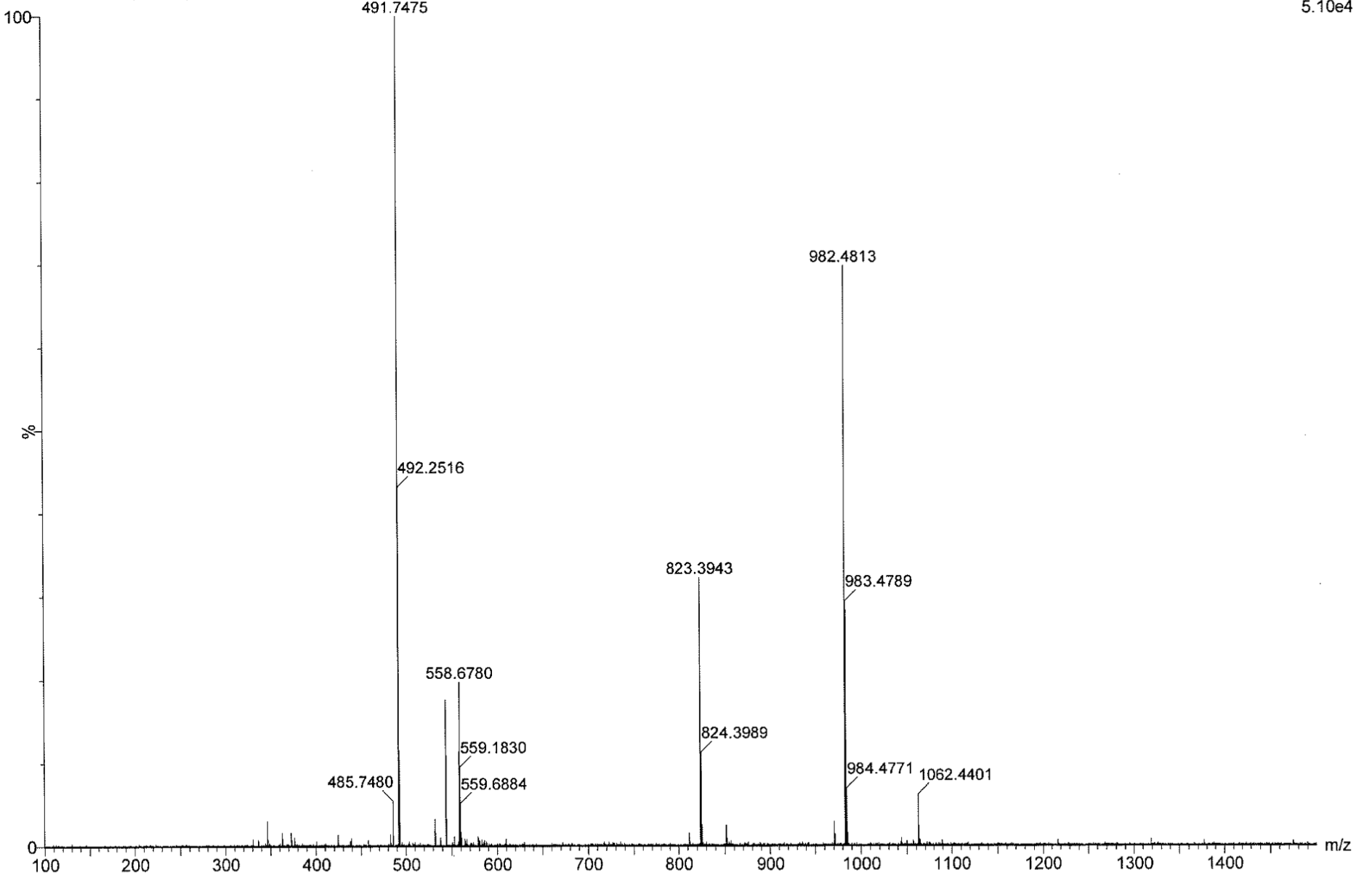


[M + H]^+^

Figure S56 HRESIMS spectrum of **15**

Figure S57 UV spectrum of **15**


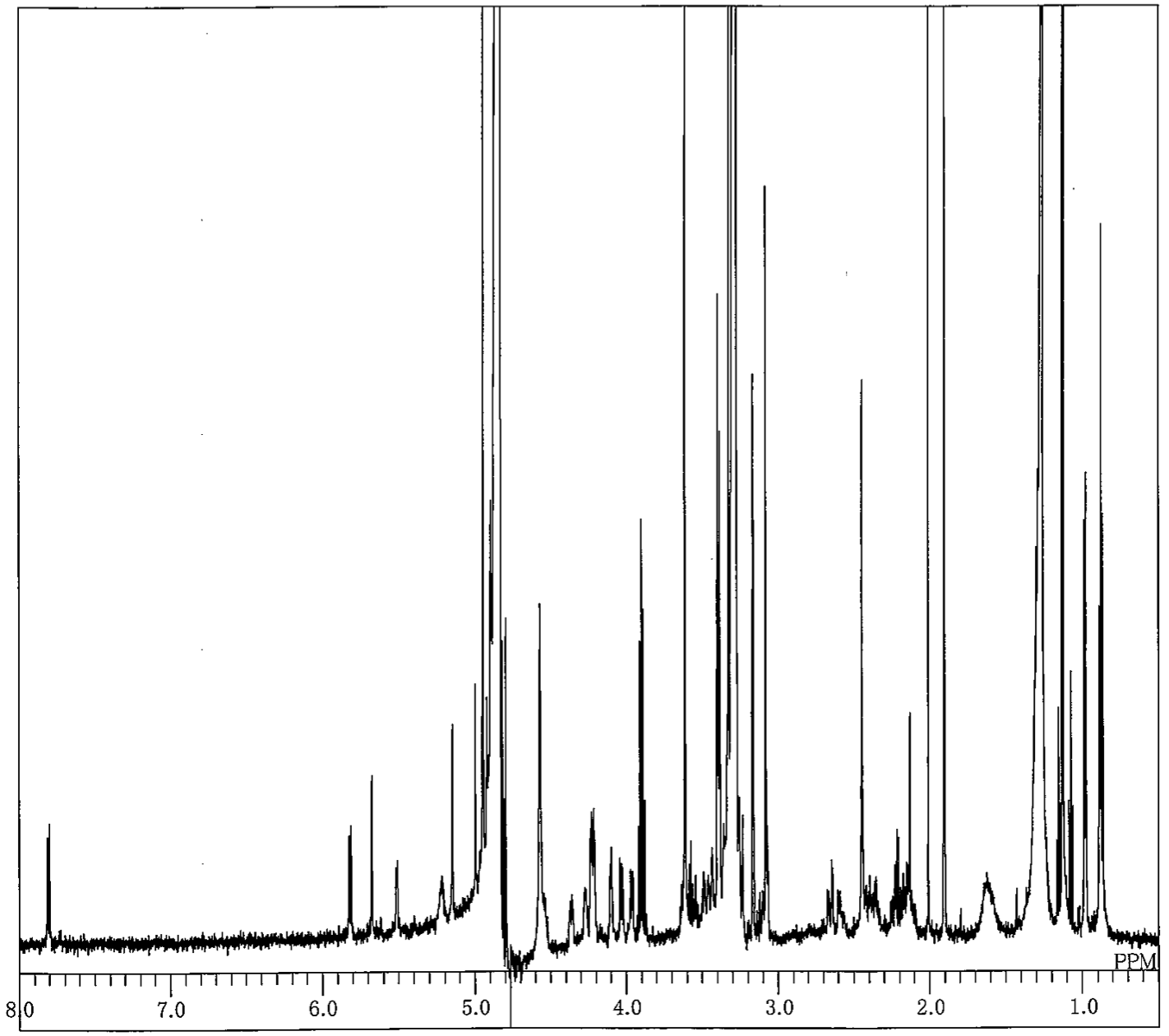


Figure S58 ^1^H NMR spectrum of **16** (600 MHz, CD_3_OD)


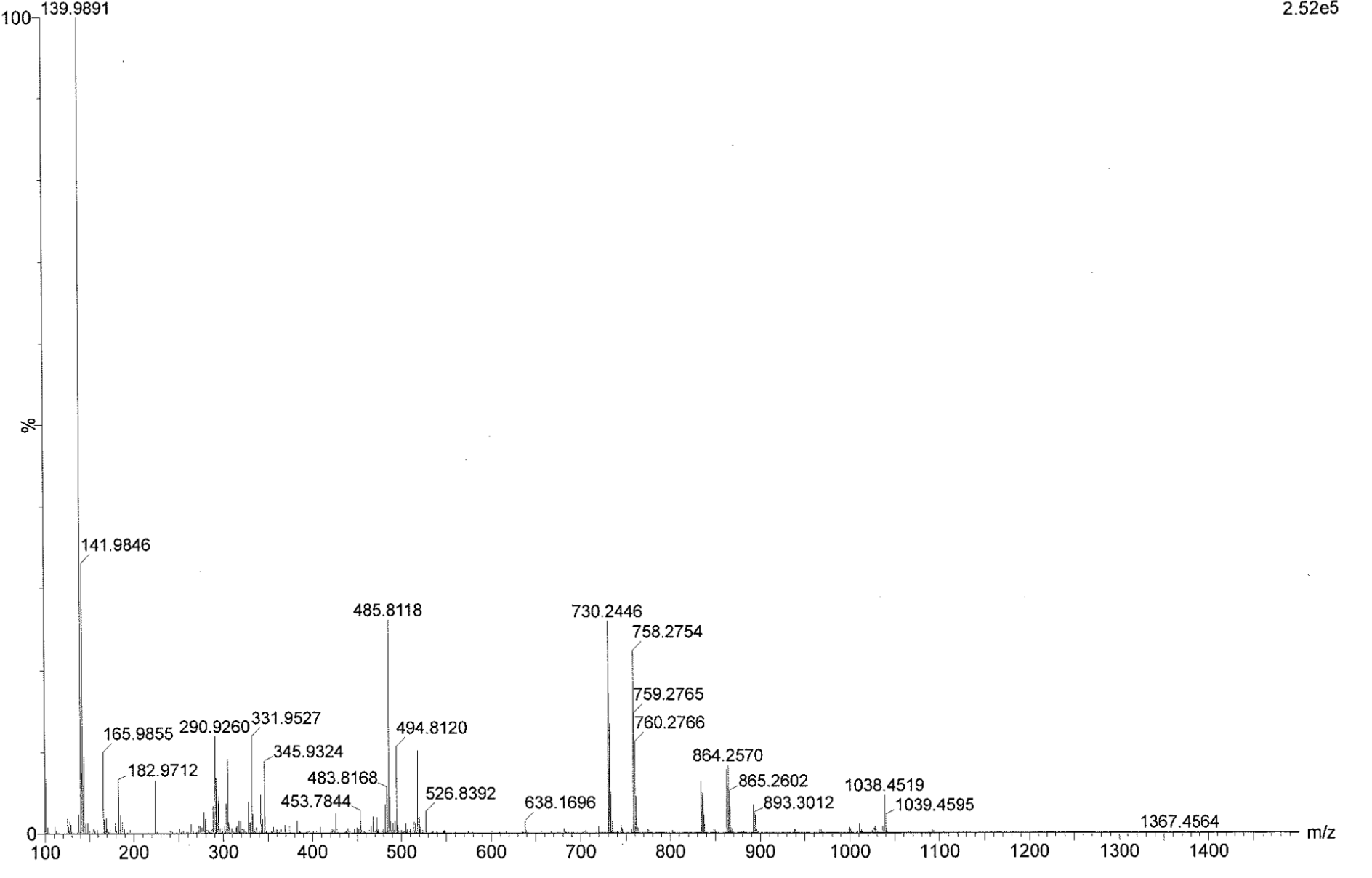


[M + H]^+^

Figure S59 HRESIMS spectrum of **16**

Figure S60 UV spectrum of **16**


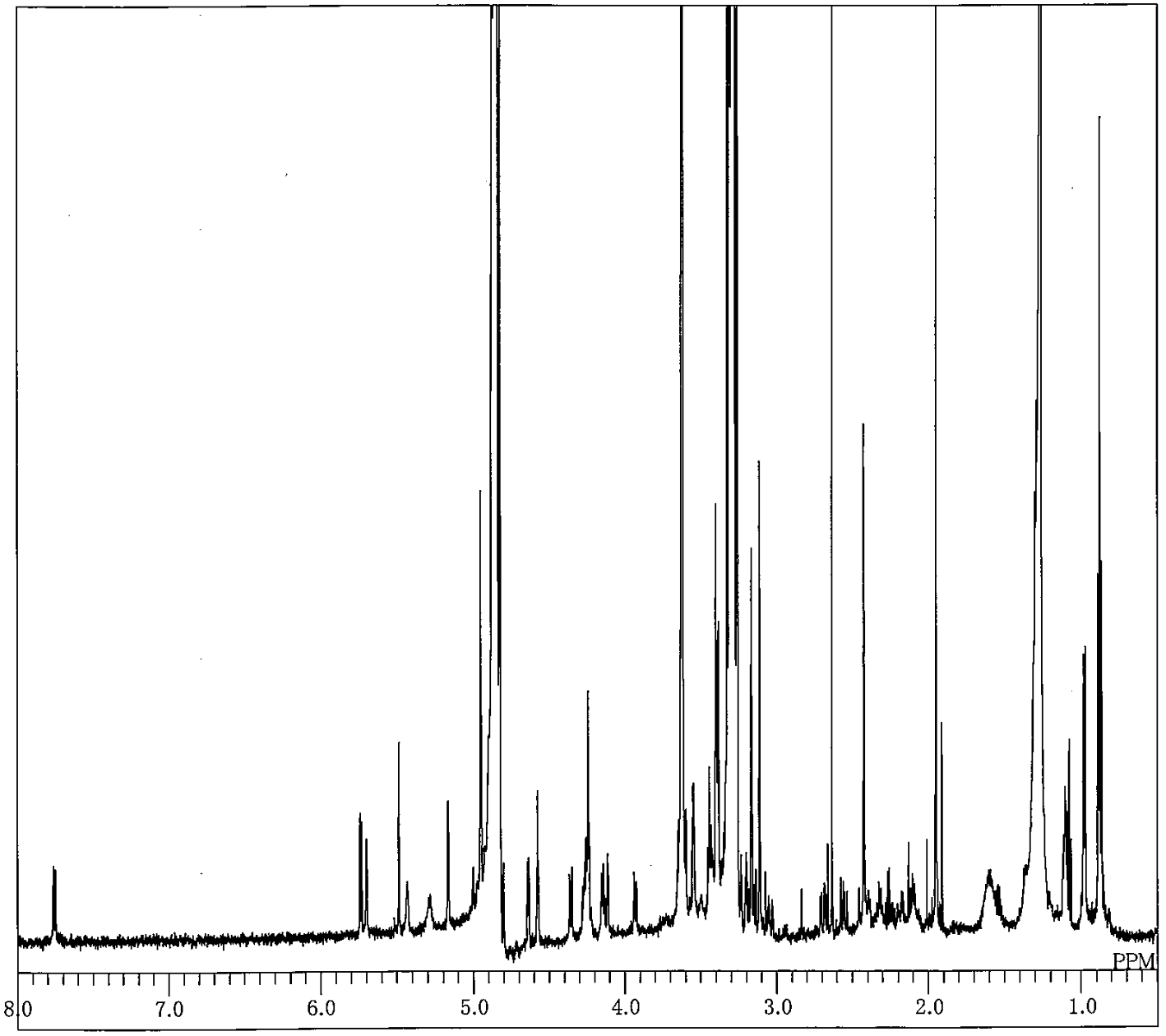


Figure S61 ^1^H NMR spectrum of **17** (600 MHz, CD_3_OD)


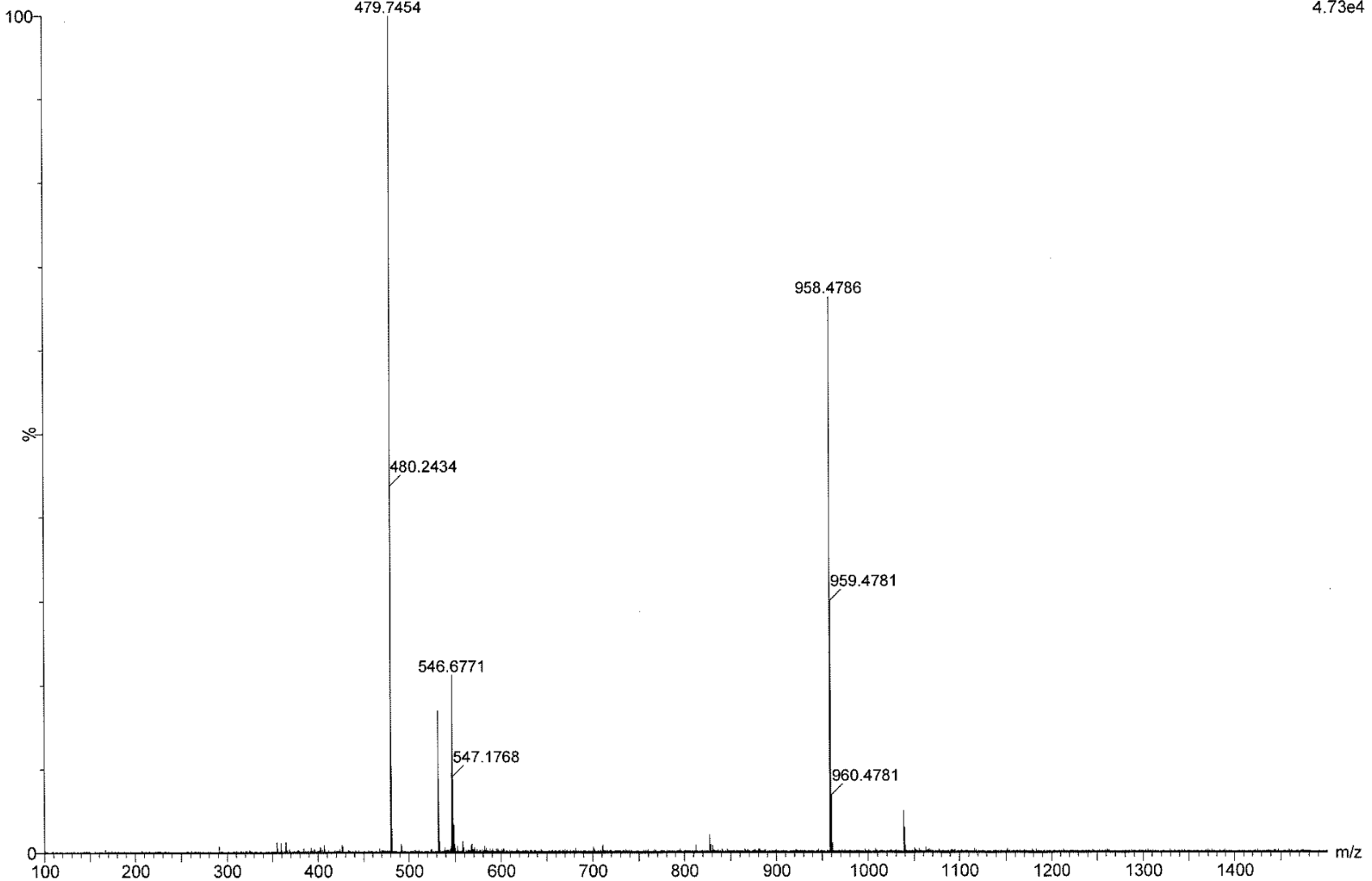


[M + H]^+^

Figure S62 HRESIMS spectrum of **17**

Figure S63 UV spectrum of **17**
